# Molecular Mimics of Intermetallic Phases: Selective Alkylamide‐Ligand Deprotection Drives Co/Ga Cluster Formation

**DOI:** 10.1002/anie.202523687

**Published:** 2026-03-13

**Authors:** Fabrizio E. Napoli, Raphael Bühler, Johannes Stephan, Samia Kahlal, Jean‐Yves Saillard, Christian Gemel, Roland A. Fischer

**Affiliations:** ^1^ Department of Chemistry Technical University of Munich, TUM School of Natural Sciences, Chair of Inorganic and Metal‐Organic Chemistry Garching Germany; ^2^ Catalysis Research Center Technical University of Munich Garching Germany; ^3^ Univ Rennes, CNRS, ISCR‐UMR Rennes France

**Keywords:** alloyed clusters, cluster compounds, cluster hydrogenation, metal co‐reduction, synthetic methods

## Abstract

The heterobimetallic M_14_ cluster [Co_3_Ga_2_]H(μ^2^‐GaTMP)_9_ (**1**, TMP = 2,2,6,6‐tetramethylpiperidinyl) was obtained by treating CoCl_2_ with GaTMP in the presence of Mg and H_2_ as reduction additives. **1** features a trigonal bipyramidal Co_3_Ga_2_ kernel with one substituent‐free Ga atom and one exposed GaH moiety inside a metallo‐environment of nine edge‐bridging GaTMP ligands. The resulting Co_3_Ga_11_ cluster core mimics a packing motif of the structurally related intermetallic phase Co_2_Al_5_. The GaTMP ligands not only donate their lone pairs to the Co, but also to the exposed, bare Ga of the Co_3_Ga_2_ kernel, thus contributing to the increase in delocalization over the M_14_ core, according to bonding analysis. The synthesis of **1** represents proof‐of‐concept to steer the coordination of GaTMP to transition metal centers toward a kernel‐alloyed Co/Ga cluster formation. This is rationalized by Mg/H_2_‐induced Ga deprotection and TMP‐trapping in the *Hauser*‐base complex [Mg(TMP)(THF)μ^2^Cl]_2_. The method bears potential for generalization, and first results were obtained for related Fe/Ga clusters.

## Graphical abstract

The bimetallic M_14_ cluster [Co_3_Ga_2_]H(μ^2^‐GaTMP)_9_ was achieved by a co‐reductive approach utilizing Mg and H_2_ to selectively deprotect and capture the Ga‐TMP metallo‐ligands, forming a Co/Ga alloyed cluster kernel, making it a structural representative of a related solid‐state counterpart. The role of the additives in this system has been rationalized, and the system was applied to yield structurally similar Fe/Ga clusters and complexes.

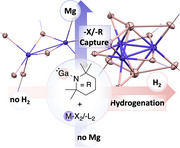

## 1 Introduction

Intermetallic compounds have been recognized as promising materials for modulating the proximate environment of catalytically active metal sites [1, 2]. Heterometallic clusters can mimic the structural motifs and reactivity patterns of their solid‐state counterparts at the molecular level [3, 4, 5, 6, 7].

An example is the decanuclear cluster [Ni_3_(GaTMP)_7_] (TMP = 2,2,6,6‐tetramethylpiperidinyl), which activates dihydrogen and catalyzes alkyne semihydrogenation in a cooperative fashion of Ni and Ga sites similar to the active site of the structurally related Ni_5_Ga_3_ solid‐state phase [8]. The chemical space of related intermetallics is enormous, and likewise for related clusters. It offers a vast, largely unexplored field for the discovery of novel structure–property relationships, in particular at cluster size regimes below 1 nm, where every metal atom counts [9]. The controlled formation of heterometallic clusters with alloyed cores, especially those involving chemically very dissimilar elements such as transition (M) and main group metals (E), remains a fundamental challenge. The synthetic obstacle lies in their differing redox properties. While co‐reduction is a common route to bimetallic clusters with chemically closely related metals (e.g., Cu, Ag, and Au) [10, 11, 12, 13, 14, 15, 16], the disparate reduction potentials and kinetics of E and M render this strategy difficult. To circumvent this, low‐valent organometallic compounds of main group elements, such as ER* (E = Al, Ga, In; R* = bulky organic substituents), have been employed as dual functional agents, acting as reducing agents and simultaneously as metallo‐ligands [3, 17, 18]. Previously, we preferred Cp* (C_5_Me_5_), due to its flexible binding mode allowing for deprotection of E by Cp* cleavage involving hydrogenolysis [19], protolysis [20], and one‐electron oxidation [21]. However, a limitation is the Cp* transfer from E to M (transmetallation), favored by strong Cp* binding to electron‐deficient M centers (e.g., Fe, Co, and Ni) [22, 23, 24, 25, 26]. Consequently, many clusters of the formula [M_a_E_b_](Cp*)_c_ (c<a + b; c = a or c = b) feature segregation of M and E, where E forms the kernel and M reside at the periphery and are ligated by Cp*, or vice versa, rather than forming alloyed kernels [M_a‐x_E_b‐y_](Cp*)_c_ (c<a + b; c<a, c<b), embedded into a metallo‐ligand shell [18, 27, 28, 29].

Herein, we report the synthesis and provide a discussion of the structure and bonding of the kernel‐alloyed M_14_ cluster [Co_3_Ga_2_]H(μ^2^‐GaTMP)_9_ (**1**) via a co‐reductive approach different from the typical methods (vide infra). The trigonal bipyramidal Co_3_Ga_2_ kernel of the M_14_ core is embedded into a shell of nine GaTMP ligands. The kernel features exposed Ga and Ga‐H moieties. The cluster mimics structural motifs of the Co_2_Al_5_ intermetallic phase. It was obtained by the treatment of CoCl_2_ and GaTMP with Mg and H_2_ as co‐reductants. We discovered decisive roles of Mg and H_2_ that favor Co/Ga‐alloyed cluster kernel formation by selective TMP‐deprotection and its trapping in the *Hauser*‐base complex [Mg(TMP)(THF)μ^2^‐Cl]_2_. We were able to show the transferability of the concept to Fe/Ga complexes and clusters.

## 2 Results and Discussion

**FIGURE 1:**
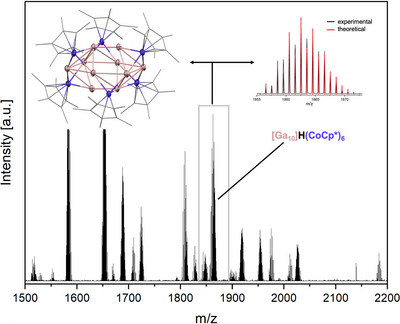
LIFDI mass spectrum of the [Ga_x_Co_y_](CoCp*)_z_ cluster library (with x = 5–11, y = 0,1 and z = 6,7). Top left: Molecular structure of the cluster [Ga_10_]H(CoCp*)_6_ (**2**) in the solid state (thermal ellipsoids given at 50% probability level, color code: cobalt: violet, gallium: rose). Top right: Mass spectrometric pattern of the molecular ion of **2** (*m/z* = 1862.5625, black), in accordance with the theoretically predicted pattern (calc.: 1862.56473, red).

Pursuing Co/Ga cluster synthesis by reduction of CoCl_2_ with Mg in the presence of GaCp* leads to an inseparable ensemble of several cluster species of the form [Ga_x_Co_y_](CoCp*)_z_ (with x = 5–11, y = 0,1 and z = 6,7) forming side by side (Figure 1 and Table S1), a Co/Ga cluster library similar to earlier works on Ni/Ga and Cu/Zn cluster libraries [27, 30].

The molecular structure of one of the cluster species, [Ga_10_]H(CoCp*)_6_ (**2**), was obtained by SC‐XRD, subsequent to crystal picking from the cluster ensemble. **2** depicts an M_16_ core, where a decanuclear gallium kernel is embedded into a Cp*‐ligated hexanuclear cobalt shell (Figure 1). This structure arises due to the transmetallation of Cp* from Ga to Co.

**FIGURE 2:**
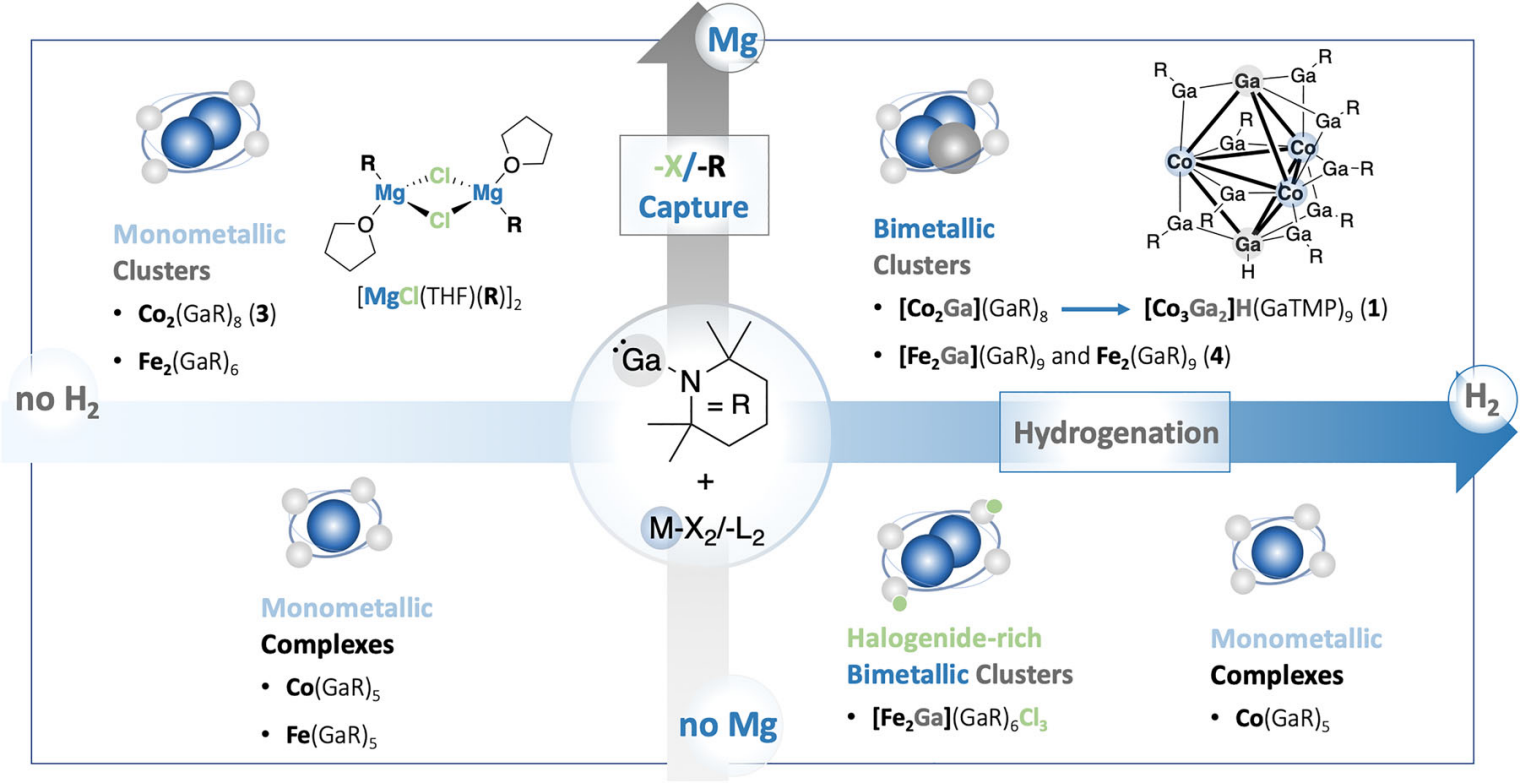
Synthetic concept for the formation of heterometallic Co/Ga and Fe/Ga complexes and clusters dependent on the reducing agent (Mg: reduction/capture of ligands/halogenides, H_2_: reduction/hydrogenation). In the segments: resulting types of formed species (bold) with graphical representation/example species. M = transition metal, ‐X = halogenide, ‐L = amide/aryl ligand. For extended schemes, see Figures S53 and S79.

Replacing Cp* by TMP suppresses transmetallation during Co(II)→Co(0) reduction. Treating CoCl_2_ with GaTMP in the presence of Mg and H_2_ (1 bar) in THF over 18 h yields the new M_14_ cluster [Co_3_Ga_2_]H(GaTMP)_9_ (**1**). It was isolated as analytically pure, dark green crystals from the reaction filtrate in 33% preparative yield (Figure 2, top right).

**FIGURE 3:**
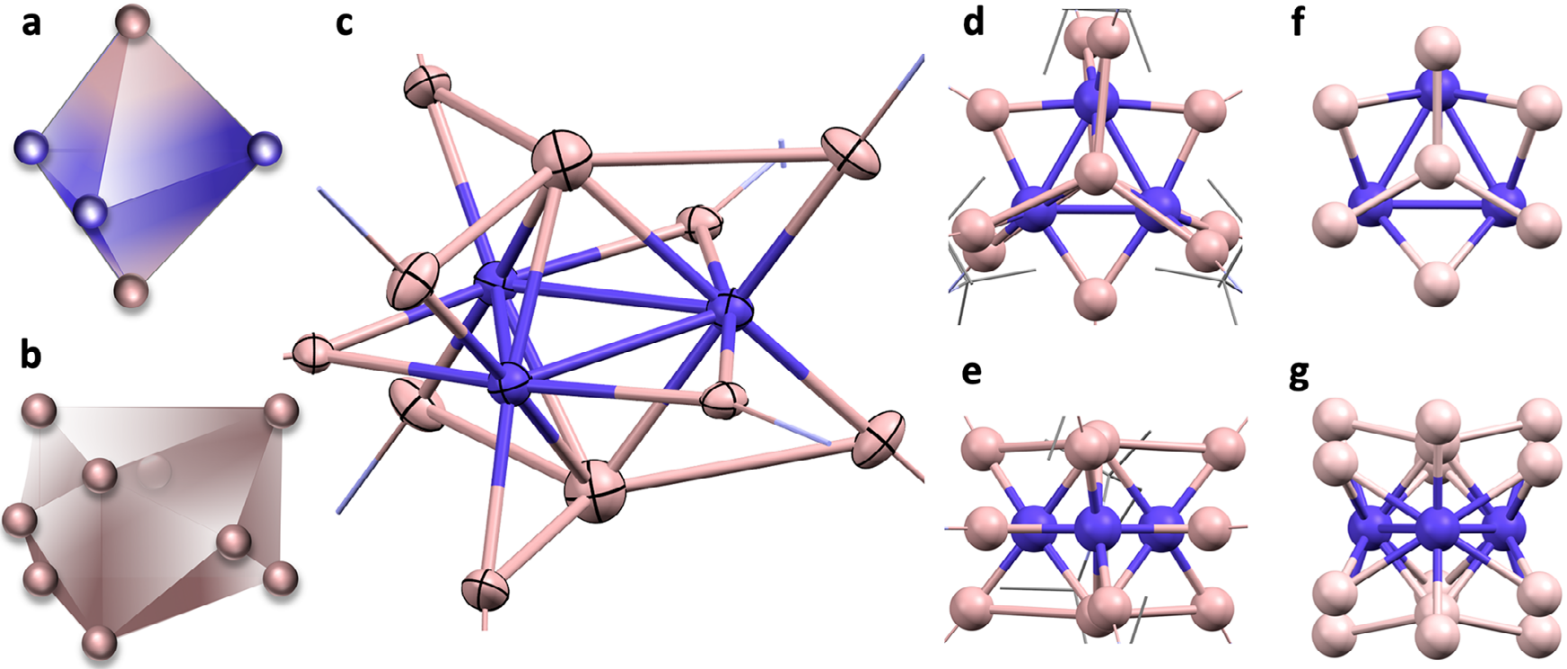
Polyhedral representations of the trigonal bipyramidal Co_2_Ga_3_ cluster kernel and the tricapped trigonal prism structure of the Ga_9_‐cluster shell (a,b). Molecular structure of **1** in the solid state (c, thermal ellipsoids given at 30% probability level). TMP omitted for clarity. Comparison of the sphere packing mode of **1** (d,e) with the central packing unit of the intermetallic Co_2_Al_5_ phase (f,g): top view (d,f), side view (e,g). Color code: cobalt: violet, gallium/aluminum: rose. Selected bond lengths/angles and a complete depiction of **1** are in the Supporting Information.

Compound **1** was rigorously characterized by SC‐XRD, NMR‐, IR‐, UV/Vis spectroscopy, and LIFDI mass spectrometry (detailed discussion of analytical data in the Supporting Information). The result of the single‐crystal structure determination (SC‐XRD) is depicted in Figure 3. The presence of exactly one hydride in **1** was confirmed by LIFDI‐MS, NMR, and IR and was assigned to a GaH moiety with a terminal hydride at one of the apical Ga atoms of the Co_3_Ga_2_ kernel.

The cluster's ^1^H‐NMR resonances and the hydride's vibrational band (1844 cm^−1^) are in accordance with the values predicted by DFT calculations. The synthesis of **1** performed under D_2_ atmosphere led to the quantitative formation of [Co_3_Ga_2_]D(GaTMP)_9_ (**1‐D**), as shown by LIFDI‐MS (*m/z* = 2208.2763, calc.: 2208.28843), ruling out solvent activation for hydride capture (D‐incorporation not observed upon using THF‐*d*_8_/H_2_ during synthesis). The assignment to a terminal GaD species was confirmed by IR (isotope shift, 1259 cm^−1^) and ^2^H‐NMR spectroscopy (*δ* = 6.60 and 6.49 ppm), in accordance with predictions by DFT. **1**’s composition and GaD ^2^H‐NMR shifts were further confirmed by quantitative formation of isotopically labeled [Co_3_^71^Ga_2_]D(^71^GaTMP)_9_ (**1‐^71^Ga‐D**), using D_2_ and ^71^GaTMP (see extended discussion in the Supporting Information).

The synthesis of **1‐D** indicates the importance of H_2_ (or D_2_) as a co‐reductant besides Mg for TMP‐ligand cleavage from Ga. This Ga‐deprotection during formation of **1** is indicated by the intermediate [Co_2_Ga](GaTMP)_8_ (identified by LIFDI‐MS, Figure S68).

Combining CoCl_2_ with GaTMP without Mg/H_2_ did not lead to the isolation of a compound with a heterometallic cluster core. Monitoring the reaction by LIFDI‐MS suggested the formation of the homoleptic, 19 valence electron complex [Co(GaTMP)_5_] ([M]^+^ *m/z* = 1108.2765, calc.: 1108.2796, Figure S62), where GaTMP is assumed to act as reduction agent and metallo‐ligand for the in situ formed Co(0) species (Figure 2, bottom left) [17, 18, 29]. Fragmentation analysis via LIFDI‐MS reveals [Co(GaTMP)_5_]^+^ to be the molecular ion [M]^+^ and no fragment ion of a larger cluster (Figures S69–S74). Unfortunately, we failed to isolate and fully characterize the compound in pure form.

By the addition of H_2_ to CoCl_2_/GaTMP to selectively cleave TMP as TMPH via hydrogenolysis in addition to the intrinsic Co/Ga redox chemistry, again [Co(GaTMP)_5_] was formed as deduced from in situ LIFDI‐MS (Figures S66, and 2, bottom right). However, when using Mg without the addition of H_2_, the selective formation of [Co_2_(GaTMP)_8_] (**3**) occurred, which was isolated in pure form (30% preparative yield) and fully characterized (Figure 2, top left, discussion of analytical data in the Supporting Information). **3** is structurally analogous to [Co_2_(CO)_8_] [8, 31], and related Co/Ga complexes [32, 33, 34, 35]. With the above‐mentioned series of [M(GaTMP)_n_], it highlights the isolobal relationship of GaTMP toward CO.

Interestingly, the *Hauser*‐base complex [Mg(TMP)(THF)μ^2^‐Cl]_2_ was found as a side‐product during this synthesis of **3**. This by‐product is literature‐known, and we confirmed its formation by ^1^H‐NMR spectroscopy and SC‐XRD (Table S5 and Figure S34) [36]. When applied individually, Mg and H_2_ are ineffective for the formation of alloyed cluster kernel structural motifs. Their combination, obviously, enables cooperative Ga‐TMP bond hydrogenolysis and TMP‐trapping at Mg (at least as an intermediate species) and thus leads to the incorporation of TMP‐free Ga atoms into the Co_2_Ga_3_ kernel of **1**. This is showcased by the transient cluster [Co_2_Ga](GaTMP)_8_, featuring a deprotected gallium atom (Figure 2, top right). [Co_2_Ga](GaTMP)_8_ was characterized by LIFDI‐MS, and [[Co_2_Ga](GaTMP)_8_]^+^ was found to be the molecular ion, following fragmentation analysis (Figures S69–S74). The described treatments (Figure 2) were performed without CoCl_2_, showing that GaTMP alone shows no reaction with Mg or/and H_2_ (Figures S41–S43), that is, it is intrinsically stable against these agents. This suggests a more complex cooperative reaction mechanism involving possible Co/Ga intermediates, which escaped our analytical monitoring so far. Note, the *Hauser*‐base complex could not be isolated as a by‐product of the synthesis of **1**, since it is not stable under H_2_ atmosphere and decomposes: Treating the *Hauser*‐base with H_2_, TMPH, and THF could be identified as decomposition products besides MgCl_2_.

The transfer of the synthetic concept toward other transition metals is a topic of our current investigation and will be reported in the future. For example, with iron, we were able to reproduce the general reaction patterns as observed for cobalt. Examples of the formed species are also depicted in Figure 2. For more detailed information, see Figure S79.

Some of the obtained compounds still feature chloride as a ligand or counter‐ion. Again, only upon combining Mg and H_2_ to deprotect the gallium and capture the chloride and the amide ligands, kernel‐alloyed species were formed, for example [Fe_2_Ga](GaTMP)_8_Cl. Replacing FeCl_2_ with the alkyl‐ and arylamide‐ligated precursors FeTMP_2_ and FeMes_2_ (Mes = 2,4,6‐trimethylphenyl) leads to comparable results: Without any additive, the mononuclear [Fe(GaTMP)_5_] was observed, whereas with Mg, the dinuclear [Fe_2_(GaTMP)_6_] and with both agents, the chloride‐free M_11_ cluster [Fe_2_Ga](GaTMP)_9_ was obtained.

Among these species, we were able to synthesize and further characterize the dinuclear complex [Fe_2_(GaTMP)_9_] (**4**). **4** was achieved by the synthesis of FeCl_2_ and GaTMP in the presence of Mg/H_2_, showing the applicability of the concept beyond Co/Ga clusters. **4** was characterized via SC‐XRD, NMR and LIFDI‐MS (see Supporting Information).

Single crystals of **1** were obtained by cooling the reaction filtrate to −30°C overnight. **1** crystallizes in the triclinic space group P‐1 (metrical data in Table S3). Its molecular structure can be rationalized as a trigonal bipyramidal heterobimetallic Co_3_Ga_2_ cluster kernel (Figure 3a), embedded into an all‐GaTMP shell in the form of a 4,4,4‐tricapped trigonal prism, resulting in a nine‐vertex deltahedron (Figure 3b).

The trigonal bipyramidal Co_3_Ga_2_ kernel consists of a Co_3_ triangle with Co–Co distances of 2.669(3)–2.673(2) Å, capped by two TMP‐free gallium atoms with Ga–Co distances of 2.382(3)–2.389(2) Å and a Ga–Ga distance of 3.640(4) Å. The nine edges of this kernel structure are coordinated by three equatorial Co_2_‐edge bridging μ^2^‐GaTMP and by six CoGa‐edge bridging μ^2^‐GaTMP moieties. The Ga‐centers of the latter are in plane with the apical core‐gallium atoms each (Figure 3c). The heterometallic CoGa‐edge bridging Ga distance toward the centroid of the particular bridged edge is longer (av. 2.286 Å) than the Co_2_‐edge bridging Ga‐distance (av. 1.964 Å). The apical Ga atoms, resting in the center of gallium triangles, are separated from their nearest three GaTMP neighbors by about 2.925 Å (av.), a value that is substantially larger than what is generally considered as a single bond distance (∼ 2.5–2.6 Å) [37], but substantially lower than twice the van der Waal radius of Ga (1.87 Å) [38]. This indicates the presence of a weak attractive Ga···Ga interaction, which will be analyzed in more detail below.

In the introduction, we highlighted structural relations between intermetallic clusters and related solid‐state intermetallic phases. Relevant examples are Co/Al phases, with Co_2_Al_5_ and the family of Co_4_Al_13_ phases being the most common [39, 40, 41]. These phases are electronically and structurally related to each other [42, 43, 44]. However, the homologous compounds Co_2_Ga_5_ or Co_4_Ga_13_ are not known in the Co/Ga phase diagram [45, 46]. The metal atom packing motif of the molecular structure of **1** shows a striking similarity to the distinct repeating building units of the intermetallic Co_2_Al_5_ phase: The Co_3_Ga_2_ trigonal bipyramidal kernel of **1** is a structural analogue of the central cluster unit of the Co_2_Al_5_ phase (Figure 3d–g) [39].

Additionally, the nine Ga positions around the cluster kernel also mimic the Al positions of the solid‐state counterpart: The two Ga_3_ triangular motifs of the (GaTMP)_9_ polyhedron can be found as a related structural repeating unit of the Co_2_Al_5_ phase (Figure 3e,f).

The remaining three Co_2_‐edge bridging μ^2^‐Ga sites can also be associated with the respective Al‐positions in the Co_2_Al_5_ phase (Figure 3e,g). In Co_2_Al_5_, these Co_2_‐edge bridging positions are occupied by one further Al atom each. **1** is sterically too crowded by the nine TMP ligands to accommodate further ligands. All in all, the structure of the M_14_ Co/Ga cluster **1** can be viewed as a molecular cutout of its closest solid‐state intermetallic counterpart Co_2_Al_5_.

The bonding situation of the title cluster **1** has been investigated through DFT calculations at BP86‐D3/TZ2P level (for details see Supporting Information). Its most stable geometry was found to have the hydride terminally bonded to one of the apical Ga positions (Ga‐H = 1.57 Å). Its metrical data are in good agreement with the x‐ray structure (Table S6). Neglecting in a first step the long Ga···Ga contacts, it is a priori possible to consider **1** as a regular 48‐electron triangular M_3_L_n_ species with three Co─Co bonds and L = GaTMP (Figure S113a) [47]. Our calculations, however, indicate that the bonding situation is closer to the limit view of a delocalized system with 15 bonding electron pairs for 18 Co–Ga contacts and no Co─Co bond (Figure S113a).

**FIGURE 4:**
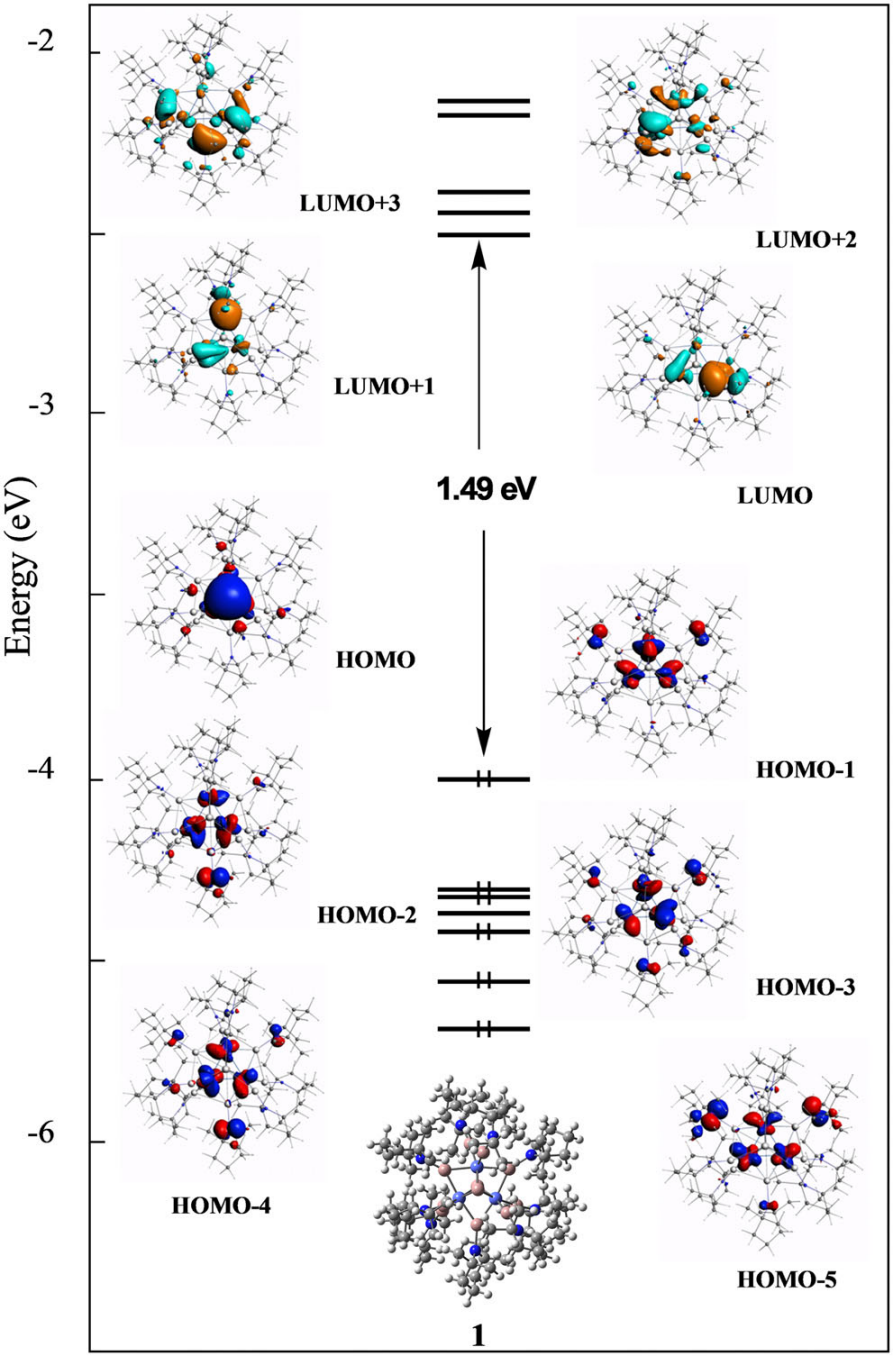
The Kohn–Sham orbital diagram of **1**. The view is perpendicular to the Ga…GaH axis, with the bare Ga atom in front.

We now turn to the analysis of the Ga_bare_…GaTMP (2.70 Å) and HGa…GaTMP (3.11 Å) interactions, which were discarded in the above description. The latter are fairly long (Table S6) and not associated with any QTAIM (quantum theory of atoms in molecules) bond critical point (bcp), and their Wiberg bond indices (WBIs) of 0.208 are more than half that of their Ga_bare_…GaTMP counterparts (0.440) [48]. For comparison, the Ga–Ga WBI computed at the same level of theory for [H_3_Ga‐GaH_3_]^2−^ [37] is 0.944. One can therefore consider these HGa…GaTMP contacts as mainly resulting from dispersion (van der Waals) forces. The shorter Ga_bare_…GaTMP contacts are associated with bcps whose indicators (Table S8) denote weak covalency. Analysis of the Kohn–Sham orbitals (Figure 4) allows tracing these interactions mainly from donation of the GaTMP ligand lone pairs into the vacant 4p(Ga_bare_) orbitals. Thus, the out‐of‐plane GaTMP ligands not only donate their lone pairs to the Co metals, but also (to some extent) to the bare Ga of the Co_3_Ga_2_ kernel, thus contributing to increase the delocalization over the whole Co_3_Ga_11_ core. This substantiates the conceptual deconstruction of **1** into an alkylamide‐ligated Co_3_Ga_11_ core, with the alloyed Co_3_Ga_2_ kernel.

## 3 Conclusion

The herein presented synthetic protocol toward the synthesis of heterobimetallic clusters enabled access to compounds with alloyed cluster kernels as molecular models of their compositionally related intermetallic *Hume–Rothery* type solid‐state phases. First results indicate generalization of the Mg/H_2_ cooperative reduction and alkylamide deprotection concept for other metal combinations, for example, Fe/Ga, to find systematic access to truly M/E alloyed clusters, that may be suitable as atom‐precise molecular models of solid‐state intermetallics. This is relevant not only from a structural and bonding point of view, but also opens an avenue to mimic specific (catalytic) reactivities of bimetallic solid‐state alloys with ligated clusters.

It should be noted that cluster **1** turned out to be inactive in H_2_ activation and alkyne semihydrogenation, compared to the initially mentioned Ni/Ga cluster [8].

Our current efforts are targeted to access systems showing the mentioned reactivities by the synthesis strategies presented in this work. We intend to investigate new types of (even larger) TMP‐bearing heterobimetallic clusters with ligand‐free cluster sites accessible for reactivities toward small molecules.

## Conflicts of Interest

The authors declare no conflicts of interest.

## Supporting information




**Supporting File 1**: anie71710‐sup‐0001‐SuppMat.pdf.

**Supporting File 2**: anie71710‐sup‐0002‐Data.zip.

## Data Availability

The data that support the findings of this study are available from the corresponding author upon reasonable request.
