# Supplementary material for: Molecular Mimics of Intermetallic Phases: Selective Alkylamide‐Ligand Deprotection Drives Co/Ga Cluster Formation
Source: Angew Chem Int Ed Engl. 2026 Mar 13;65(19):e23687. doi: 10.1002/anie.202523687 (PMC13134592; doi:10.1002/anie.202523687)
Supplement: Supplementary file 1 — Supporting File 1: anie71710‐sup‐0001‐SuppMat.pdf. [file ANIE-65-e23687-s002.pdf]

Supporting Information

Molecular Mimics of Intermetallic Phases: Selective Alkylamide-Ligand Deprotection Drives Co/Ga Cluster Formation

Fabrizio E. Napoli,[a,b] Raphael Bühler,[a,b] Johannes Stephan,[a,b] Samia Kahlal,[c] Jean-Yves Saillard,[c] Christian Gemel,[a,b] Roland A. Fischer[a,b]*

a-Technical University of Munich, TUM School of Natural Sciences, Department of Chemistry, Chair of Inorganic and Metal-Organic Chemistry, Lichtenbergstraße 4, D-85748 Garching, Germany. b-Catalysis Research Centre, Technical University Munich, Ernst-Otto-Fischer Strasse 1, D-85748 Garching, Germany. c-Univ Rennes, CNRS, ISCR-UMR 6226, F-35000 Rennes, France.

The manuscript was written through contributions of all authors. All authors have given approval to the final version of the manuscript

Abstract: The heterobimetallic M_{14} cluster $[Co_3Ga_2]H(\mu^2\text{-GaTMP})_9$ (**1**, TMP = 2,2,6,6-tetramethylpiperidinyl) was obtained by treating $CoCl_2$ with GaTMP in presence of Mg and H_2 as reduction additives. **1** features a trigonal bipyramidal Co_3Ga_2 kernel with one substituent-free Ga atom and one exposed GaH moiety inside a metallo-environment of nine edge-bridging GaTMP ligands. The resulting Co_3Ga_{11} cluster core mimics a packing motif of the structurally related intermetallic phase Co_2Al_5. The GaTMP ligands not only donate their lone pairs to the Co, but also to the exposed, bare Ga of the Co_3Ga_2 kernel, thus contributing to increase the delocalization over the M_{14} core, according to bonding analysis. The synthesis of **1** represents proof-of-concept to steer the coordination of GaTMP to transition metal centers towards a kernel-alloyed Co/Ga cluster formation. This is rationalized by Mg/H_2 induced Ga deprotection and TMP-trapping in the *Hauser*-base complex $[Mg(TMP)(THF)\mu^2Cl]_2$. The method bears potential for generalization and first results were obtained for related Fe/Ga clusters.

Table of Contents

Experimental Methodology…………2

General Aspects…………2

Instrumentation…………2

Crystallography…………3

Fragmentation Analysis…………4

Computational Details…………5

Synthetic Procedures…………6

Crystallographic Data…………12

Extended Discussion of Analytical Data…………17

$[Ga_{10}]H(CoCp^*)_6$ (**2**)…………17

$[Co_3Ga_2]H(\mu^2\text{-}GaTMP)_9$ (**1**)…………19

$[Co_2(GaTMP)_8]$ (**3**)…………22

$[Fe_2(GaTMP)_9]$ (**4**)…………23

NMR Spectra…………25

IR Spectra…………45

UV-Vis Spectra…………48

LIFDI Mass Spectra…………49

Ga/CoCp* Library…………49

Co/GaTMP Clusters and Complexes…………51

Co/GaTMP Fragmentation Analysis…………59

Fe/GaTMP Clusters and Complexes…………62

Extended Bonding Analysis of **1** from DFT Calculations…………82

References…………86

Experimental Methodology

General Aspects

All manipulations were carried out in standardized Schlenk flasks/tubes or in the glovebox under inert argon atmospheres. Heating baths were filled with paraffin oil and the temperature was controlled and regulated by means of contact thermometers. All used glassware was heated with hexamethyldisilazane prior to use to achieve passivated glass surfaces.

The solvents for moisture-sensitive reactions were processed by a solvent purification system from M. Braun GmbH, model MB-SPS-800, and stored over molecular sieve (3 Å). Final H_2O contents of all solvents were checked by Karl Fischer titration and were below 5 ppm before using. All used chemicals were purchased from commercial suppliers and were used without further purification unless otherwise specified. GaCp*[1] and GaTMP[2] were synthesized according to literature-known procedures.

Instrumentation

The NMR spectra were recorded at 298 K either on a *Bruker* Advance III AV-400 US, a *Bruker* AV-II-500 equipped with a cryo probehead for improved ^{13}C NMR spectra or a *Bruker* Ascend AVHD-400 for ^{2}H NMR spectra. The solvents used were benzene-d_6 and toluene-d_8. Both the used solvents were degassed prior to used and were stored over molecular sieve (3 Å). Spectra were referenced to the solvent's residual proton signal.

The NMR spectra were evaluated using the MestreNova 12.0 program from Mestrelab Research. The chemical shifts δ are given in ppm (parts per million). For the signals, the multiplicity (s = singlet, d = doublet, t = triplet or m = multiplet), the coupling constant *J* in Hertz Hz and the number of nuclei determined by integration are given in brackets.

The FT-IR spectra were taken on an ALPHA-T FT-IR spectrometer from Bruker with a platinum ATR unit operated in a glovebox under argon atmosphere with 48 scans per measurement and a resolution of 4 cm^{-1}. The spectra were evaluated using the software OPUS.

UV/Vis spectra were recorded on a Cary 60 spectrometer from Agilent Technologies. The applied scan rate was 600 nm/min. For the measurements, a 10.00 mm quartz glass cuvette from Hellma Analytics was used, which was modified into a Schlenk-cuvette. Baseline correction was used prior to the measurement using the respective solvent (hexane).

The mass spectra were taken using a Linden CMS LIFDI as ionization source and a ThermoFisher Scientific Exactive Plus Orbitrap as detector. The sample application was performed *via* a fumed silica capillary from a glovebox under an argon atmosphere to enable the measurement of highly air-sensitive compounds.[3] Evaluation of obtained mass spectra were performed using the program FreeStyle 1.3, provided by ThermoFisher Scientific. Prediction of theoretical mass spectrometric patterns were obtained by the enviPat Web tool by eawag aquatic research.[4]

Crystallography

Data were collected on a single crystal x-ray diffractometer equipped with a CMOS detector (Bruker APEX IV, κ-CMOS) and a TXS rotating anode with MoK_α radiation (λ = 0.71073 Å) and a Helios optic or equipped with a CPAD detector (Bruker Photon II) for compounds **1**, **4** and $[Mg(TMP)(THF)\mu^2\text{-}Cl]_2$. For compound **3**, a single crystal x-ray diffractometer equipped with an IMS microsource with MoK_α radiation (λ = 0.71073 Å) and a Helios optic for compound **3** was used. Data for **1**, **3, 4** and $[Mg(TMP)(THF)\mu^2\text{-}Cl]_2$ were processed using the APEX4 software package.[C1] For **2**, x-ray intensity data were collected on a Xcalibur2 four-circle diffractometer with a fine-focus sealed X-ray tube with Mo K_α radiation (λ=0.71073 Å) using CrysAlisPro.[C2] The diffractometer was equipped with a graphite monochromator and a Sapphire2 large Be window detector. Measurements were performed on single crystals coated with perfluorinated ether. The crystals were fixed on top of a Kapton micro sampler and frozen under a stream of cold nitrogen. A matrix scan was used to determine the initial lattice parameters. Reflections were corrected for Lorentz and polarization effects, scan speed, and background using SAINT.[C3] Absorption correction, including odd and even ordered spherical harmonics was performed using SADABS.[C4] Data for **2** were corrected for absorption effects including odd and even ordered spherical harmonics by the analytical method (SCALE3 ABSPACK).[C2] Space group assignments were based upon systematic absences, E statistics, and successful refinement of the structures. The structures were solved using SHELXT with the aid of successive difference Fourier maps, and were refined against all data using SHELXL-2019/1 in conjunction with SHELXLE.[C5,C6,C7] Hydrogen atoms were calculated in ideal positions as follows: Methyl hydrogen atoms were refined as part of rigid rotating groups, with a C–H distance of 0.98 Å and $U_{iso(H)} = 1.5 \cdot U_{eq(C)}$. Other H atoms were placed in calculated positions and refined using a riding model, with methylene and aromatic C–H distances of 0.99 Å and 0.95 Å, respectively, other C–H distances of 1.00 Å, all with $U_{iso(H)} = 1.2 \cdot U_{eq(C)}$. Non-hydrogen atoms were refined with anisotropic displacement parameters. Full-matrix least-squares refinements were carried out by minimizing $\Sigma\, w(F_o^2 - F_c^2)^2$ with the SHELXL weighting scheme.[C6] Neutral atom scattering factors for all atoms and anomalous dispersion corrections for the non-hydrogen atoms were taken from *International Tables for Crystallography*.[C8] A split layer refinement was used for disordered groups and additional restraints on distances, angles and anisotropic displacement

parameters were employed to ensure convergence within chemically reasonable limits, if necessary. Whole molecule disorder and rotational disorder, e.g. of amide moieties or toluene solvent molecules, was modelled using the *DSR* tool plugin within SHELXLE.[C9] For compound **1**, two heavily disordered molecules of *n*-hexane as well as one molecule of *n*-pentane for compound **4** were treated as a diffuse contribution to the overall scattering without specific atom positions using the PLATON/SQUEEZE procedure.[C10] Images of the crystal structures were generated with Mercury.[C11]

Fragmentation Analysis

The analysis of the fragmentation behavior was assessed for the complexes [Co(GaTMP)$_5$] and [Co$_2$Ga](GaTMP)$_8$. The following procedure is originally based on the energy-dependent ESI-MS and stepwise collision energy for mass spectrometers equipped with an orbitrap.[5-6] This procedure was initially used for fragmentation analysis of peptides. The here described fragmentation analysis was already applied for fragmentation analysis of living libraries of molecular clusters on an orbitrap-equipped mass spectrometer coupled to a LIFDI ionization source and is also based on that procedure.[3, 7]

Mass spectra were recorded at a varied normalized collision energy between values of 10 and 100 (arbitrary units), with a normalized collision energy of 10 being the lowest collision energy possible on the used mass spectrometric setup (*vide supra*). The pattern of every detected ion associated to the molecules of interest were integrated, normalized and compared towards the respective integral of the ion at the lowest relative collision energy of 10. The intensity of every pattern of interest was determined by a computerized integration of the pattern in the mass spectrum.

An increasing peak intensity for increasing collision energy of an observed ion of interest is associated with fragment ions. This is caused by the enhanced occurrence of fragmentation events at higher collision energies, and therefore results in an increase of peak intensity of fragment ions. The opposite is the case for molecular ions: A decreasing peak intensity for increasing collision energy is associated with molecular ions, due to the same reason as described above. Nevertheless, sometimes no clear decision can be made if the intensities of the observed ions show a fluxional or continuous behavior over increasing relative collision energy.

For collision energies over 100, a decrease of all ions associated to the investigated compounds – fragment ions and molecular ions – was observed. This behavior is caused by an increased fragmentation to even smaller fragments.

The applied heating rate used for all of the here described measurements was 60 mA/s with a maximum applied current of 120 mA reached after a total measurement time of two minutes.

Beside the variation of the relative collision energy, there are further factors that influence the observed peak intensity and cause statistic fluctuations during a set of measurements.

One factor is the preparation of the analyte on the LIFDI-emitter before the measurement, since the final amount of the analyte on the emitter cannot be fully controlled. To reduce the influence of this factor, a constant injection time of the analyte solution onto the emitter was ensured. Beside the analyte preparation, also the ionization process influences intensity fluctuations by alteration of the emitter through decomposition of the analyte and decomposition of metal on the emitter, as well as geometric alterations or rearrangements on the surface of the emitter. Those factors also cause a constant, but slow decrease of sensitivity of the emitter for mass spectrometric measurements.

To keep the influence of the emitter on the measurement as low as possible, a complete fragmentation analysis is always conducted on the same emitter.

The obtained integrated and normalized peak intensities of a molecular or fragment ion of interest against increasing collision energy were analyzed by linear regression analysis to assign each ion to either molecular or fragment ion. A linear regression with a negative slope corresponds to a decrease in intensity against increasing collision energy and therefore to a molecular ion, and *vice versa* (*vide supra*). Besides that, two further plots were shown to illustrate the behavior of molecular and fragment ions over time: The relative ratio of the identified molecular ion and the accumulated related fragment ions over increasing collision energy and a stack of representative mass spectra at different collision energies to visualize the change in intensity of the different types of ions as well an allocation of sum formulas to the identified molecular and related fragment ions.

Computational Details

Density Functional Theory (DFT) calculations were carried out with the use of the Amsterdam Density Functional code (ADF2020.101)[A] incorporating scalar relativistic corrections *via* the ZORA Hamiltonian.[B,C] The BP86 functional[X,Y] was used, with the addition of Grimme's D3(BJ) empirical corrections[D] in order to take into account dispersion effects. All the geometry optimizations were performed with the all-electron triple-ξ Slater basis set plus two polarization functions (STO-TZ2P).[E] Natural atomic orbital (NAO) populations and Wiberg bond indices were computed with the natural bond orbital NBO6.0 program[F] implemented in the ADF2020.101 package. Quantum theory of atoms in molecules (QTAIM)[G,H,I] data were computed at the same level of theory with the ADF2020.101 package. The NMR chemical shifts were computed according to the gauge-independent atomic orbitals (GIAO) method.[J,K]

Synthetic Procedures

Synthesis of $[Ga_xCo_y](CoCp^*)_z$ (including $[Ga_{10}]H(CoCp^*)_6$ (2) as part of the ensemble)

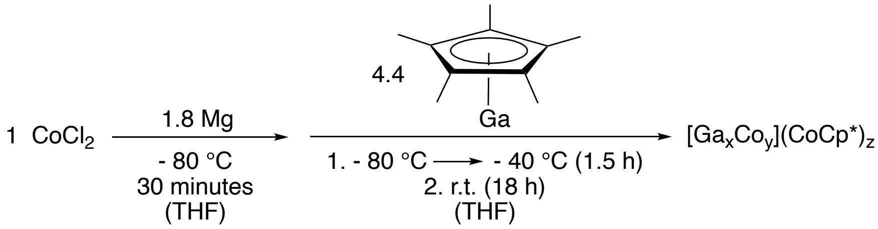

58.0 mg of $CoCl_2$ (0.45 mmol, 1.0 eq.) and 20.0 mg Mg powder (0.81 mmol, 1.8 eq.) were added to 10 mL of dried THF. The suspension was cooled to -80 °C and stirred for 30 minutes. Subsequently, a suspension of 410 mg GaCp* (2.00 mmol, 4.4 eq.) in 5 mL THF at -80 °C was added to the $CoCl_2$/Mg suspension. The reaction mixture was allowed to warm to -40 °C over a course of 1.5 h, removed from the cooling bath, and stirred for subsequent 18 h at room temperature. Afterwards, the solvent was removed *in vacuo* and extracted upon addition of 20 mL of dried hexane and separated using Whatman filtration. The resulting dark solution was reduced under lowered pressure and stored at -30 °C over night. The resulting solids of the ensemble formed over the course of a few days. ^{13}C NMR shifts were obtained by ^{13}C and 2D NMR spectroscopy. $[Ga_{10}]H(CoCp^*)_6$ (**2**) was obtained by crystal picking. A yield could not be determined.

^{1}H NMR (400 MHz, 298 K, benzene-d_6): δ [ppm] = 2.64 (s), 2.62 (s), 2.52 (s), 2.41 (s), 2.36-2.34 (m), 2.32 (s), 2.32 (s), 2.30 (s), 2.28 (s), 2.26 (s), 2.22-2.20 (m), 2.17 (s), 2.16 (s), 2.14 (s), 2.13 (s), 2.10 (s), 2.09 (s), 2.06 (s), 2.03 (s), 2.00 (m), 1.93 (s), 1.87 (s), 1.80 (s), 1.79 (s), 1.77 (s), 1.75 (s), 1.71 (s), 1.70 (s), 1.67 (s), 1.64 (s), 1.58 (s), 1.51 (s), 1.44 (s), 1.41 (s), 1.36 (s), 1.34 (s), 1.16 (s), 1.10 (s). **^{13}C NMR** (101 MHz, 298 K, benzene-d_6) δ [ppm] = 113.54, 109.01, 108.58, 89.69, 87.54, 87.11, 86.68, 39.10, 31.97, 29.32, 23.06, 19.77, 14.37, 13.41, 13.25, 12.82, 12.39, 11.73, 11.26, 11.15, 10.67, 9.91, 9.38.). **IR**: υ [cm^{-1}] = 2961, 2890, 2849, 2713, 2273, 1784, 1649, 1560, 1474, 1449, 1425, 1374, 1258, 1154, 1089, 1065, 1022, 868, 792, 693, 659, 630, 606, 589, 544, 494. **LIFDI-MS**: *m/z* [a.u.] = 2183.4588 (calc.: 2183.46645) $[[Ga_{11}Co](CoCp^*)_7]^+$, 2124.5365 (calc.: 2124.53325) $[[Ga_{11}](CoCp^*)_7]^+$, 1990.4337 (calc.: 1990.40724) $[[Ga_{11}Co](CoCp^*)_6\ \text{-H}]^+$, 1919.4910 (calc.: 1919.48227) $[[Ga_{10}Co](CoCp^*)_6\ \text{-H}]^+$, 1862.5625 (calc.: 1862.56473) $[[Ga_{10}]H(CoCp^*)_6]^+$, 1847.5581 (calc.: 1847.55224) $[[Ga_9Co](CoCp^*)_6\ \text{-2H}]^+$, 1792.6244 (calc.: 1792.63151) $[[Ga_9](CoCp^*)_6]^+$, 1723.7325 (calc.: 1723.72214) $[[Ga_8]H_2(CoCp^*)_6]^+$, 1709.7002 (calc.: 1709.69865) $[[Ga_7Co](CoCp^*)_6\ \text{-2H}]^+$, 1653.7822 (calc.: 1653.78893) $[[Ga_7]H(CoCp^*)_6]^+$, 1582.8558 (calc.: 1582.86383) $[[Ga_6]H(CoCp^*)_6]^+$, 1528.6799 (calc.: 1528.66343) $[[Ga_8]H(CoCp^*)_5]^+$, 1513.9350 (calc.: 1513.93846) $[[Ga_5]H(CoCp^*)_6]^+$.

Table S1 List of selected cluster species identified in the LIFDI mass spectrum of the cluster library of the form $[Ga_xCo_y](CoCp^*)_z$ (x = 5-11, y = 0,1 and z = 6,7).

Sum formulae of species	*m/z*	Theoretically predicted *m/z*
$[\mathbf{[Ga_{11}Co]}(CoCp^*)_7]^+$	2183.4588	2183.46645
$[\mathbf{[Ga_{11}]}(CoCp^*)_7]^+$	2124.5365	2124.53325
$[\mathbf{[Ga_{10}Co]}(CoCp^*)_6\ \text{-H}]^+$	1919.4910	1919.48227
$[\mathbf{[Ga_{10}]}H(CoCp^*)_6]^+$ (**2**)	1862.5625	1862.56473
$[\mathbf{[Ga_9Co]}(CoCp^*)_6\ \text{-2H}]^+$	1847.5581	1847.55224
$[\mathbf{[Ga_9]}(CoCp^*)_6]^+$	1792.6244	1792.63151
$[\mathbf{[Ga_8]}H_2(CoCp^*)_6]^+$	1723.7325	1723.72214
$[\mathbf{[Ga_7Co]}(CoCp^*)_6\ \text{-2H}]^+$	1709.7002	1709.69865
$[\mathbf{[Ga_7]}H(CoCp^*)_6]^+$	1653.7822	1653.78893
$[\mathbf{[Ga_6]}H(CoCp^*)_6]^+$	1582.8558	1582.86383

Synthesis of Co/GaTMP complexes and clusters

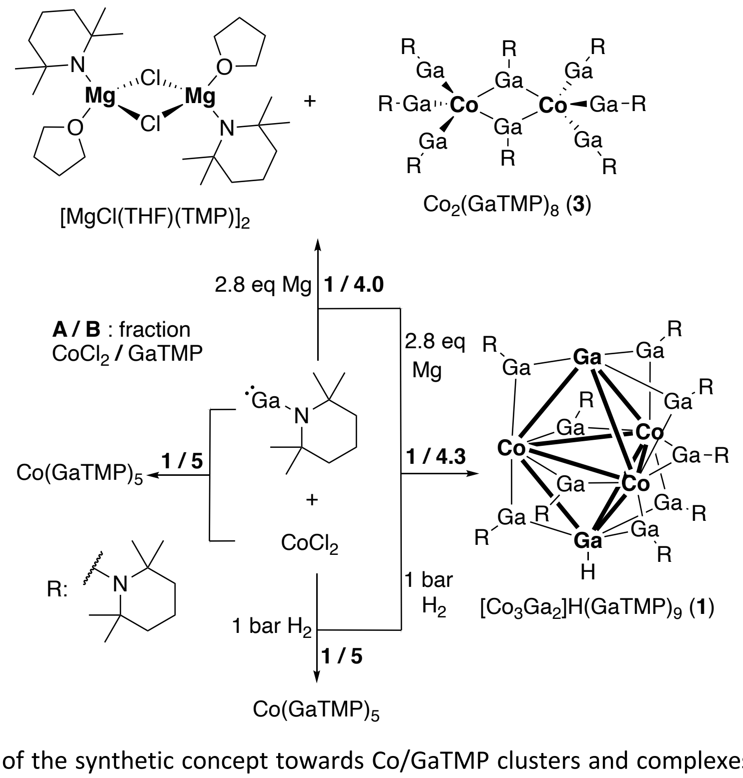

Scheme S1 Overview of the synthetic concept towards Co/GaTMP clusters and complexes, starting from $CoCl_2$ and GaTMP, upon addition of Mg and H_2 as reducing agents.

Synthesis of $[Co_3Ga_2]H(GaTMP)_9$ (1) and $[Co_3{}^{71}Ga_2]D({}^{71}GaTMP)_9$ (1-^{71}Ga-D)

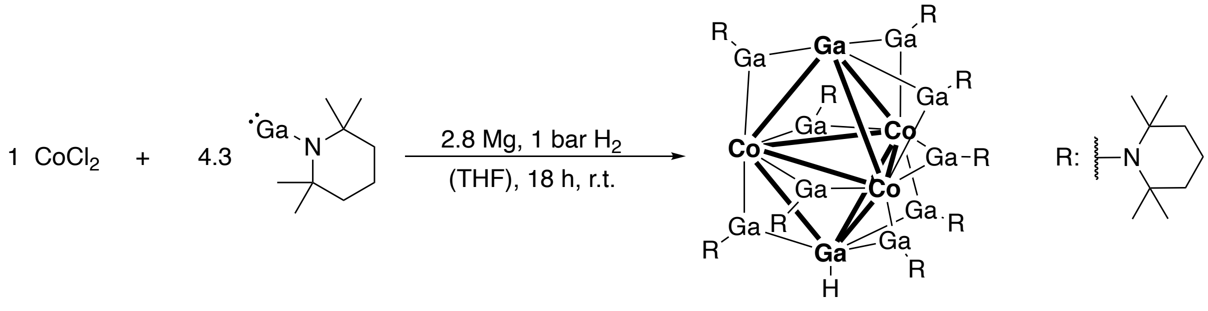

$[Co_3Ga_2]H(GaTMP)_9$ (**1**)

Synthesis of $[Co_3Ga_2]H(GaTMP)_9$ (**1**)

4.14 mg $CoCl_2$ (0.0319 mmol, 1.0 eq.), 2.14 mg Mg powder (0.0880 mmol, 2.8 eq.) and 28.57 mg GaTMP (0.137 mmol, 4.3 eq.) were added to a J-Young NMR tube. Subsequently, 1 mL of dried THF was added and the tube was immediately cooled in liquid nitrogen. The J-Young tube was degassed once and pressurized with 1 bar of H_2. This procedure was performed nine times in total. The reaction mixtures were allowed to rest for 18 h at room temperature and were collected into one Schlenk-flask afterwards. The solvent was removed *in vacuo* and extracted upon addition of 30 mL of dried hexane and separated using Whatman filtration. The resulting dark solution was concentrated under reduced pressure and stored over night at -30 °C. The resulting crystals were separated using Whatman filtration and dried *in vacuo*, leading to **1** as dark green crystals (70.2 mg, 0.0317 mmol, 33%). **1** was stored at -30°C. **^{1}H-NMR** (400 MHz, 298 K, toluene-d_8): δ [ppm] = 1.97 (36 H, s, methyl, μ^2-GaTMP), 1.84 (36 H, s, methyl, [GaH]-μ^3-GaTMP), 1.71 (36 H, s, methyl, [Ga]-μ^3-GaTMP), 1.65 (108 H, m, γ, $\mu^{2/3}$-GaTMP), 1.45 (72 H, t, β, μ^2-GaTMP and [Ga]-μ^3-GaTMP), 1.42 ppm (36 H, t, β, [GaH]-μ^3-GaTMP). **^{13}C-NMR** (101 MHz, 298 K, toluene-d_8): δ [ppm] = 57.33 (α, [Ga]-μ^3-GaTMP), 56.05 (α, [GaH]-μ^3-GaTMP), 55.35 (α, μ^2-GaTMP), 40.73 (β, [Ga]-μ^3-GaTMP), 40.30 (β, μ^2-GaTMP), 39.44 (β, [GaH]-μ^3-GaTMP), 37.03 (methyl, [Ga]-μ^3-GaTMP), 36.74 (methyl, μ^2-GaTMP), 35.60 (methyl, [GaH]-μ^3-GaTMP), 18.98 (γ, $\mu^{2/3}$-GaTMP). Alternatively: **^{13}C-NMR** (101 MHz, 298 K, benzene-d_6): δ [ppm] = 57.34 (s, α, [Ga]-μ^3-GaTMP), 56.07 (s, α, [GaH]-μ^3-GaTMP), 55.36 (s, α, μ^2-GaTMP), 41.17 (s, β, [Ga]-μ^3-GaTMP), 40.48 (s, β, μ^2-GaTMP), 39.86 (s, β, [GaH]-μ^3-GaTMP), 37.31 (s, methyl, [Ga]-μ^3-GaTMP), 37.05 (s, methyl, μ^2-GaTMP), 35.64 (s, methyl, [GaH]-μ^3-GaTMP), 18.85 (s, γ, $\mu^{2/3}$-GaTMP). **^{2}H-NMR** (61.402 MHz, 298 K, DMSO-d_6): δ [ppm] = 6.60 (0.40 D, s, ^{71}Ga*D*) 6.49 (0.60 D, s, ^{69}Ga*D*). **IR**: υ [cm^{-1}] = 2997, 2911, 2861, 2655, 1844, 1450, 1374, 1360, 1343, 1290, 1234, 1199, 1170, 1129, 1079, 1057, 1007, 966, 948, 927, 904, 865, 742, 511, 424. **LIFDI-MS**: *m/z* [a.u.] = 2207.2822 (calc.: 2207.28215) [M]$^+$, 2068.1475 (calc.: 2268.14563) [M-TMP]$^+$, 1994.1878 (calc.: 1994.19713) [M-GaTMP-2H]$^+$, 1926.9925 (calc.: 1926.99348) [M-2TMP]$^+$, 1857.0843 (calc.: 1857.07631) [MH-TMP-GaTMP]$^+$, 1783.1223 (calc.: 1783.11181) [M-2GaTMP-4H]$^+$, 1647.9901 (calc.: 1648.00665) [MH-TMP-2GaTMP]$^+$, 1572.0374 (calc.: 1572.04255) [M-3GaTMP-H]$^+$. **UV/Vis:** 296 (sh) nm, 410 (sh) nm, 604 (sh) nm.

Synthesis of $[Co_3{}^{71}Ga_2]D({}^{71}GaTMP)_9$ (**1-^{71}Ga-D**)

4.14 mg $CoCl_2$ (0.0319 mmol, 1.0 eq.), 2.14 mg Mg powder (0.0880 mmol, 2.8 eq.) and 28.97 mg ^{71}GaTMP (0.137 mmol, 4.3 eq.) were added to a J-Young NMR tube. Subsequently, 1 mL of dried THF was added and the tube was immediately cooled in liquid Nitrogen. The J-Young tube was degassed once and pressurized with 1 bar of D_2. This procedure was performed four times in total. The reaction mixtures were allowed to rest for 18 h at room temperature and were collected into one Schlenk-flask afterwards. The solvent was removed *in vacuo* and extracted upon addition of 15 mL of dried hexane and separated using Whatman filtration. The resulting dark solution was concentrated under reduced pressure and stored over night at -30 °C. The resulting crystals were separated using Whatman filtration and dried *in vacuo*, leading to **1-^{71}Ga-D** as dark green crystals (10.0 mg, 0.00450 mmol, 11%). **^{2}H-NMR** (61.402 MHz, 298 K, DMSO-d_6): δ [ppm] = 6.62 (1 D, s, ^{71}Ga*D*). **LIFDI-MS**: *m/z* [a.u.] = 2220.2809 (calc.: 2220.28138) $[M]^+$, 2080.1297 (calc.: 2080.13732) $[M\text{-}TMP]^+$, 2006.1717 (calc.: 2006.19067) $[M\text{-}GaTMP\text{-}D\text{-}H]^+$.

Synthesis of $Co_2(GaTMP)_8$ (3)

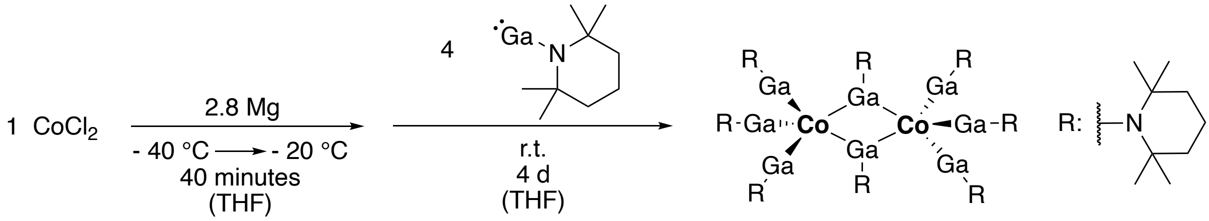

$Co_2(GaTMP)_8$ (**3**)

69.6 mg $CoCl_2$ (0.536 mmol, 1.0 eq.) and 36.0 mg Mg (1.48 mmol, 2.8 eq.) were added to 6 mL of dried THF at -40 °C. The reaction mixture was stirred and allowed to reach -20 °C over a course of 40 minutes, resulting in a light blue suspension. Afterwards, a solution of 445.9 mg of GaTMP (2.14 mmol, 4.0 eq.) in 12 mL dried THF were cooled to -20 °C and added to the reaction mixture. The suspension was allowed to reach room temperature and stirred for four days. Afterwards, the solvent was removed *in vacuo* and extracted upon addition of 40 mL of hexane and separated using Whatman filtration. The resulting dark solution was concentrated under reduced pressure and stored over night at -30 °C. The resulting dark crystals were separated using Whatman filtration and dried *in vacuo*, leading to **3** as black crystals (144 mg, 0.0804 mmol, 30%). **3** was stored at -30°C. **^{1}H-NMR** (400 MHz, 298 K, benzene-d_6): δ [ppm] = 1.75 (96 H, s, methyl), 1.50 (32 H, t, β), 1.42 (16 H, br s, γ). **^{13}C-NMR** (101 MHz, 298 K, benzene-d_6): δ [ppm] = 55.10 (s, α), 40.38 (s, β), 34.97 (methyl), 25.36 (s, γ). **IR:** υ [cm^{-1}] = 2995, 2917, 2863, 2841, 2655, 1450, 1374, 1360, 1343, 1306, 1290, 1234, 1199, 1172, 1129, 1102, 1057, 1009, 983, 964, 929, 911, 863, 804, 744, 540, 507, 426. **LIFDI-MS**: *m/z* [a.u.] = 1796.4160 (calc.: 1796.42128) $[M]^+$, 1587.3400 (calc.: 1587.35167) $[M\text{-}GaTMP]^+$, 1447.2040 (calc.: 1447.21136) $[M\text{-}GaTMP\text{-}TMP]^+$, 1376.2767 (calc.: 1376.28238) $[M\text{-}2GaTMP]^+$, 1167.2005 (calc.: 1167.21279) $[M\text{-}3GaTMP]^+$, 958.1365 (calc.: 958.14320) $[M\text{-}4GaTMP]^+$.

Synthesis of $Fe_2(GaTMP)_9$ (4)

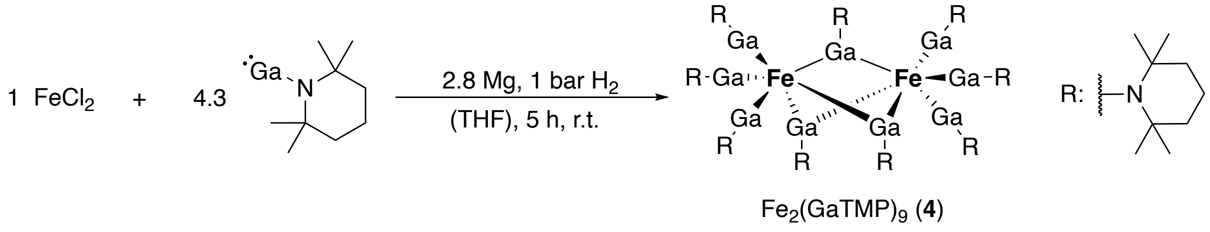

4.00 mg $FeCl_2$ (0.0316 mmol, 1.0 eq.), 2.15 mg Mg powder (0.0880 mmol, 2.8 eq.) and 28.53 mg GaTMP (0.136 mmol, 4.3 eq.) were added to a J-Young NMR tube. Subsequently, 1 mL of dried THF was added and the tube was immediately cooled in liquid Nitrogen. The J-Young tube was degassed once and pressurized with 1 bar of H_2. This procedure was performed eight times in total. The reaction mixtures were allowed to rest for 5 h at room temperature and were collected into one Schlenk-flask afterwards. The solvent was removed *in vacuo* and extracted upon addition of 30 mL of dried pentane and separated using Whatman filtration. The resulting dark solution was concentrated under reduced pressure and stored over night at -30 °C. The resulting crystals were separated using Whatman filtration and dried *in vacuo*, leading to **4** as black crystals, which were collected by crystal picking. A yield could not be determined. **^{1}H-NMR** (400 MHz, 298 K, benzene-*d*$_6$): δ [ppm] = 1.63 (18 H, br s, γ), 1.56 (108 H, s, methyl), 1.44 (36 H, t, β). **^{13}C-NMR** (101 MHz, 298 K, benzene-*d*$_6$): δ [ppm] = 53.61 (s, α), 40.38 (s, β), 35.14 (methyl), 18.40 (s, γ). **LIFDI-MS**: *m/z* [a.u.] = 2001.5098 (calc.: 2001.49470) $[M]^+$, 1790.4278 (calc.: 1790.42533) $[M\text{-}GaTMP]^+$, 1370.2768 (calc.: 1370.28642) $[M\text{-}3GaTMP]^+$.

Crystallographic data

Table S2 [Ga_{10}]H(CoCp*)$_6$

CCDC number	2496873
Empirical formula	$C_{65}H_{102}Co_6Ga_{10}$
Formula weight	1934.24
Temperature [K]	103.3(10)
Crystal system	monoclinic
Space group (number)	$P2_1/n$ (14)
a [Å]	12.6775(10)
b [Å]	14.5018(11)
c [Å]	19.3800(15)
α [°]	90
β [°]	94.394(7)
γ [°]	90
Volume [Å^3]	3552.5(5)
Z	2.00
ρ_{calc} [gcm^{-3}]	1.808
μ [mm^{-1}]	5.116
F(000)	1928
Crystal size [mm^3]	0.093×0.144×0.301
Crystal colour	black
Crystal shape	block
Radiation	Mo K_α (λ=0.71073 Å)
2θ range [°]	5.83 to 52.83 (0.80 Å)
Index ranges	$-15 \le h \le 15$<br>$-18 \le k \le 18$<br>$-24 \le l \le 24$
Reflections collected	32055
Independent reflections	7258<br>R_{int} = 0.1992<br>R_{sigma} = 0.2220
Completeness to θ = 26.3154°	99.8
Data / Restraints / Parameters	7258 / 225 / 372
Goodness-of-fit on F^2	1.041
Final *R* indexes [$I \geq 2\sigma(I)$]	R_1 = 0.0991<br>wR_2 = 0.2034
Final *R* indexes [all data]	R_1 = 0.2198<br>wR_2 = 0.2565
Largest peak/hole [eÅ^{-3}]	2.04/−1.00

Table S3 $[Co_3Ga_2]H(GaTMP)_9$ (**1**)

CCDC number	2496871
Empirical formula	$C_{81}H_{162}Co_3Ga_{11}N_9$
Formula weight	2205.90
Temperature [K]	100(2)
Crystal system	triclinic
Space group (number)	$P\bar{1}$ (2)
a [Å]	16.884(11)
b [Å]	16.891(11)
c [Å]	23.81(2)
α [°]	89.98(3)
β [°]	69.29(2)
γ [°]	60.055(11)
Volume [Å^3]	5371(8)
Z	2
ρ_{calc} [gcm^{-3}]	1.364
μ [mm^{-1}]	3.196
F(000)	2266
Crystal size [mm^3]	0.060×0.125×0.133
Crystal colour	black
Crystal shape	fragment
Radiation	MoK_α (λ=0.71073 Å)
2θ range [°]	4.18 to 50.15 (0.84 Å)
Index ranges	−20 ≤ h ≤ 19<br>−20 ≤ k ≤ 20<br>−28 ≤ l ≤ 28
Reflections collected	128907
Independent reflections	19017<br>R_{int} = 0.0839<br>R_{sigma} = 0.0502
Completeness to θ = 25.077°	99.5 %
Data / Restraints / Parameters	19017 / 4140 / 1821
Goodness-of-fit on F^2	1.036
Final *R* indexes [$I≥2σ(I)$]	R_1 = 0.0705<br>wR_2 = 0.1760
Final *R* indexes [all data]	R_1 = 0.1075<br>wR_2 = 0.2041
Largest peak/hole [eÅ^{-3}]	3.27/−2.21

Table S4 $[Co_2(GaTMP)_8]$ (**3**)

CCDC number	2496872
Empirical formula	$C_{72}H_{144}Co_2Ga_8N_8$
Formula weight	1797.56
Temperature [K]	100(2)
Crystal system	triclinic
Space group (number)	$P\overline{1}$ (2)
a [Å]	13.2859(9)
b [Å]	14.9936(11)
c [Å]	26.309(2)
α [°]	83.159(2)
β [°]	85.540(2)
γ [°]	66.514(2)
Volume [Å^3]	4769.8(6)
Z	2
ρ_{calc} [gcm^{-3}]	1.252
μ [mm^{-1}]	2.595
F(000)	1868
Crystal size [mm^3]	0.134×0.135×0.332
Crystal colour	black
Crystal shape	block
Radiation	MoK_α (λ=0.71073 Å)
2θ range [°]	3.99 to 50.05 (0.84 Å)
Index ranges	$-15 \le h \le 15$
	$-17 \le k \le 17$
	$-31 \le l \le 31$
Reflections collected	171250
Independent reflections	16845
	R_{int} = 0.0358
	R_{sigma} = 0.0179
Completeness to θ = 25.027°	99.9
Data / Restraints / Parameters	16845 / 0 / 843
Goodness-of-fit on F^2	1.128
Final *R* indexes [$I \ge 2\sigma(I)$]	R_1 = 0.0277<br>wR_2 = 0.0625
Final *R* indexes [all data]	R_1 = 0.0311<br>wR_2 = 0.0637
Largest peak/hole [eÅ^{-3}]	0.47/−0.34

Table S5 *Hauser*-Base

CCDC number	2496874
Empirical formula	$C_{33}H_{60}Cl_2Mg_2N_2O_2$
Formula weight	636.35
Temperature [K]	104(2)
Crystal system	monoclinic
Space group (number)	$P2_1/n$ (14)
a [Å]	7.6546(5)
b [Å]	11.7122(8)
c [Å]	20.4953(12)
α [°]	90
β [°]	95.190(2)
γ [°]	90
Volume [Å^3]	1829.9(2)
Z	2
ρ_{calc} [gcm^{-3}]	1.155
μ [mm^{-1}]	0.241
F(000)	692
Crystal size [mm^3]	0.074×0.155×0.298
Crystal colour	colourless
Crystal shape	rod
Radiation	MoK_α (λ=0.71073 Å)
2θ range [°]	5.53 to 52.87 (0.80 Å)
Index ranges	$-9 \le h \le 9$<br>$-14 \le k \le 14$<br>$-25 \le l \le 25$
Reflections collected	98836
Independent reflections	3758<br>R_{int} = 0.0660<br>R_{sigma} = 0.0229
Completeness to θ = 25.242°	99.7
Data / Restraints / Parameters	3758 / 72 / 231
Goodness-of-fit on F^2	1.054
Final *R* indexes [$I \geq 2\sigma(I)$]	R_1 = 0.0325<br>wR_2 = 0.0843
Final *R* indexes [all data]	R_1 = 0.0372<br>wR_2 = 0.0880
Largest peak/hole [eÅ^{-3}]	0.32/−0.20

Table S6 [Fe_2(GaTMP)$_9$] (**4**)

CCDC number	2521089
Empirical formula	$C_{81}H_{162}Fe_2Ga_9N_9$
Formula weight	2001.37
Temperature [K]	100(2)
Crystal system	monoclinic
Space group (number)	$P2_1/n$ (14)
a [Å]	13.2536(12)
b [Å]	16.1292(15)
c [Å]	45.725(4)
α [°]	90
β [°]	92.933(4)
γ [°]	90
Volume [Å^3]	9761.7(16)
Z	4
ρ_{calc} [gcm^{-3}]	1.362
μ [mm^{-1}]	2.770
F(000)	4168
Crystal size [mm^3]	0.041×0.075×0.163
Crystal colour	black
Crystal shape	plate
Radiation	MoK_α (λ=0.71073 Å)
2θ range [°]	3.98 to 50.05 (0.84 Å)
Index ranges	−15 ≤ h ≤ 15
	−19 ≤ k ≤ 19
	−54 ≤ l ≤ 54
Reflections collected	322507
Independent reflections	17215
	R_{int} = 0.1505
	R_{sigma} = 0.0440
Completeness to θ = 25.027°	99.9
Data / Restraints / Parameters	17215 / 1922 / 1302
Goodness-of-fit on F^2	1.095
Final *R* indexes [I≥2σ(I)]	R_1 = 0.0668<br>wR_2 = 0.1718
Final *R* indexes [all data]	R_1 = 0.0771<br>wR_2 = 0.1773
Largest peak/hole [eÅ^{-3}]	3.28/−0.83

Extended Discussion of Analytical Data

Discussion of $[Ga_{10}]H(CoCp^*)_6$ (2)

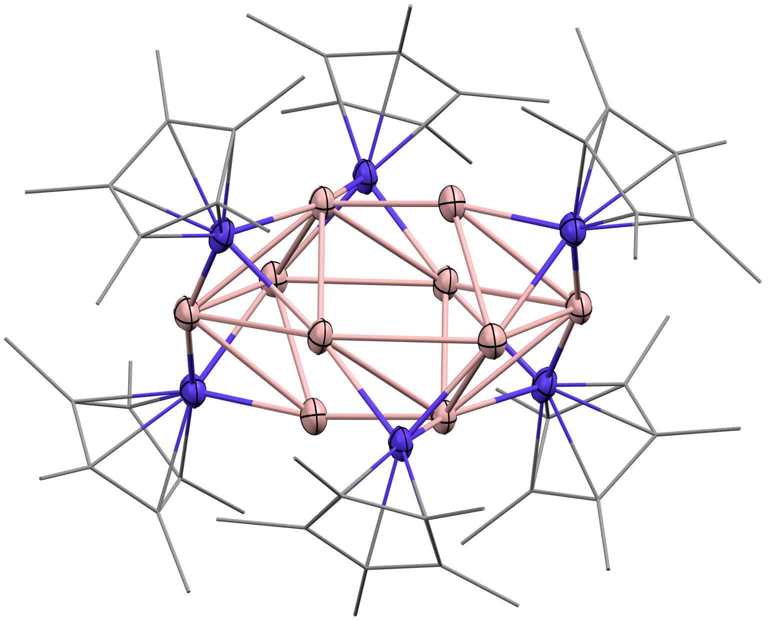

Figure S1 Molecular structure of $[Ga_{10}]H(CoCp^*)_6$ (**2**) in the solid state (thermal ellipsoids are given at 50% probability level). Cobalt in violet, gallium in rose. Selected bond lengths (Å) of $[Ga_{10}]H(CoCp^*)_6$: Ga-Ga (hexagonal plane, short side): 2.839(3), Ga-Ga (hexagonal plane, long side): 2.964(3)-2.965(3), Ga-Ga (plane-apex): 2.838(3)-2.876(3), Ga-Ga (apex-apex): 2.484(3), Co-Ga: 2.248(3)-2.407(3).

$[Ga_{10}]H(CoCp^*)_6$ (**2**) crystalizes in the monoclinic space group $P2_1/n$ and consists of a $[Ga_{10}]$ cluster core and a $(CoCp^*)_6$ shell. The cluster core can be described as the conjunction of two capped trigonal prisms *via* their shared square planes. The capping atoms of the trigonal prisms are on opposite sides to one another.

Alternatively, the cluster core can be described as a hexagonal bipyramid with two vertices as the apices of the bipyramidal structure. Therefore, the cluster core can be imagined as two fused pentagonal bipyramidal structures. Based on the latter description, the distances of selected bonds are the following: The Ga-Ga distances of the cluster core range from 2.484(3) to 2.965(3) Å. For a detailed presentation of the bond distances, see Figure S1. The six CoCp* ligands bind face-capping on the triangular planes of the bipyramid-derived cluster core, three on each side of the cluster core. The Co-Ga distances of the core towards the CoCp* ligands range from Co-Ga: 2.248(3)-2.407(3).

2 was only obtained by crystal picking from a mixture of different clusters of which a total of 10 species could be identified upon analysis by LIFDI-MS (Table S1 and Figures S50 and S51). The composition of all of the identified clusters is described by the formula $[Ga_xCo_y](CoCp^*)_z$ with x = 5-11, y = 0,1, z = 6,7. Although the exact molecular structures of the clusters remain uncertain, some assumptions can be

made based on the findings of the molecular structure of **2**: The cluster ensemble consist most probably of pairs of cluster with a [Ga_{5-11}] core and a counterpart with a [$Ga_{5-11}Co$] core, surrounded by a $(CoCp^*)_6$ shell. One exception is the cluster core pair of [Ga_{11}]/[$Ga_{11}Co$], where the all-cobalt shell consists of seven instead of six CoCp* moieties, due to the enlarged cluster core of eleven and twelve metal atoms, respectively.

The cluster ensemble was further characterized by ^1H, ^{13}C, HSCQ, HMBC NMR as well as IR spectroscopy. The IR spectrum of the cluster library shows a distinct vibrational band at 1782 cm^{-1} and a second, smaller band at 1648 cm^{-1} (Figure S44). The first can be allocated to a gallium hydride, bound to a gallium of the cluster core (see hydride species of **1** at 1844 cm^{-1}), whereas the vibrational band of lower intensity is rather a bridging hydride.[8] The presence of a cobalt hydride can be excluded, since such hydrides are expected at vibrational band of wavenumbers starting at 1900 cm^{-1}.[8-9] This is in accordance of the sum formulas of the clusters of the ensemble derived from the LIFDI mass spectra, which also indicate the presence of hydride-bearing clusters, such as **2**.

Beside the crystal structure of **2**, also the NMR spectra of the cluster ensemble indicate a structure resulting from ligand transfer from the gallium towards the cobalt. The ^1H and ^{13}C NMR spectra mainly show the signals of the methyl groups of the Cp* ligands shell, whereas with the ^1H ^{13}C HMBC spectrum, also the carbon atoms of the aromatic ring of the Cp* ligands can be observed and allocated (Figures S5 to S11). The majority of those carbon atoms are observed at a chemical shift of 85-90 ppm, which is in accordance to the typical chemical shift of Co-Cp* units, which are expected at shifts of 85-95 pm,[10-11] whereas the ^{13}C signals of the Cp*-rings of Ga-Cp* ligands are observed at chemical shifts of 110 to 125 ppm.[11-14]

In fact, this cluster ensemble mirrors the cluster formation of the molecular Ni/Ga cluster ensemble of the form [Ga_{6+x}]$Ni_{0+y}(NiCp^*)_6$ (with $x + y \leq 2$). Again, ligand transfer from gallium towards nickel leads to gallium-only cluster cores of different sizes and cores containing one or two additional nickel atoms, where the cluster shell is again made up of six NiCp* moieties.[15]

Discussion of $[Co_3Ga_2]H(GaTMP)_9$ (1)

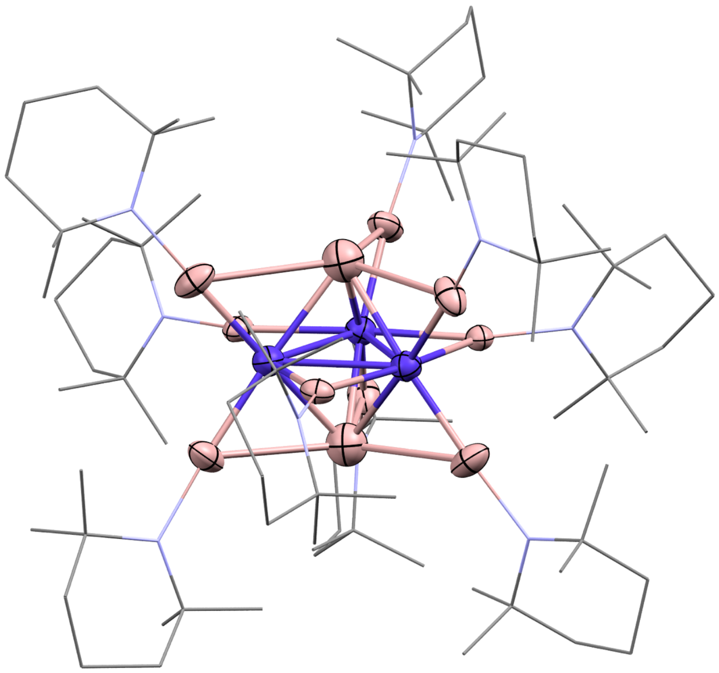

Figure S2 Molecular structure of **1** in the solid state (thermal ellipsoids are given at 50% probability level). Cobalt in violet, gallium in rose. Selected bond lengths (Å) and angles (deg) of **1**: Co-Co: 2.669(3)-2.673(2) Å, Co-μ^3-Ga: 2.382(3)-2.389(2), Co-μ^2-Ga (CoCo-bridging): 2.347(2)-2.352(2), Co-μ^2-Ga (CoGa-bridging): 2.176(2)-2.180(2), Ga-μ^2-Ga (CoGa-bridging): 2.922(3)-2.927(2), Co-Co-Co: 59.94°-60.07°, Co-Co-μ^3-Ga: 55.87°-56.10°, Co-Co-μ^2-Ga (CoCo-bridging): 55.32°-55.41°, Ga-Co-μ^2-Ga (CoGa-bridging): 79.54°-79.63°, Co-Ga-μ^2-Ga (CoGa-bridging): 46.97°-47.15°.

1 crystalizes in the triclinic space group P-1 and its molecular structure can be viewed at as a heterobimetallic cluster core of a trigonal bipyramid structure, which is coordinated by a 4,4,4-tricapped trigonal prism – a nine-vertex deltahedron – coordination sphere of nine GaTMP ligands. The cluster core consists of a central cobalt triangle with Co-Co distances of 2.669(3)-2.673(2) Å, capped by two TMP-free gallium atoms with Ga-Co distances ranging from 2.382(3) to 2.389(2) Å and a Ga-Ga distance of 3.640 Å. Cobalt atoms arranged in triangular fashion are commonly found in literature of organometallic cobalt chemistry, such as in $Co_3(\mu^3\text{-}CCl)(CO)_9$,[16] $Co_3Cp^*{}_3(CH)_2$[17] and $Co_3Cp^*{}_3O_2$,[18] for instance. The Co-Co distances of 2.669(3)-2.673(2) Å in **1** are significantly longer compared to the literature-known organometallic compounds containing Co-triangles (2.439 Å in $Co_3Cp^*{}_3CH_2$ and 2.438 Å in $Co_3Cp^*{}_3O_2$, for instance).

This central unit is coordinated by three Co_2-edge bridging μ^2-GaTMP, in plane with the cobalt triangle, and in total six CoGa-edge bridging μ^2-GaTMP, three in plane with one of the terminal core-gallium atoms each. The bonds towards those CoGa-edge bridging GaTMP (av. 2.286 Å) are longer than the

CoCo bridging (av. 1.964 Å), when looking at the distance of the GaTMP towards the centroid of its coordination site.

This can be explained by the steric constrain of the TMP ligand, resulting in a tilting of the GaTMP ligand outwards the cluster and in a less isosceles triangle of the ligand with the CoGa-coordination site of the core. This is reflected by the different ligand distances of av. 2.228 Å and av. 2.925 Å for the Co-Ga and Ga…Ga distances (CoGa-bridging ligands), respectively and av. 2.350 Å for the Co-Ga distances (CoCo-bridging ligands), which is further resulting in altered angle of those triangles (av. 55.35° for the Ga-Co-Co angles of the Co_2-bridging and av. 47.09° and 79.59° for the Ga-Ga-Co and Ga-Co-Ga angles of the CoGa-bridging ligands). The Ga(apex)…GaTMP separations are substantially larger than what is generally considered as a single bond distance (~ 2.5-2.6 Å),[19] but substantially lower than twice the van der Waals radius of gallium (1.87 Å).[20] The nature of these weak interaction will be analyzed in more details below in the section dealing with the bonding analysis of **1** by DFT calculations. Those six CoGa- edge bridging GaTMP can be viewed as a trigonal prim, which is capped by the three Co_2-edge bridging μ^2-GaTMP, leading to an overall 4,4,4-tricapped trigonal prism structure of the ligand sphere. The central plane of **1** ("$(Co_3)(\mu^2\text{-GaTMP})_3$") is structurally related to the intermetallic cluster $Ni_3(GaTMP)_7$, which exhibits a central $(Ni_3)(\mu^2\text{-GaTMP})_3$ building unit.[2] Besides that, there are no further TM/E-TMP clusters or complexes of a comparable size that **1** might be compared to.

1 was further characterized beside SC-XRD. The ^1H and ^{13}C chemical shifts of **1** are all in a similar range with respect to $Ni_3(GaTMP)_7$.[2] The ^1H NMR gives rise to three different distinct signals of the GaTMPs methyl groups in the ratio of 1:1:1, representing the three Co_2-edge bridging μ^2-GaTMP and each the three CoGa- edge bridging GaTMP separated by the Co_2 bridging ligands (Figures S12 and S13). The signals of the backbone of the TMP ligands are not fully separated to three signals, which is a similar behavior as for the cluster $Ni_3(GaTMP)_7$, which is also containing GaTMP ligands of different bridging modes. Besides, also TMPH as decomposition product of **1** is visible in the ^1H NMR spectrum, since **1** slowly decomposes in solution over time against all tested solvents. Formed TMPH is overrepresented in the spectrum due to the low solubility of **1**. Traces of hexane/THF are also visible as media of synthesis and crystallization and due to co-crystallization. All ^1H signals were allocated by ^{13}C, COSY and HSQC NMR spectroscopy. The shifted methyl signals of three of the CoGa-edge bridging GaTMP are due to an altered electronic structure of one of the pyramidal gallium atoms, caused by a hydride bound to it. We were not able to observe this Ga-bond hydride in the ^1H NMR spectrum, due to the assumed fluxionality of the gallium hydride. Even upon variable temperature ^1H NMR spectroscopy, the hydride could not be observed at the expected chemical shift, which was determined by the DFT calculations of the NMR chemical shifts of **1** (6.4 ppm, *vide infra*) and observed for ^2H NMR of **1-D** (6.60 and 6.49 ppm, *vide infra*). The ^{13}C NMR signal of the carbon atoms in β-position of the TMP ligand are

not visible in the ^{13}C NMR spectrum (Figure S14 and S15), due to the already mentioned low solubility of **1**, and were determined *via* 2D-NMR spectroscopy (Figures S16 and S19). Nevertheless, Figures S17 and S18 shows an additional ^{13}C NMR spectrum from a *Bruker* AV-II-500 equipped with a cryo probe head device with all ^{13}C signals detected but with a not identified impurity.

The presence and location of the mentioned hydride was further confirmed by IR spectroscopy, revealing a Ga-H vibrational band at 1844 cm^{-1} (Figure S45). This vibrational band is in accordance to the DFT calculations of **1**, which predicted a band at 1854 cm^{-1} (gas phase calculations). Moreover, also the mass spectrometric analysis by LIFDI-MS, an extremely soft ionization technique, able to be used under inert atmosphere, showing a *m/z* of 2207.2822, which is in great agreement to the sum formula $Co_3Ga_{11}TMP_9H$ (calc.: 2207.28215, Figures S54 to S56).

Additionally, preparative synthesis of **1** performed under D_2 atmosphere instead of H_2 led to the quantitative formation of $[Co_3Ga_2]D(GaTMP)_9$ (**1-D**), where the position of the single deuteride is determined by MS, IR and NMR spectroscopy. The IR measurement of **1-D** led to a disappearance of the vibrational band at 1844 cm^{-1} of the Ga-H in the spectrum. Since in the region, where the Ga-D would be expected, are many different vibrational bands (mainly C-C and C-H vibration), the Ga-D band is assumed to be a new arisen band and at 1259 cm^{-1} (Figure S46 and S47). This is further in agreement with the vibrational band predicted by DFT (1317 cm^{-1}). The mass spectrum of the compound shows a shift of 1 *m/z*, confirming the isotopic labeling of the hydridic position by deuterium leading to $Co_3Ga_{11}TMP_9D$ (*m/z* = 2208.2763), again in agreement with the theoretically expected *m/z* (calc.: 2208.28843, Figure S57 and S58). These results are in line with the ^{2}H-NMR spectrum of **1-D**, showing two signal at 6.60 and 6.49 ppm (Figures S20 and S21), again in agreement with the predicted ^{1}H-NMR-shift of the Ga-H at 6.4 ppm by DFT calculations (^{1}H-and ^{2}H-NMR shifts can be considered quite similar in general). The reason for the observation of two signals instead of one is not the resolution of the Ga-D coupling, which would result in a much more complicated multiplet, but rather the resolution of **1-D**'s Ga-D signals of the two different naturally abundant gallium isotopes of ^{69}Ga and ^{71}Ga. In fact, the integral ratio of the two signals reflects the natural isotopic ratio of ^{69}Ga and ^{71}Ga of 60:40 (at 6.49 and 6.60 ppm, respectively). Upon formation of isotopically pure ^{71}Ga-TMP and subsequently, by the synthesis of the cluster **1-D** labeled with isotopically pure ^{71}Ga-TMP, only one signal was observed in the ^{2}H NMR spectrum at a chemical shift of 6.62 ppm, which is consistent with the signal of the integral representing the natural abundance of ^{71}Ga (40%) in the initial ^{2}H NMR consisting of the isotopic mixture of both Ga isotopes in cluster **1-D** (Figures S20-S21 and S22-S23). We were not able to identify comparable cases for the isotopic resolution of Ga-H or Ga-D in molecular chemistry, only in solid-state chemistry.[21-22]

Investigation of semihydrogenation of 3-hexyne towards 3-hexene using **1** as catalyst in the presence of H_2 was investigated *via* ^{1}H NMR spectroscopy and LIFDI mass spectrometry. No conversion of 3-

hexyne towards hexene or hexane could be observed according to ^{1}H NMR spectroscopy. Besides, also no reaction intermediates such as **1** bearing hexenyl ligands could be identified upon *in situ* LIFDI mass spectrometry. Moreover, the activation of dihydrogen (H_2) at **1** was investigated by reaction of **1** with D_2. Again, no D_2 activation and no H/D exchange could be observed upon analysis *via in situ* LIFDI mass spectrometry.

Discussion of $Co_2(GaTMP)_8$ (3)

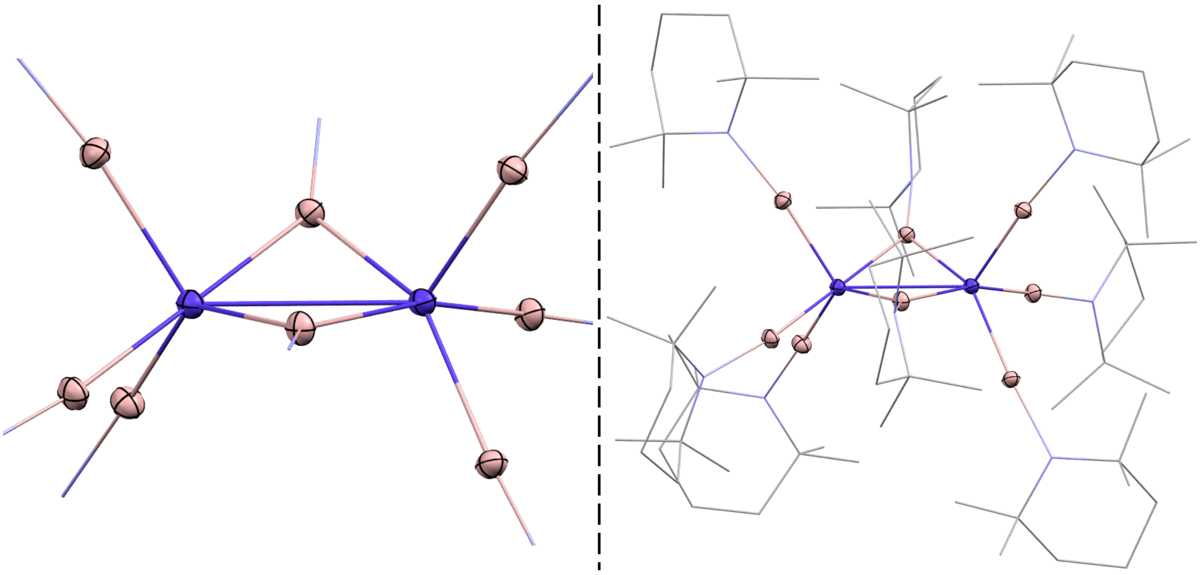

Figure S3 Molecular structure of **3** in the solid state determined by single crystal x-ray diffraction (right) and with ligands omitted (left, thermal ellipsoids are given at 50% probability level). Co: violet, Ga: rose, TMP ligands are omitted for clarity. Selected bond lengths (Å) and angles (deg): Co-Co: 2.807(1), Co-Ga: 2.173(3)-2.194(1), Co-μ^2-Ga: 2.305(3)-2.335(7), Co-Co-μ^2-Ga: 52.29°-53.28°, Co-Co-Ga: 112.72°-129.01°.

3 is a decanuclear Co/Ga compound and a structural analogue to $Co_2(CO)_8$. The center of the complex consists of two Co^0 atoms in a distance of 2.807(1) Å. Despite the elongated Co-Co distance of **3** (2.807(1) Å), compared to $Co_2(CO)_8$ (2.530 Å),[23] and which is rather similar to the distance in $Co_2(CO)_6(\mu^2$-GaTMP$)_2$ (2.836 Å),[24] where the GaTMP function as the bridging and CO as the terminal ligands, Co-Co interactions are still assumed in **3**.

The complex shows a diamagnetic behavior upon ^{1}H-NMR spectroscopic analysis (see Figures S24 to S26) and therefore, there are no unpaired electrons of the two Co^0 d^9 centers assumed. A similar behavior could be observed for $Co_2(CO)_6(\mu^2$-GaTMP$)_2$, which is also a diamagnetic compound. Besides, the distance of **3** of 2.807(1) Å is still lower than the combined van der Waals radii of the two cobalt

atoms and is not significantly shorter than the Co-Co distances in **1** with 2.669(3)-2.673(2) Å (*vide supra*).[20]

Three of the in total eight GaTMP ligands are coordinated in a terminal fashion towards the two cobalt centers each (Co-Ga: 2.173(3)-2.194(1) Å), with the remaining two GaTMP ligands coordinated in a bridging fashion between the Co (Co-μ^2-Ga: 2.305(3)-2.335(7) Å). The central 4-atom square is not planar, since the two bridging GaTMP are tilted upwards compared to the two cobalt atoms, which can be seen at the angles of this central square of 52.29°-53.28° (Co-Co-μ^2-Ga). The coordination around one cobalt atom can be viewed at as a polyhedral form laying between a trigonal bipyramid and a tetragonal pyramid. The angles between the Co-Co bond towards the terminal GaTMPs are in a range of 112.72°-129.01°. **3** was further characterized by ^{1}H, ^{13}C, DEPT-135, COSY, HSQC NMR and IR spectroscopy, as well as LIFDI mass spectrometry (see Figures S24 to S32, S48, S63 and S64).

$Fe_2(GaTMP)_9$ (4)

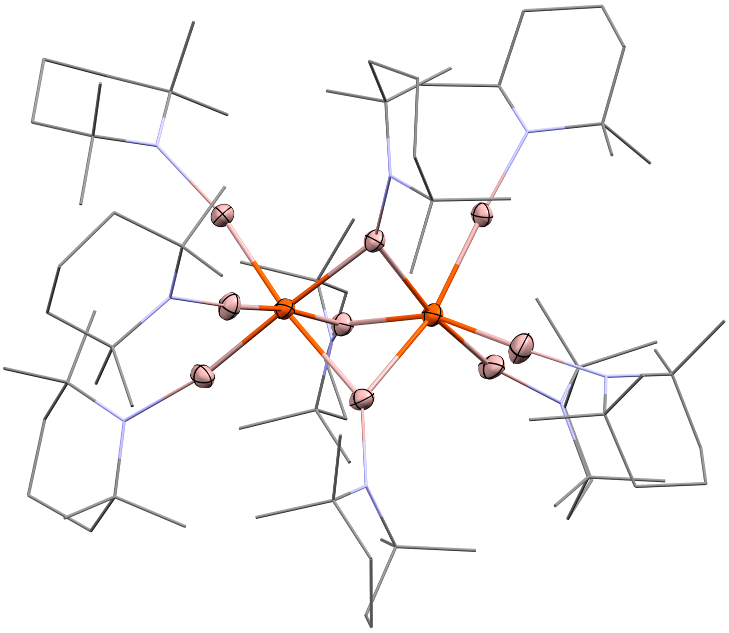

Figure S4 Molecular structure of **4** in the solid state determined by single crystal x-ray diffraction (thermal ellipsoids are given at 50% probability level). Fe: orange, Ga: rose. Selected bond lengths (Å) and angles (deg): Fe-Fe: 2.912(1), Fe-Ga: 2.228(1)-2.241(1), Fe-μ^2-Ga: 2.389(1)-2.433(1), Fe-Fe-μ^2-Ga: 74.11°-74.49°, Fe-Fe-Ga: 115.45°-131.68°.

Analogously to the structure of **2**, **4** is a hendecanuclear Fe/Ga complex, which possesses a structure analogue to Fe_2CO_9.[25] In its center, **4** is build up by two Fe^0 atoms with a Fe-Fe distance of 2.912(1) Å. In contrast, Fe_2CO_9 has a much shorter Fe-Fe distance of 2.4609 Å, again similar to the Co-Co distance of Co_2CO_8 and its Co/Ga counterpart (**2**).[26]

Unlike the formation of $Co_2(CO)_6(\mu^2\text{-GaCp*})_2$ from $Co_2(CO)_8$, $Fe_2(CO)_6(\mu^2\text{-GaCp*})_3$ cannot be obtained by the reaction of $Fe_2(CO)_9$ with GaCp*. Instead, the pentanuclear iron complex $Fe(CO)_4(GaCp*)$ is formed, where the GaCp* takes the axial position in the bipyramidal structure of the complex.[1] Nevertheless, $Fe_2(CO)_6(\mu^2\text{-GaCp*})_3$ was reported following a synthesis starting from $Fe(CO)_3(COT)$ (COT = cyclooctatetraene).[1] Again, the three bridging CO ligands were replaced by GaCp* units, resulting in an increased Fe-Fe bond length from 2.4609 to 2.908(6) Å. Beside $Fe_2(CO)_6(\mu^2\text{-GaCp*})_3$, also the two complexes $Fe_2(CO)_7(\mu^2\text{-Si(TMS)}_3)_2$ and $Fe_2(CO)_6(\mu^2\text{-Si(TMS)}_3)_3$ are known, where two and three bridging CO ligands were replaced with $Ga(Si(TMS)_3)$ units (Fe-Fe distances of 2.6804(8) and 2.876(2) Å, respectively).[27] An analogue complex containing GaTMP as bridging ligand is not known yet.

4 consists of a central trigonal bipyramidal $[Fe_2Ga_3]$ unit, made up by the iron atoms and the gallium atoms of the bridging GaTMP ligands. The distance of the iron atoms towards the bridging gallium atoms is 2.389(1)-2.433(1) Å and the angle between the central Fe-Fe bond towards the bridging gallium atoms ranges from 74.11°-74.49°. Besides, the distance of the iron centers towards the in total six terminal GaTMP ligands is 2.228(1)-2.241(1) Å, whereas the angle between the Fe-Fe bond towards the terminal GaTMP units is 115.45°-131.68°.The coordination sphere of the GaTMP around the iron centers can be seen as an octahedron. **4** was further characterized by ^1H, ^{13}C, COSY, HSQC and HMBC NMR spectroscopy as well as LIFDI mass spectrometry (see Figures S35 to S40, S75 and S78).

Characterizing **4** by LIFDI-MS led only to a low intensity of the obtained patterns, but both the molecular ion as well as most prominent fragment ions were visible and in good agreement with the theoretically predicted isotopic pattern for $[M]^+$ (M = $Fe_2(GaTMP)_9$, *m/z* [a.u.] = 2001.5098, calc.: 2001.49470), $[M\text{-GaTMP}]^+$ (*m/z* [a.u.] = 1790.4278, calc.: 1790.42533) and $[M\text{-(GaTMP)}_3]^+$ (*m/z* [a.u.] = 1370.2768, calc.: 1370.28642, see Figures S75 to S78).

The isolated compound was not pure **4**, which can be seen in the ^1H NMR spectrum, showing the isolated product being a mixture of $Fe_2(GaTMP)_9$, $Fe(GaTMP)_5$ (as a side product) and GaTMP (due to incomplete conversion). The ^1H signals could be allocated using ^1H ^1H COSY NMR spectroscopy, and the ^{13}C chemical shifts were determined by ^1H ^{13}C HSQC and ^1H ^{13}C HMBC NMR spectroscopy (see Figures S35 to S40).

NMR Spectra

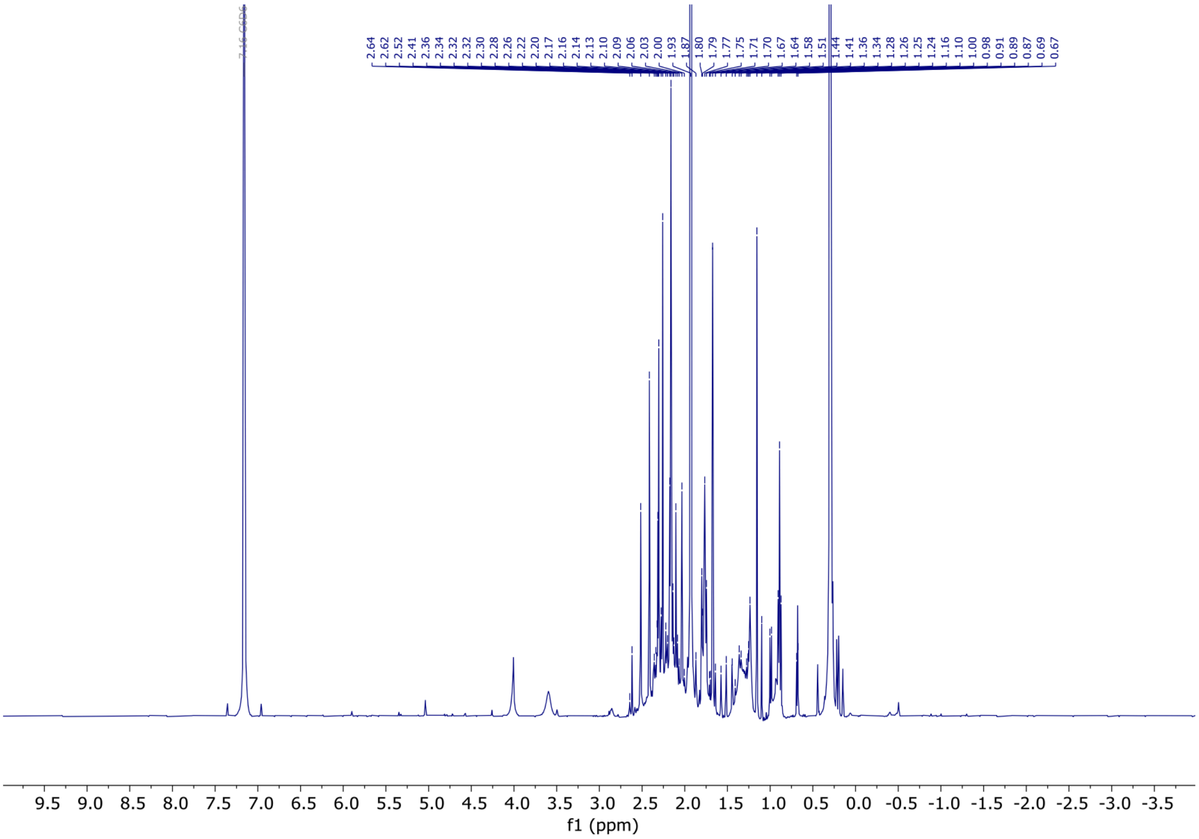

Figure S5 ^{1}H NMR spectrum of the Co/Ga cluster library $[Ga_xCo_y](CoCp^*)_z$ (x = 5-11, y = 0,1 and z = 6,7) in benzene-d_6 (full range spectrum).

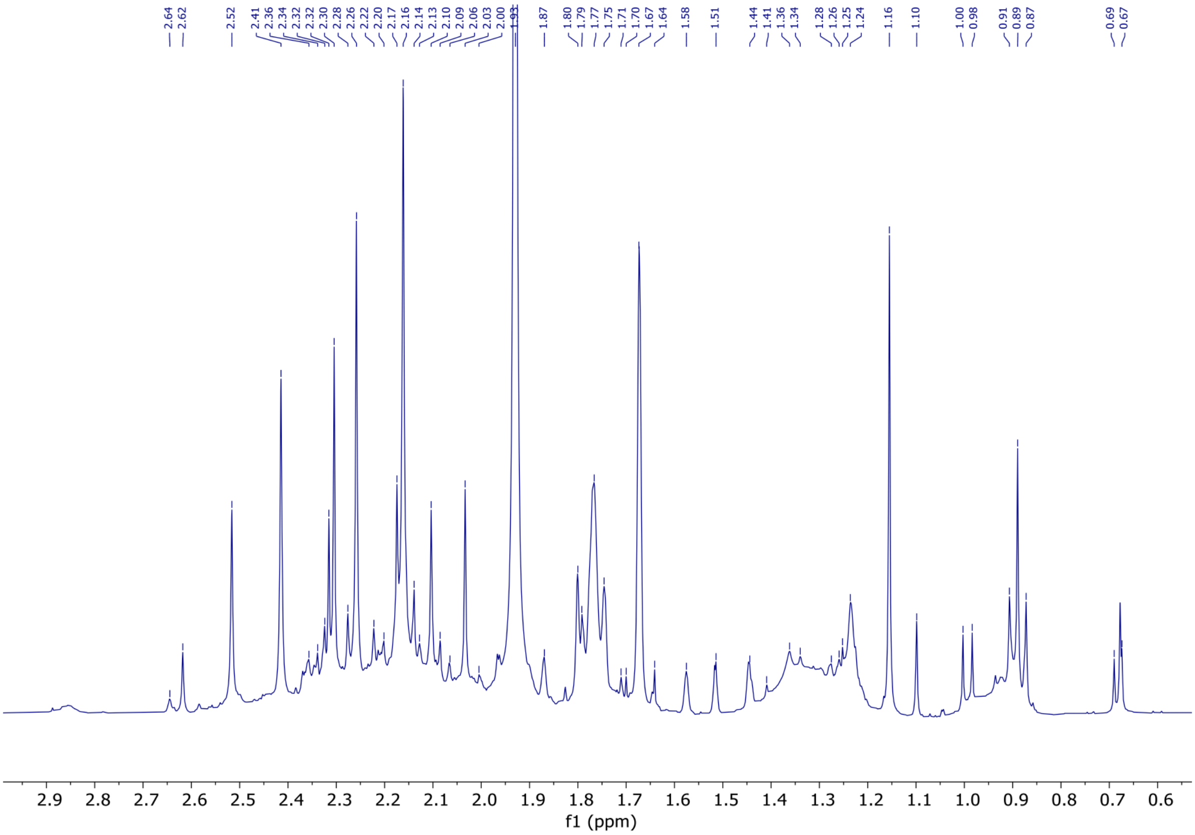

Figure S6 ^{1}H NMR spectrum of the Co/Ga cluster library $[Ga_xCo_y](CoCp^*)_z$ (x = 5-11, y = 0,1 and z = 6,7) in benzene-d_6 (close-up spectrum).

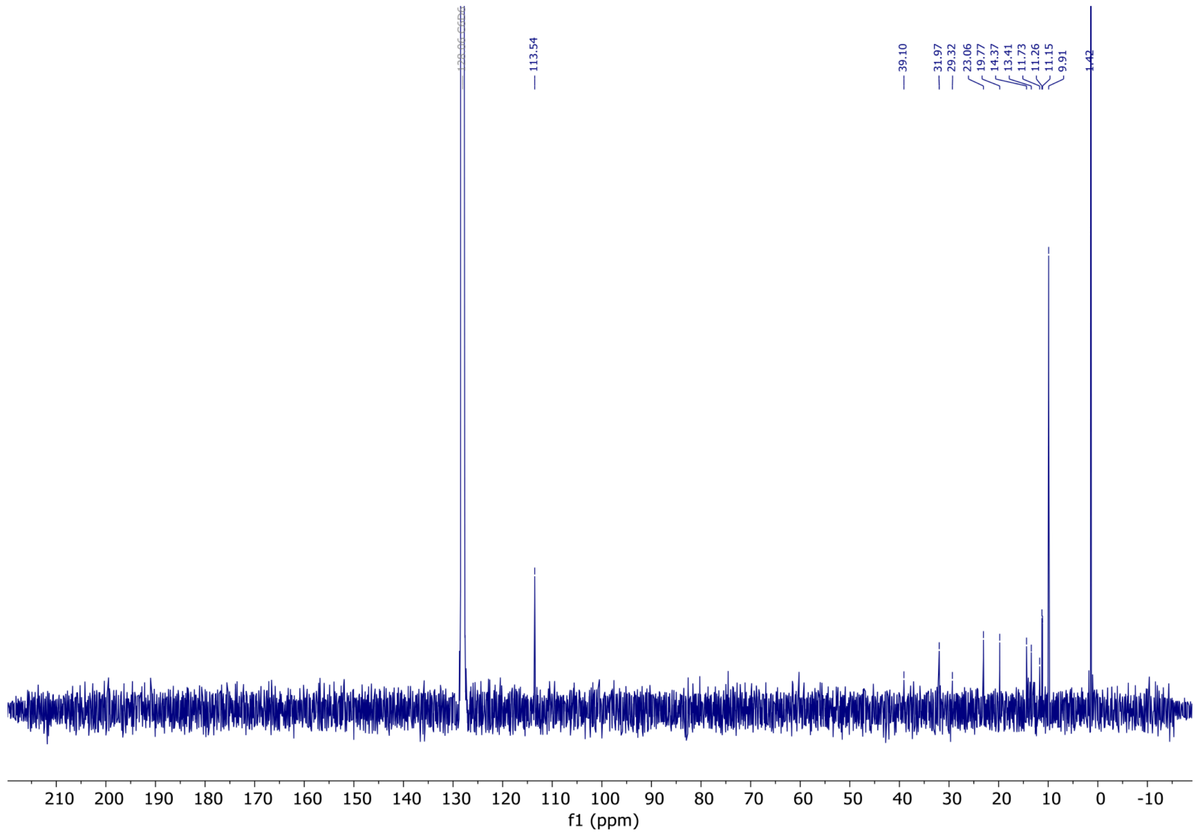

Figure S7 ^{13}C NMR spectrum of the Co/Ga cluster library $[Ga_xCo_y](CoCp^*)_z$ (x = 5-11, y = 0,1 and z = 6,7) in benzene-d_6.

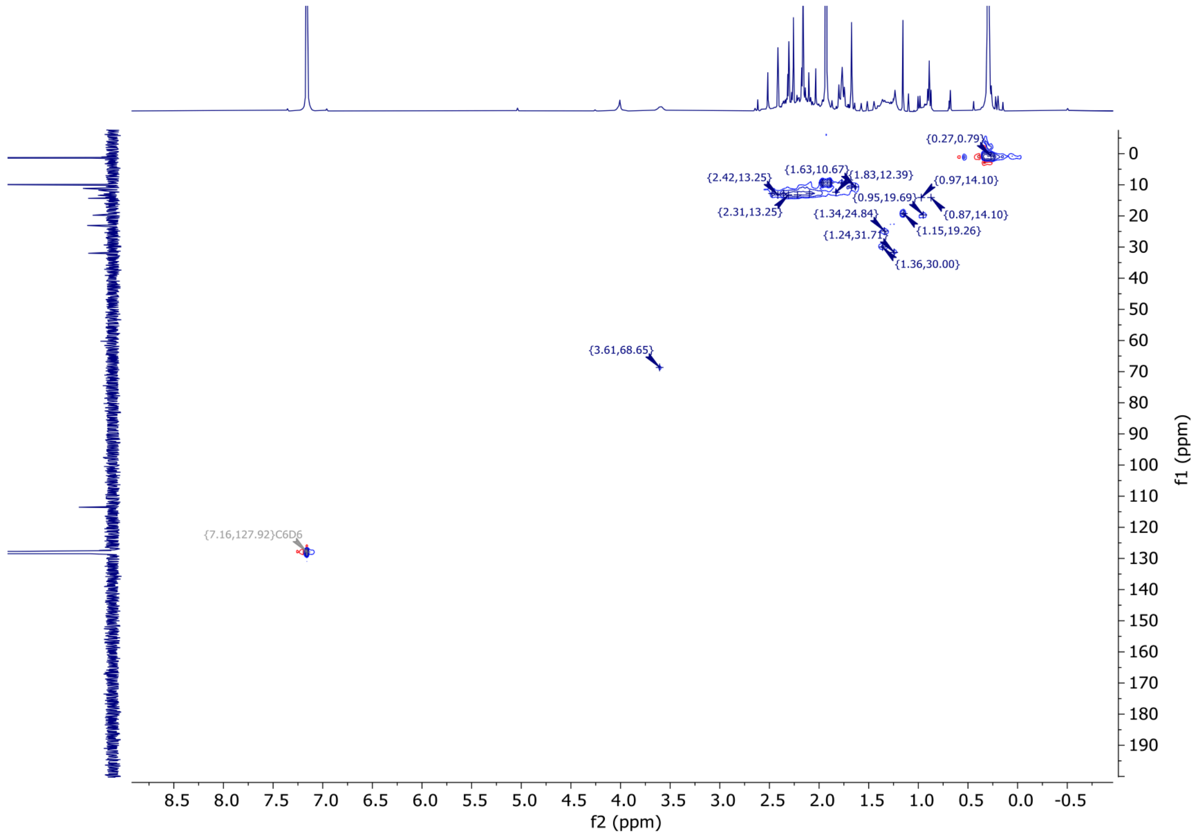

Figure S8 ^{1}H ^{13}C HSQC NMR spectrum of the Co/Ga cluster library $[Ga_xCo_y](CoCp^*)_z$ (x = 5-11, y = 0,1 and z = 6,7) in benzene-d_6 (full range spectrum).

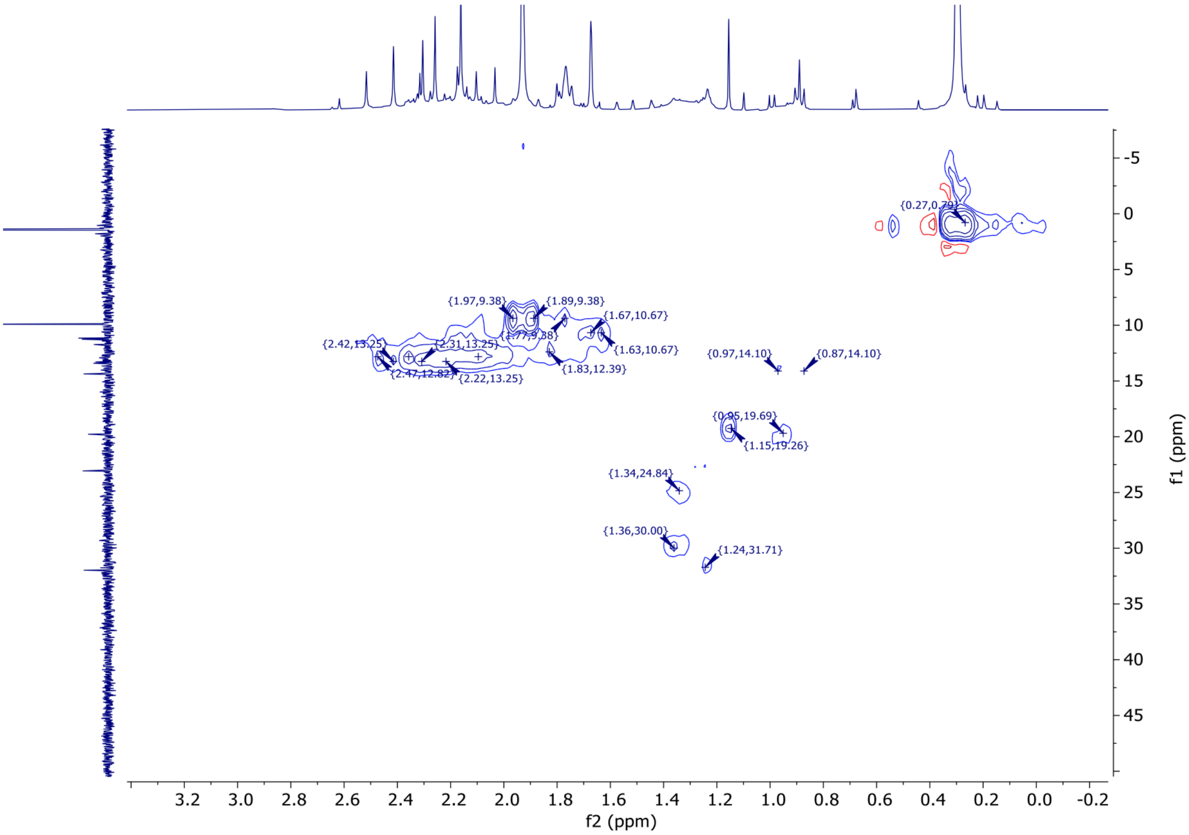

Figure S9 ^{1}H ^{13}C HSQC NMR spectrum of the Co/Ga cluster library $[Ga_xCo_y](CoCp^*)_z$ (x = 5-11, y = 0,1 and z = 6,7) in benzene-d_6 (close-up spectrum).

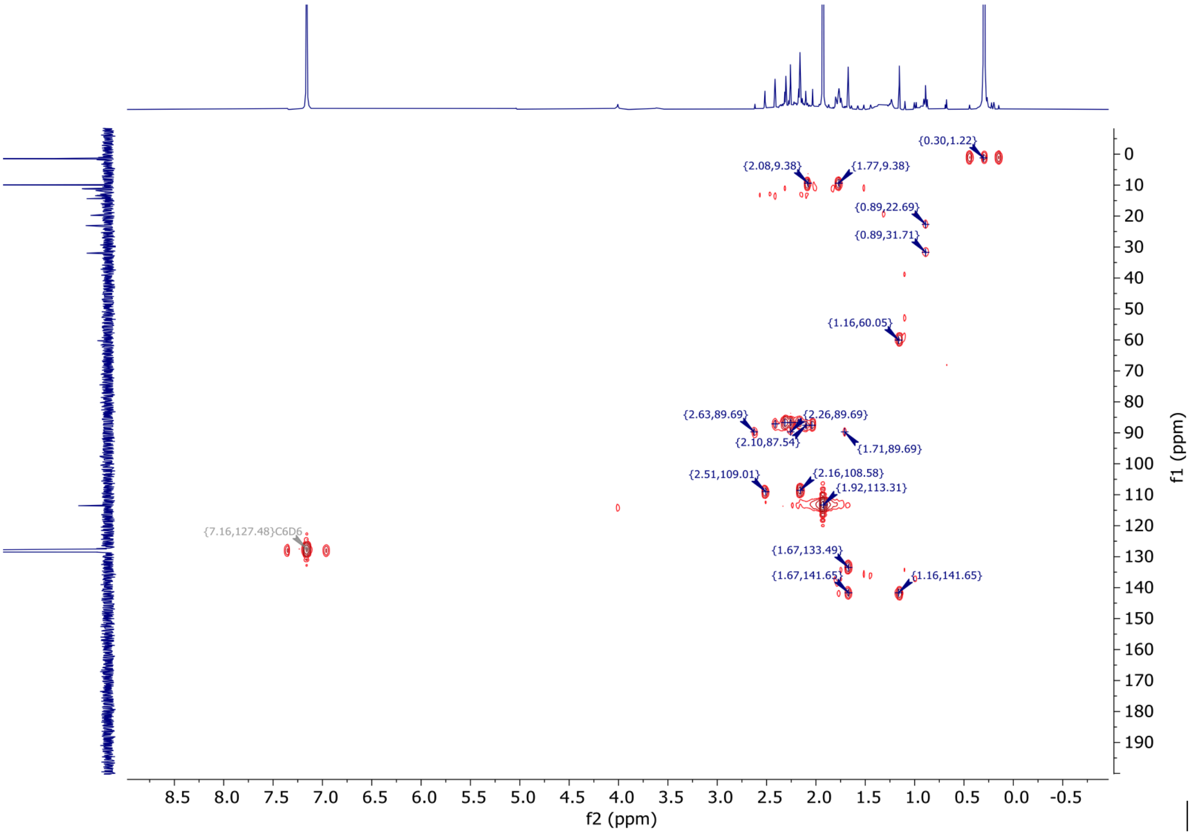

Figure S10 ^{1}H ^{13}C HMBC NMR spectrum of the Co/Ga cluster library $[Ga_xCo_y](CoCp^*)_z$ (x = 5-11, y = 0,1 and z = 6,7) in benzene-d_6 (full range spectrum).

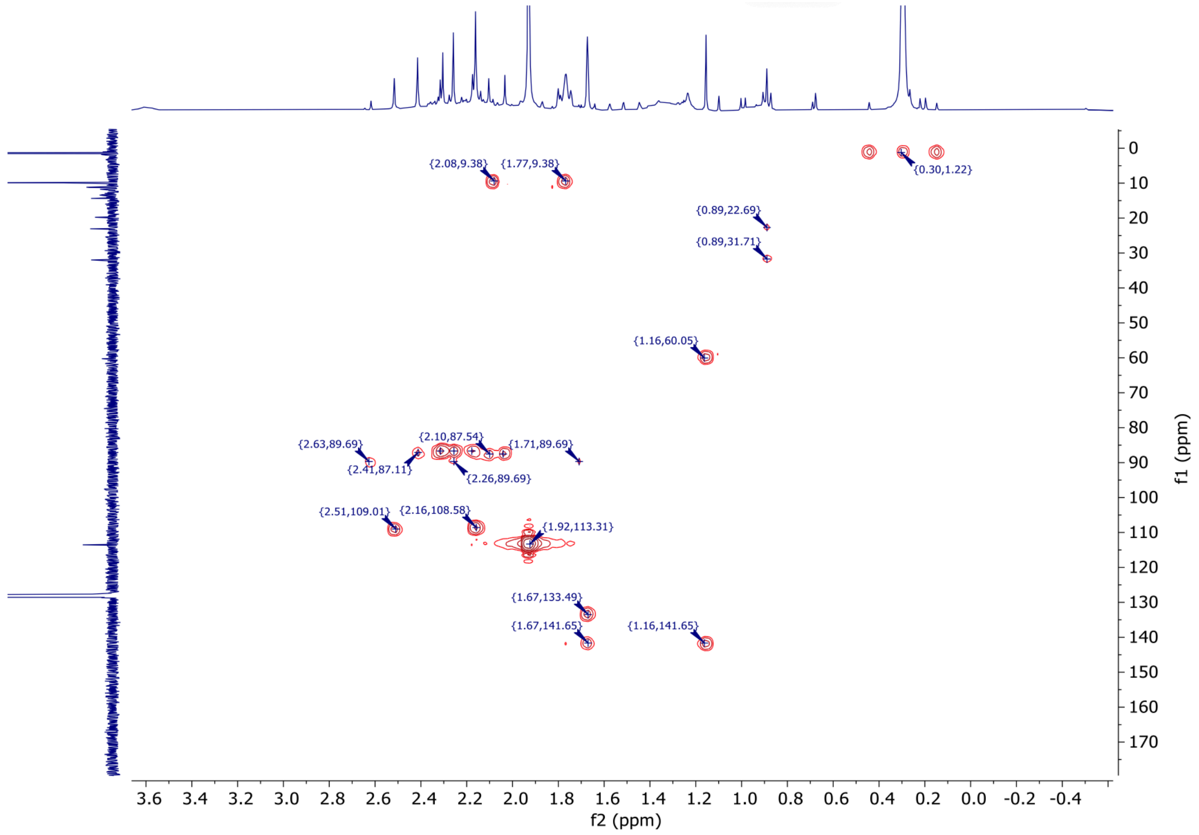

Figure S11 ^{1}H ^{13}C HMBC NMR spectrum of the Co/Ga cluster library $[Ga_xCo_y](CoCp^*)_z$ (x = 5-11, y = 0,1 and z = 6,7) in benzene-d_6 (close-up spectrum).

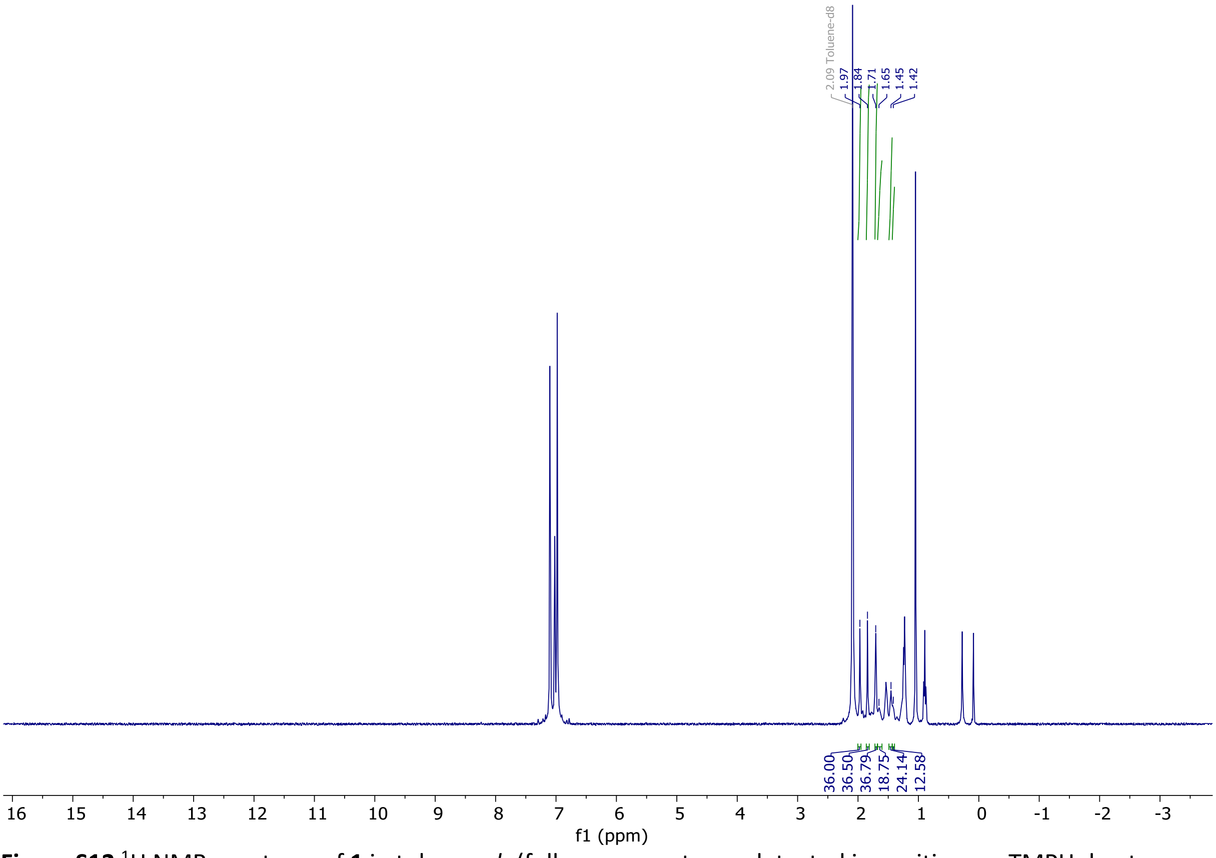

Figure S12 ^{1}H NMR spectrum of **1** in toluene-d_8 (full range spectrum; detected impurities are TMPH due to decomposition of small amounts of the compound upon contact with the solvent, hexane as crystallization medium and grease, see also Figure S13 below).

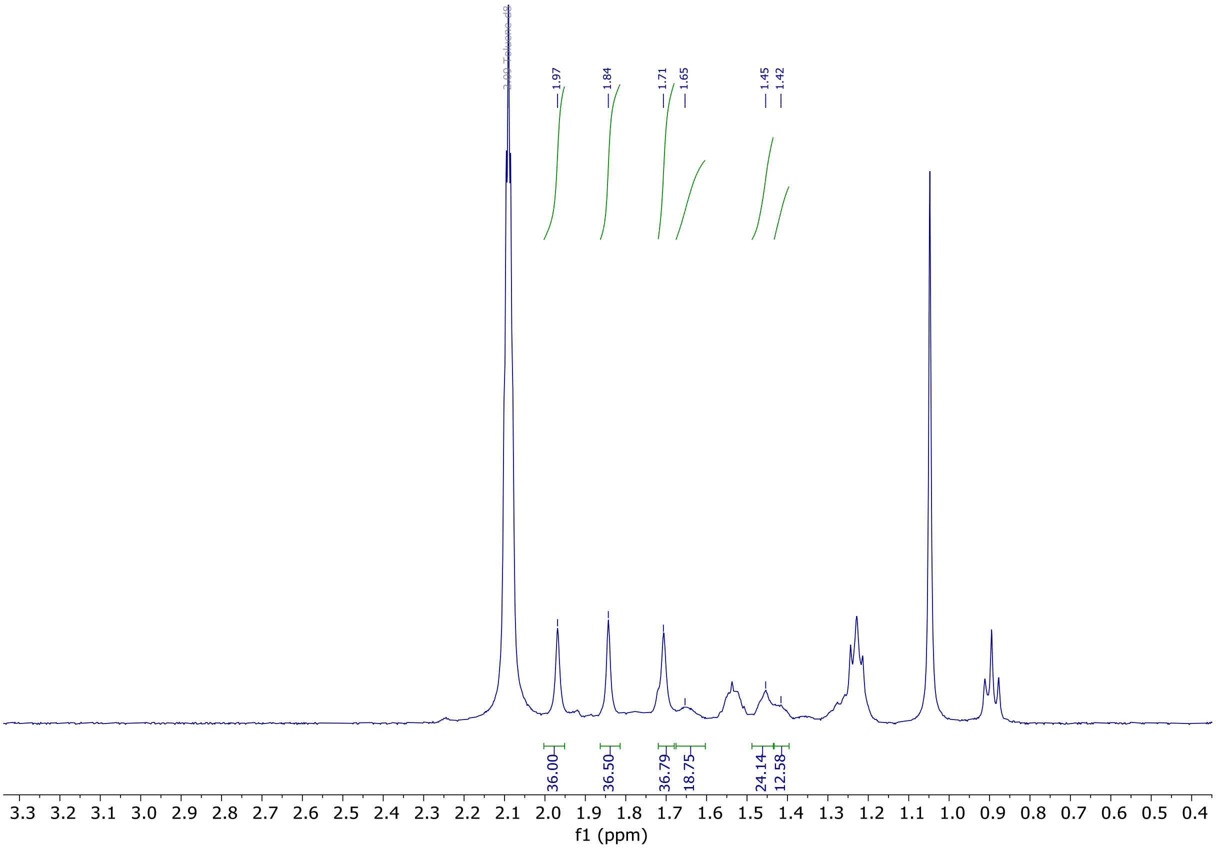

Figure S13 ^{1}H NMR spectrum of **1** in toluene-d_8 (close-up spectrum; detected impurities are TMPH due to decomposition of small amounts of the compound upon contact with the solvent, hexane as crystallization medium and grease, see also Figure S12 above).

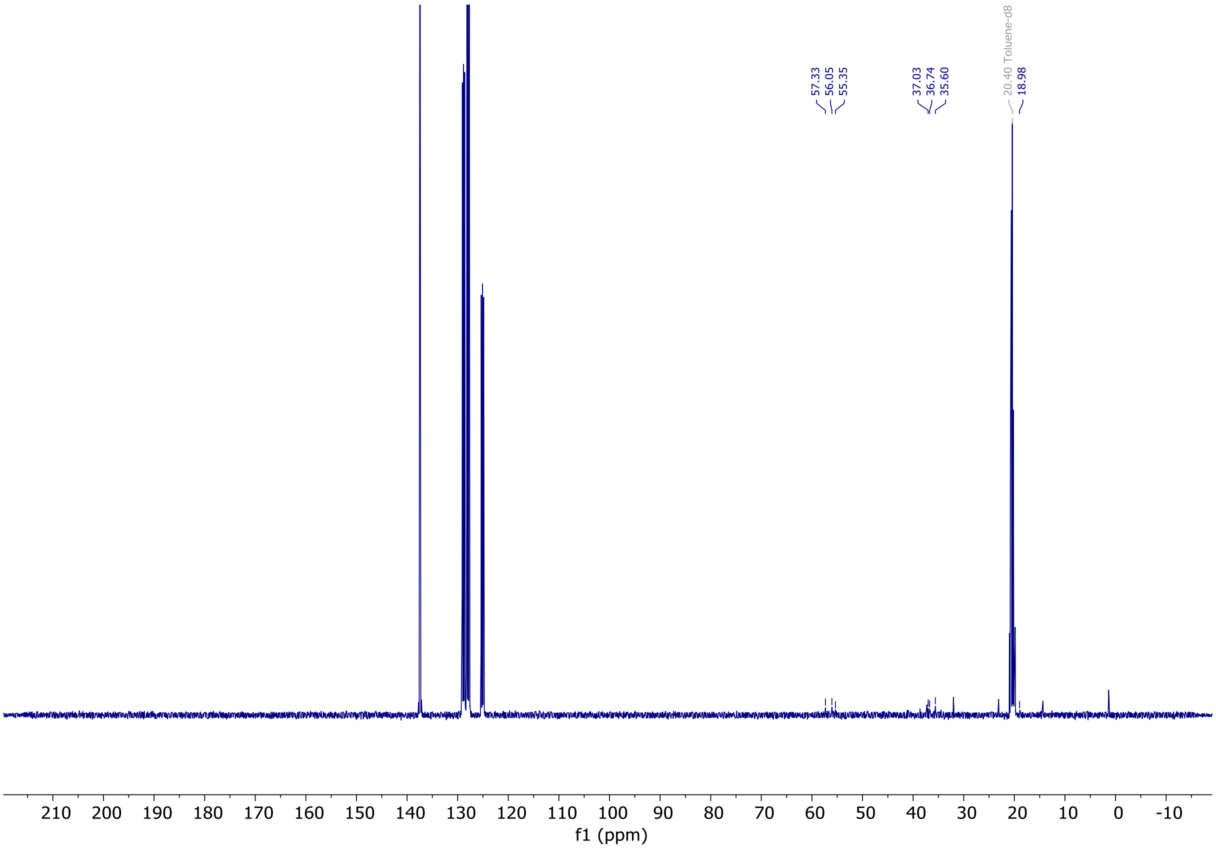

Figure S14 ^{13}C NMR spectrum of **1** in toluene-d_8 (full spectrum).

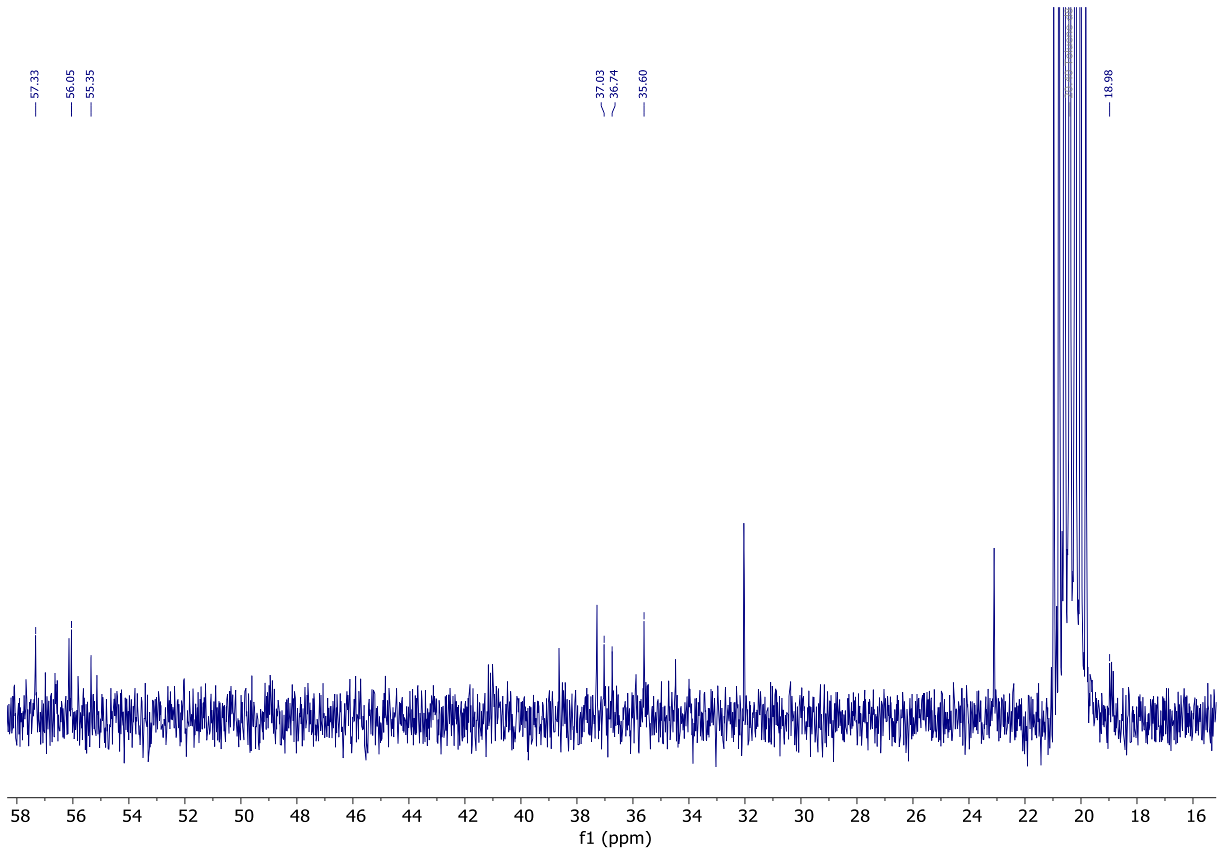

Figure S15 ^{13}C NMR spectrum of **1** in toluene-d_8 (close-up spectrum).

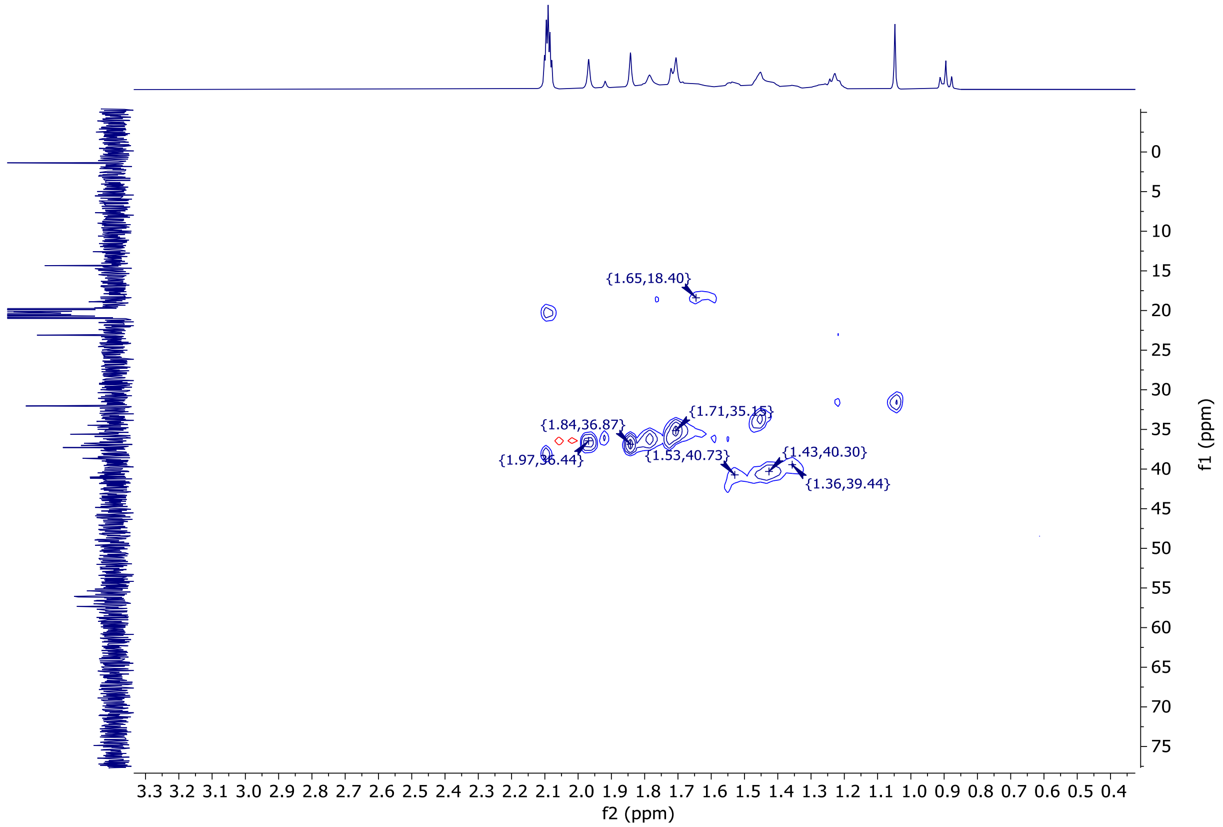

Figure S16 ^{1}H ^{13}C HSQC NMR spectrum of **1** in toluene-d_8.

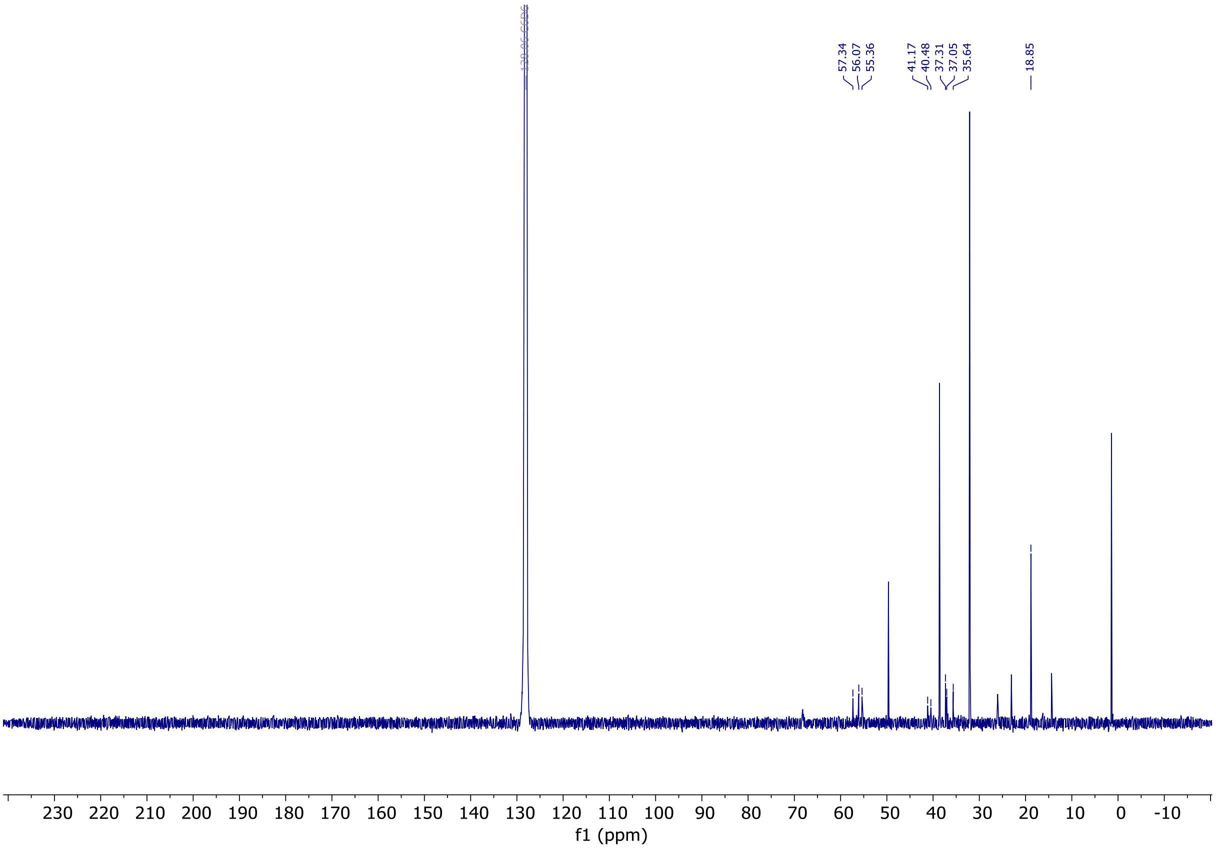

Figure S17 ^{13}C NMR spectrum of **1** in benzene-d_6 (full spectrum).

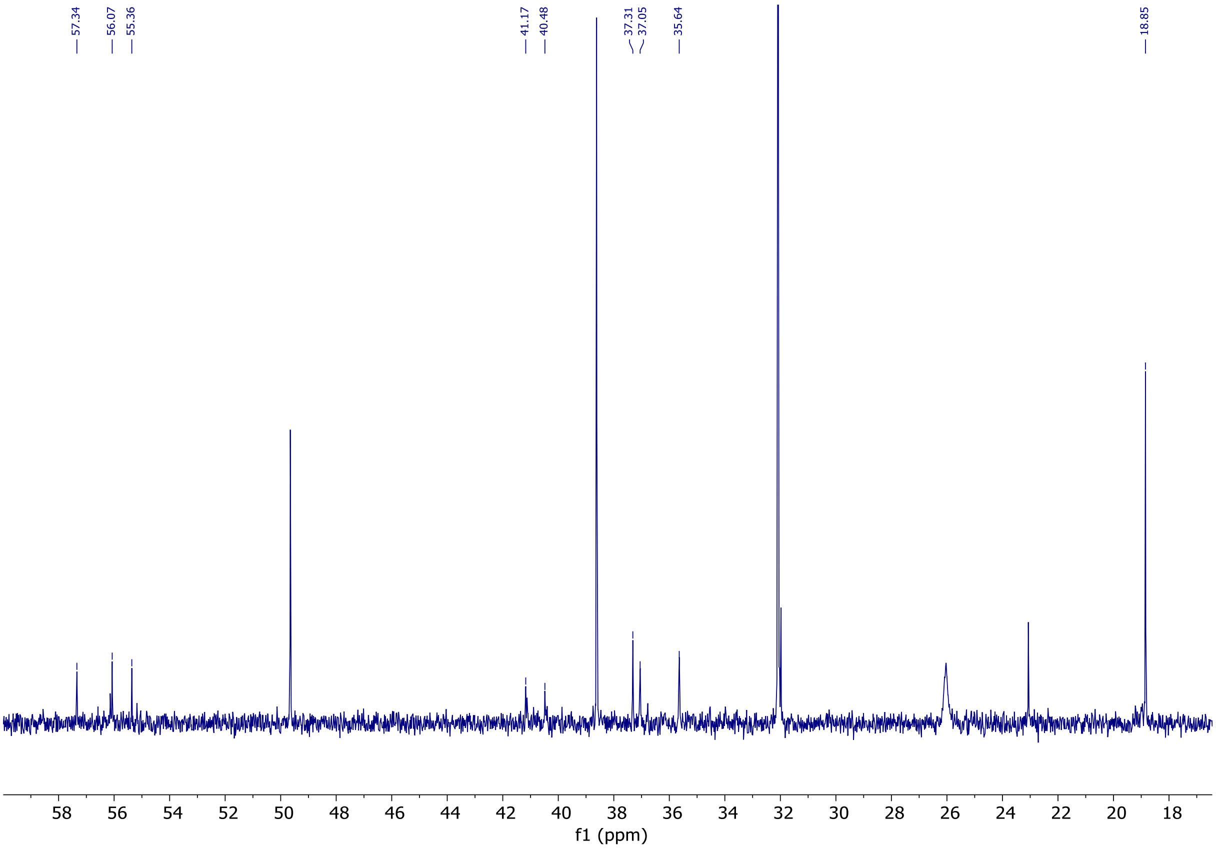

Figure S18 ^{13}C NMR spectrum of **1** in benzene-d_6 (close-up spectrum).

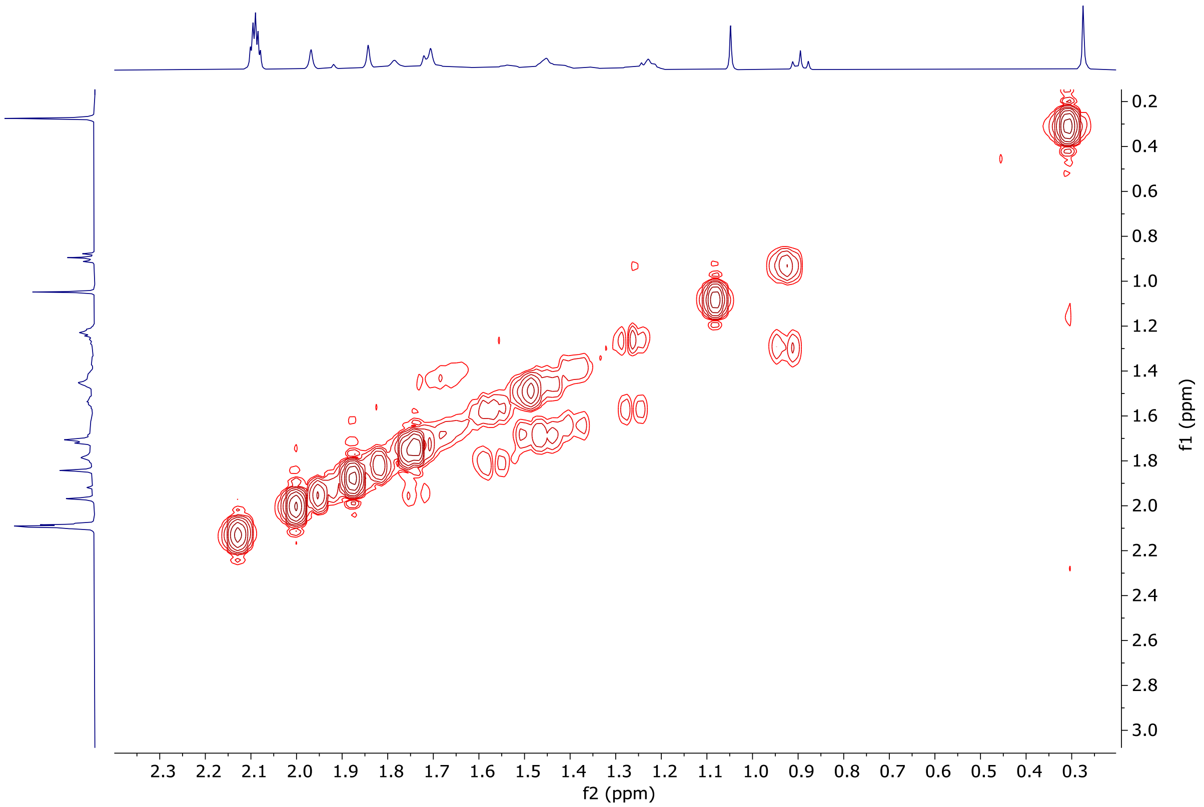

Figure S19 ^1H ^1H COSY NMR spectrum of **1** in toluene-d_8.

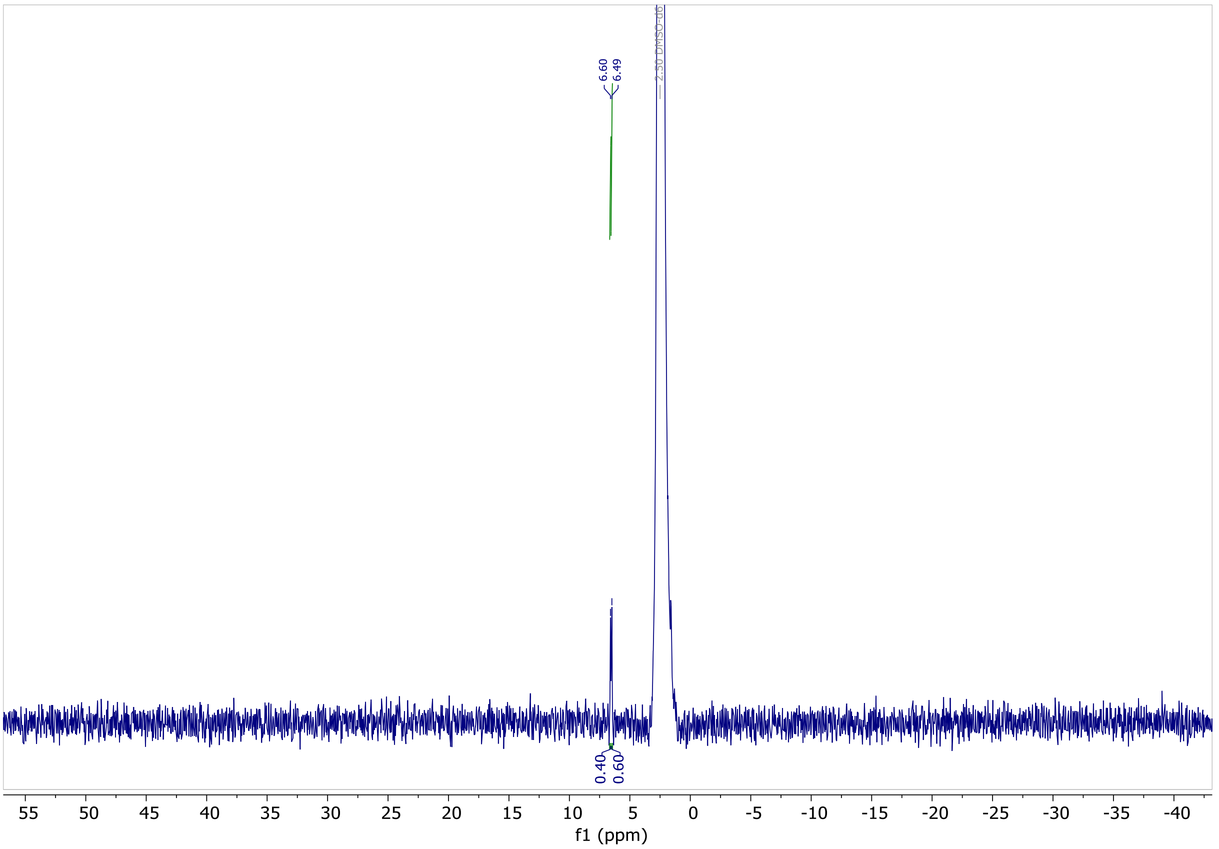

Figure S20 ^2H NMR spectrum of **1-D** in toluene and a capillary of DMSO-d_6 (full spectrum).

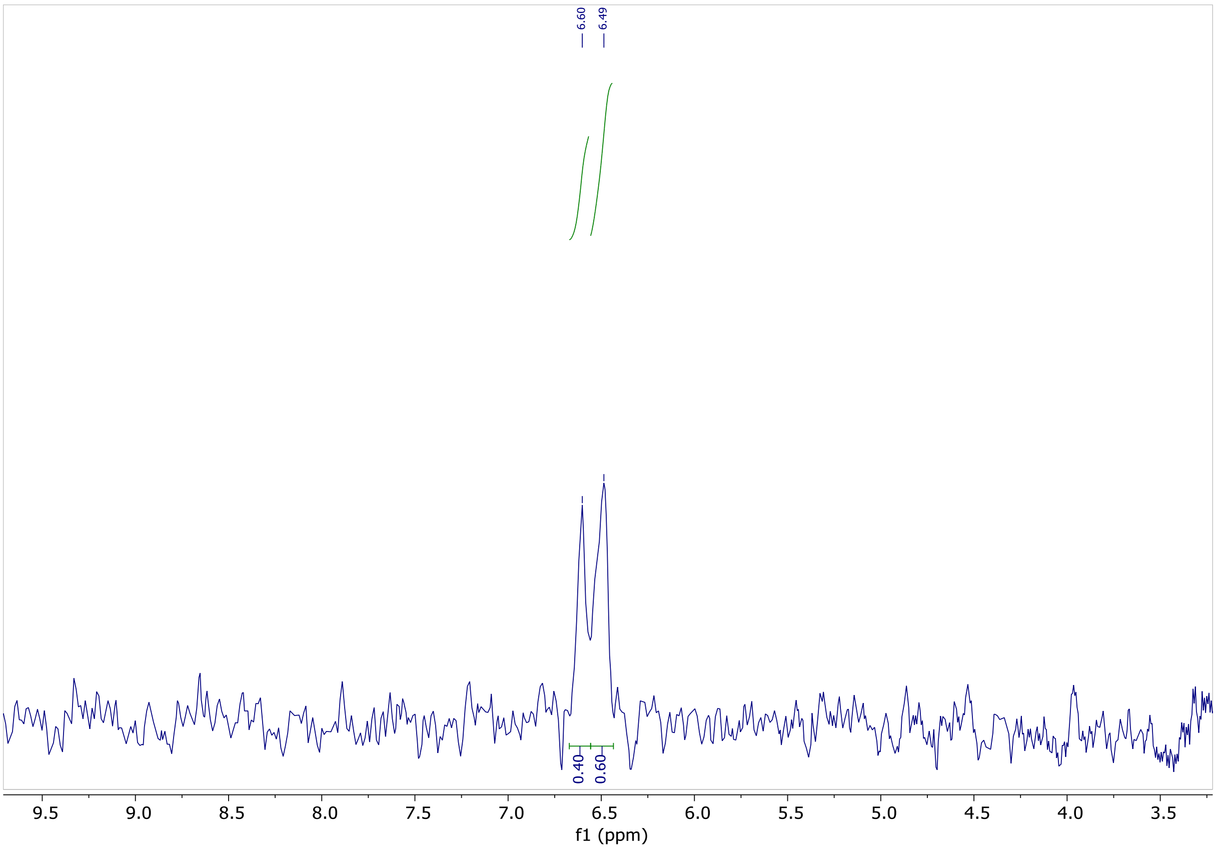

Figure S21 ^{2}H NMR spectrum of **1-D** in toluene and a capillary of DMSO-d_6 (close-up spectrum).

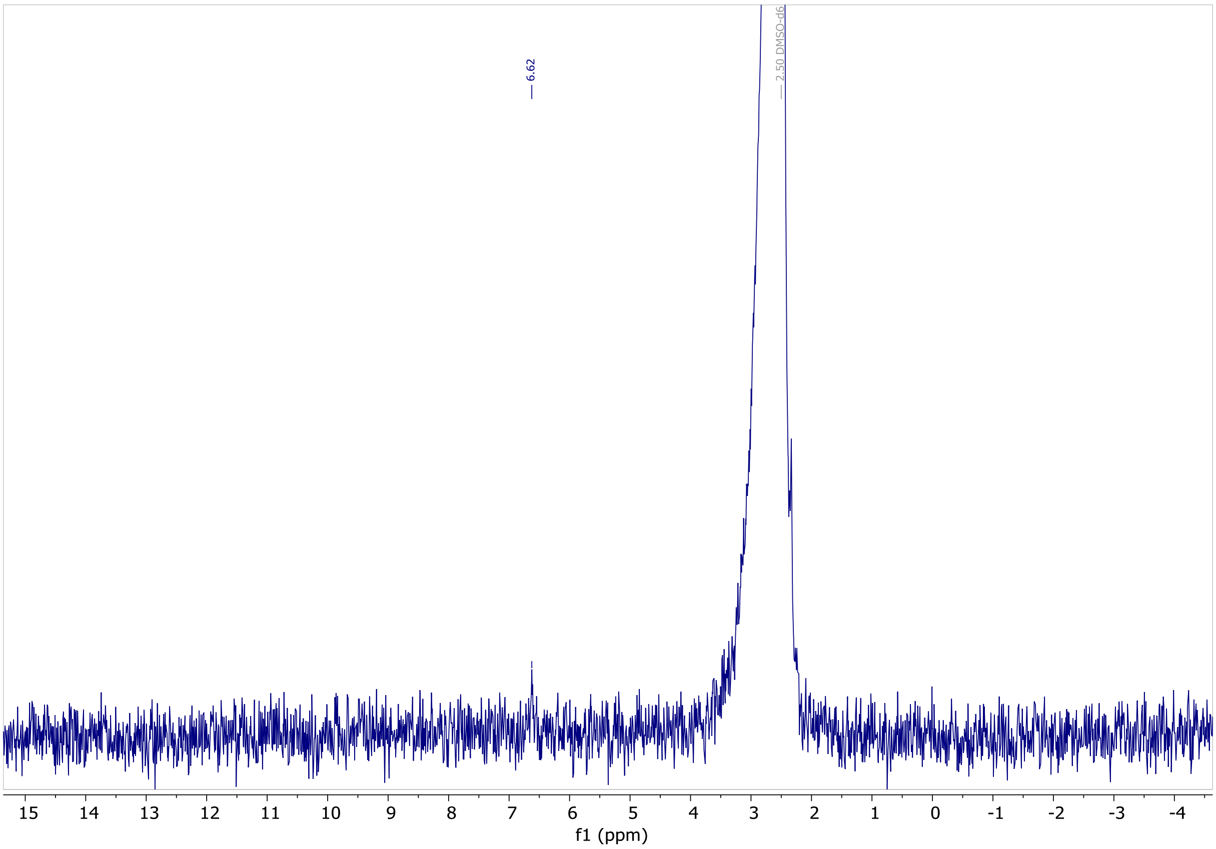

Figure S22 ^{2}H NMR spectrum of **1-^{71}Ga-D** prepared with isotopically labeled ^{71}GaTMP in benzene and a capillary of DMSO-d_6, showing only the signal allocated to the ^{71}GaD (see extended discussion to **1**).

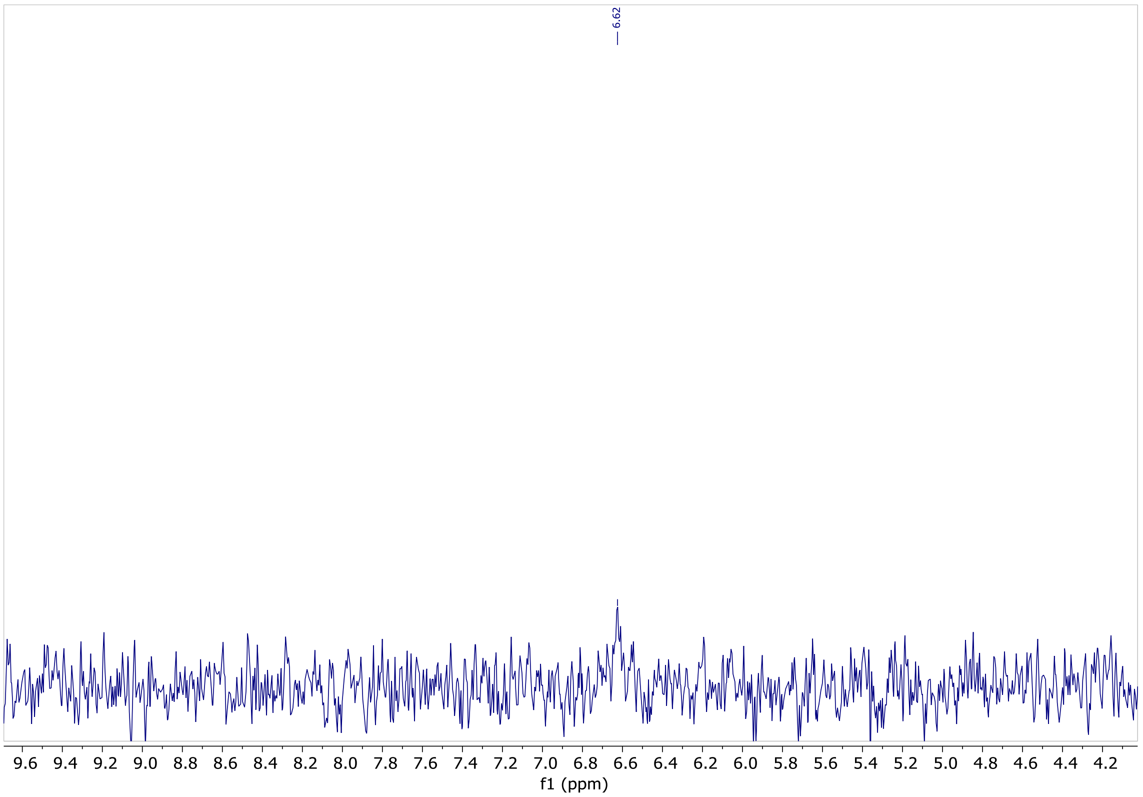

Figure S23 ^{2}H NMR spectrum of **1-^{71}Ga-D** prepared with isotopically labeled ^{71}GaTMP in benzene and a capillary of DMSO-d_6 (close-up spectrum), showing only the signal allocated to the ^{71}GaD (see extended discussion to **1**).

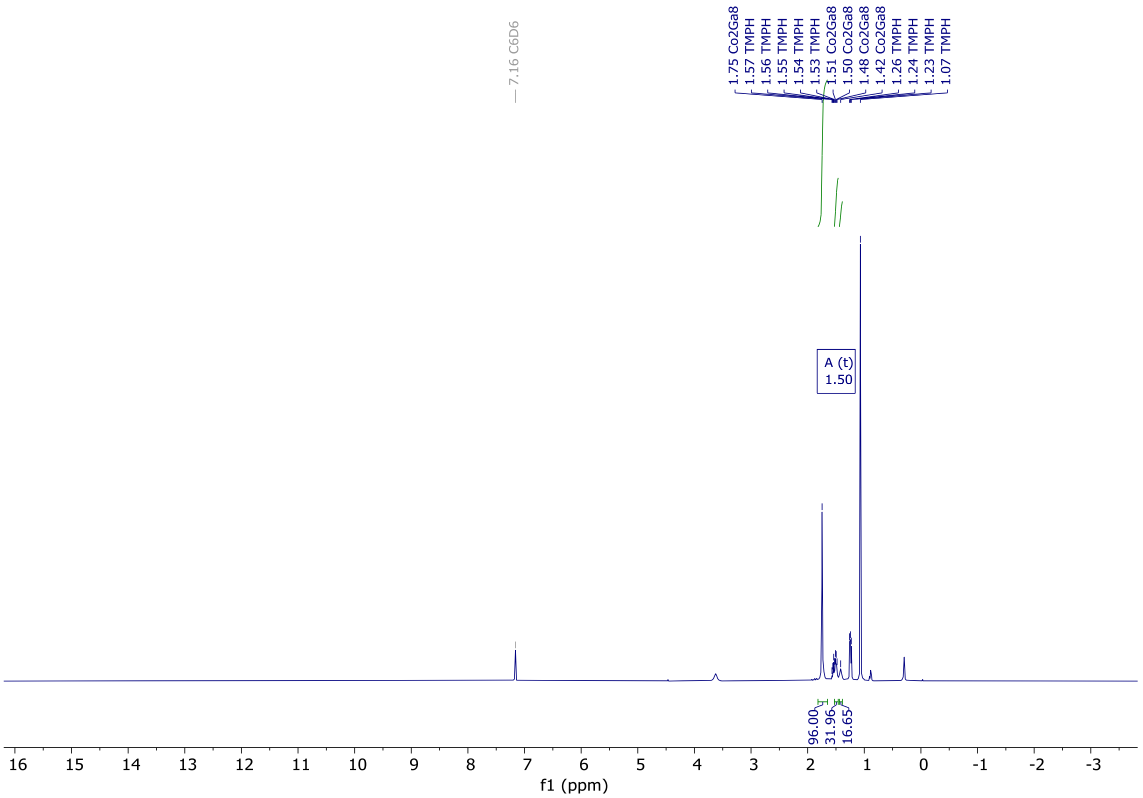

Figure S24 ^{1}H NMR spectrum of **3** in benzene-d_6 (full range; detected impurities are TMPH due to decomposition of small amounts of the compound upon contact with the solvent, hexane/THF as crystallization/reaction medium and grease, see also Figure S25 below).

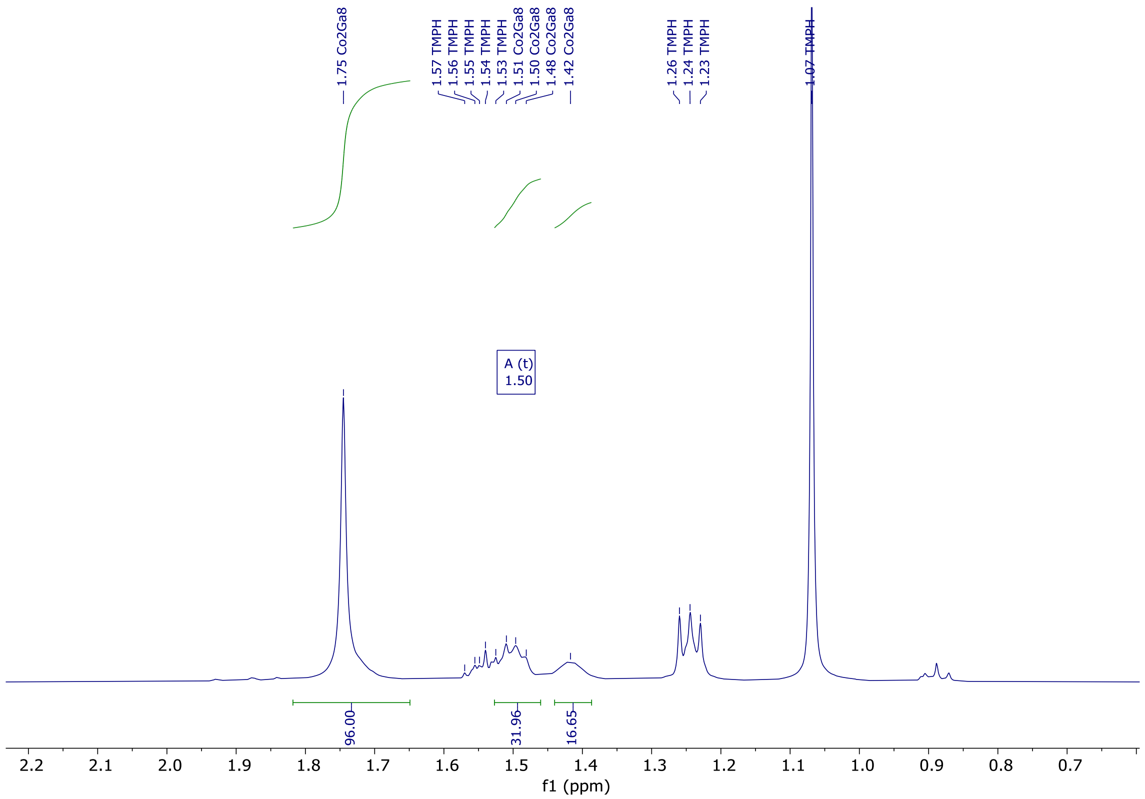

Figure S25 ^{1}H NMR spectrum of **3** in benzene-d_6 (close-up spectrum; detected impurities are TMPH due to decomposition of small amounts of the compound upon contact with the solvent, hexane/THF as crystallization/reaction medium and grease, see also Figure S24 above).

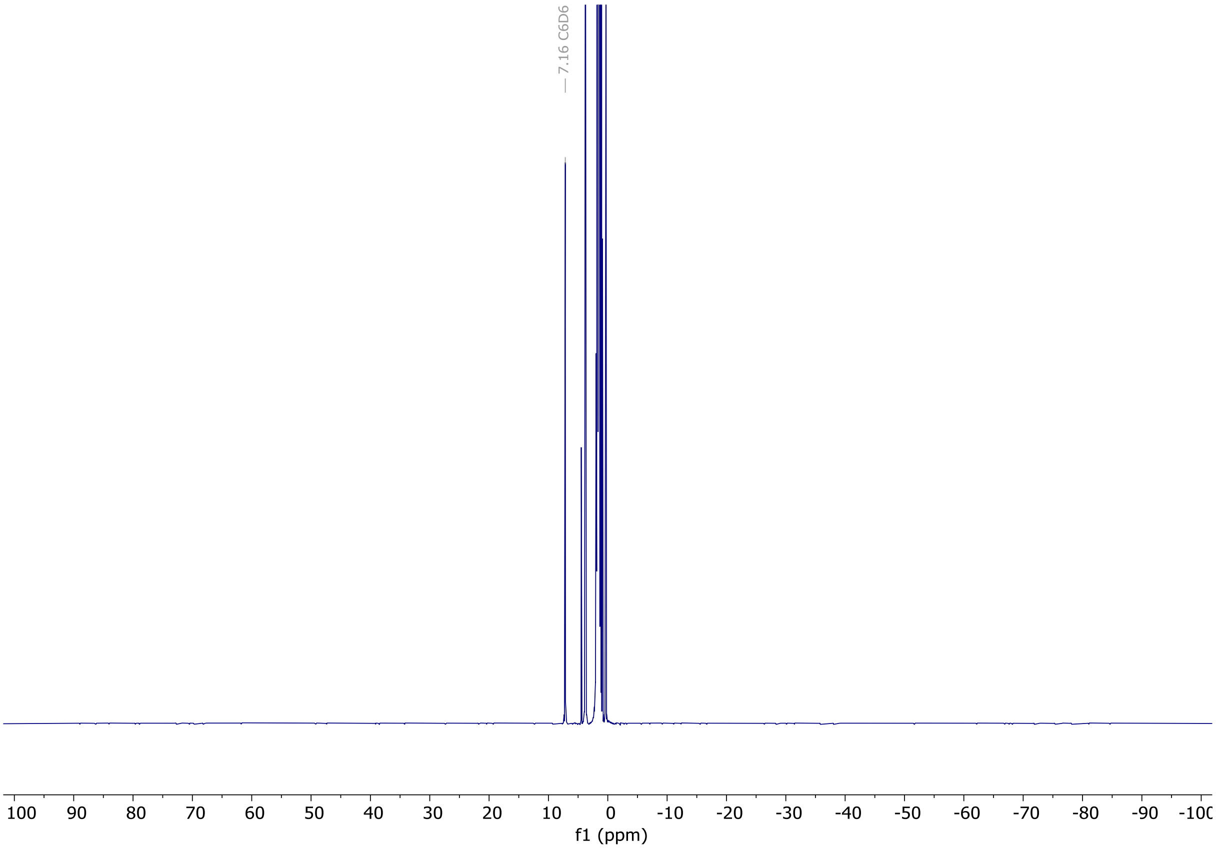

Figure S26 ^{1}H NMR spectrum (paramagnetic) of **3** in benzene-d_6.

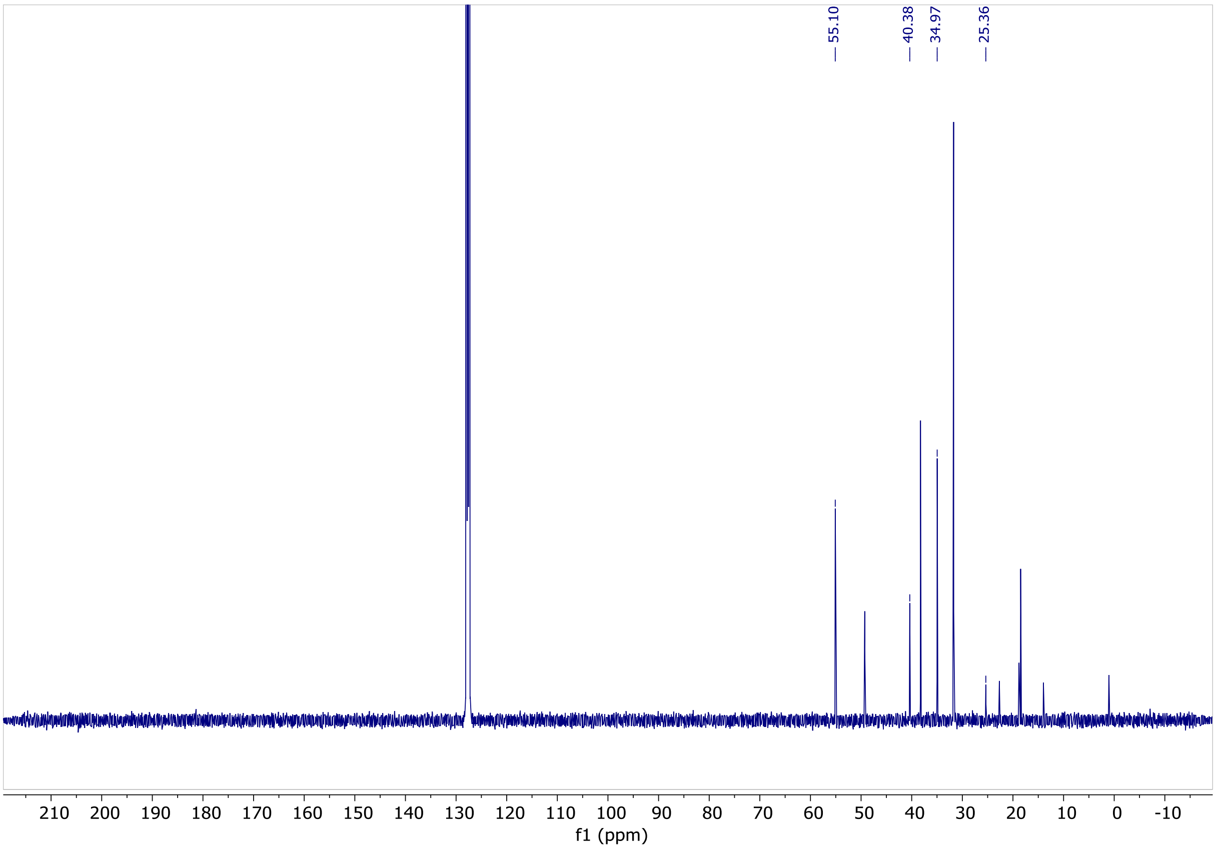

Figure S27 ^{13}C NMR spectrum of **3** in benzene-d_6 (full spectrum).

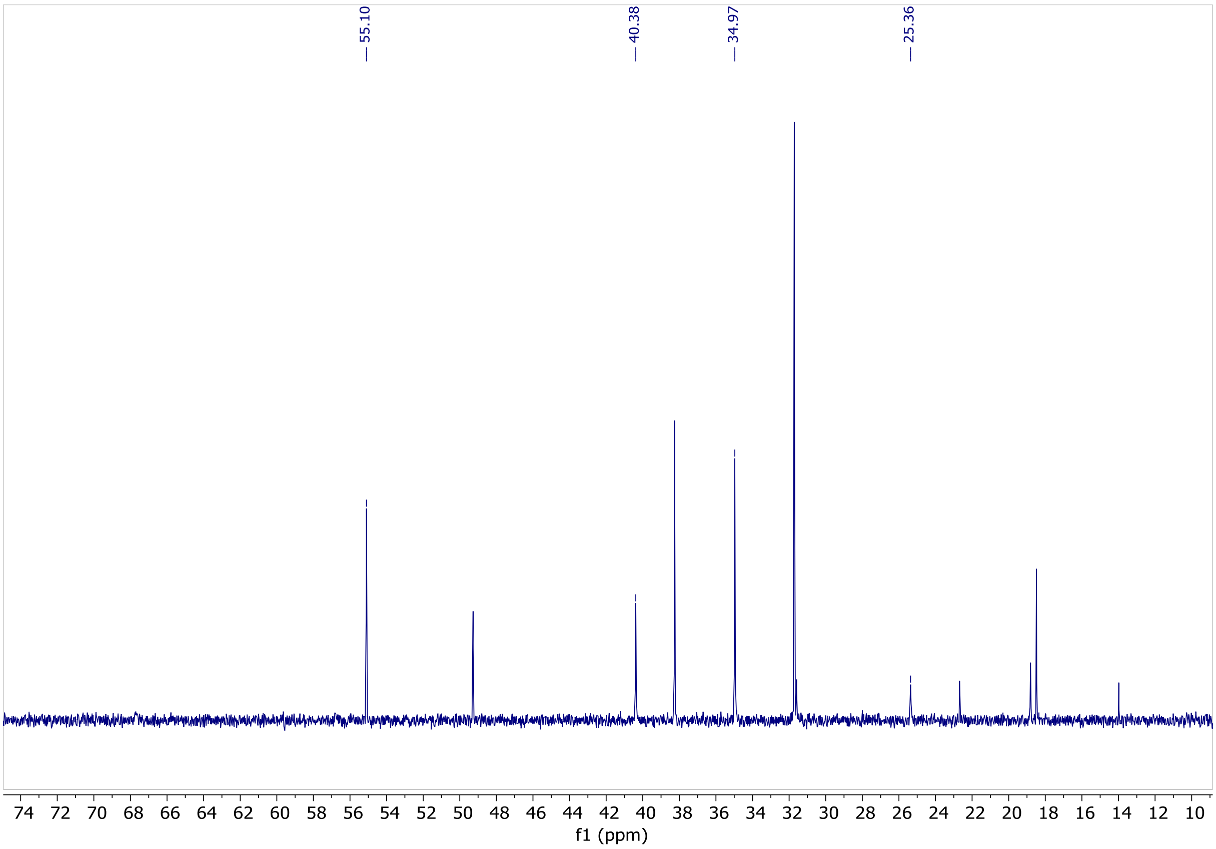

Figure S28 ^{13}C NMR spectrum of **3** in benzene-d_6 (close-up spectrum).

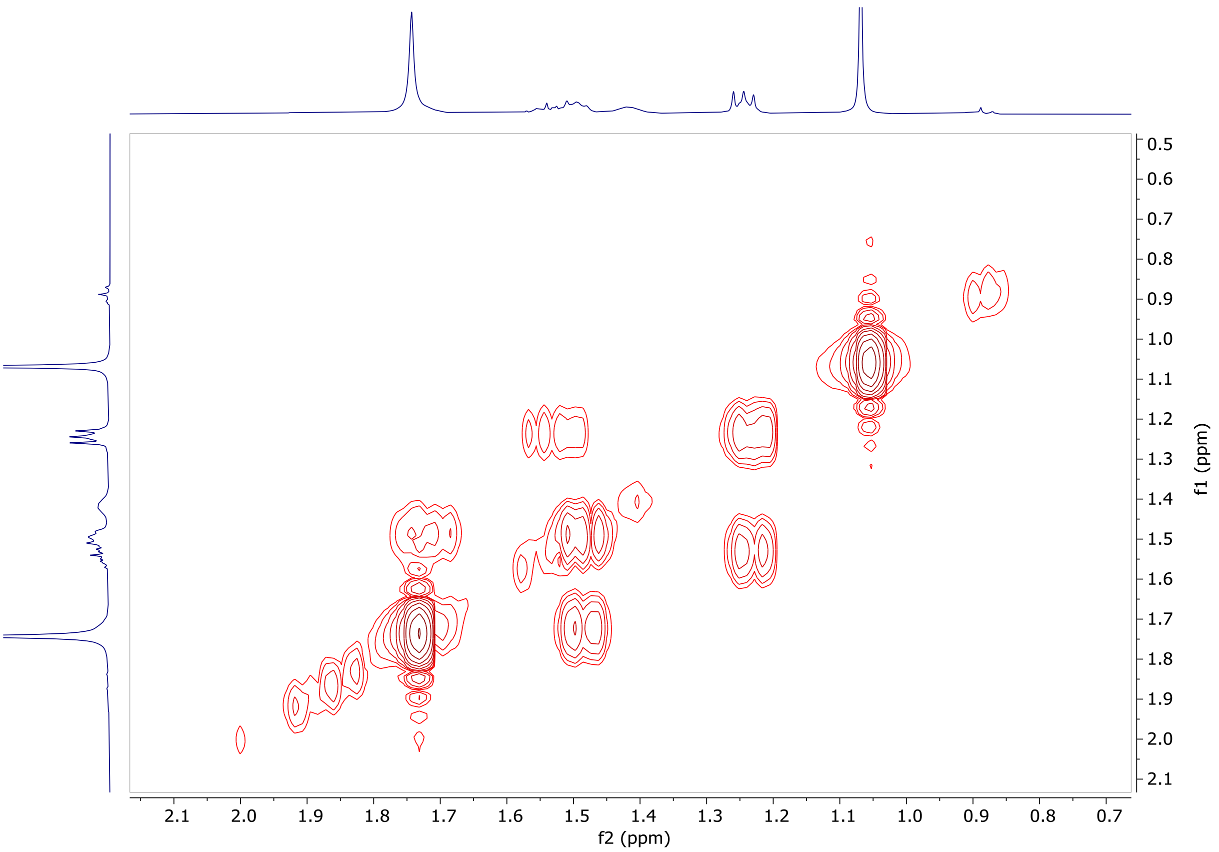

Figure S29 ^{1}H ^{1}H COSY NMR spectrum of **3** in benzene-d_6.

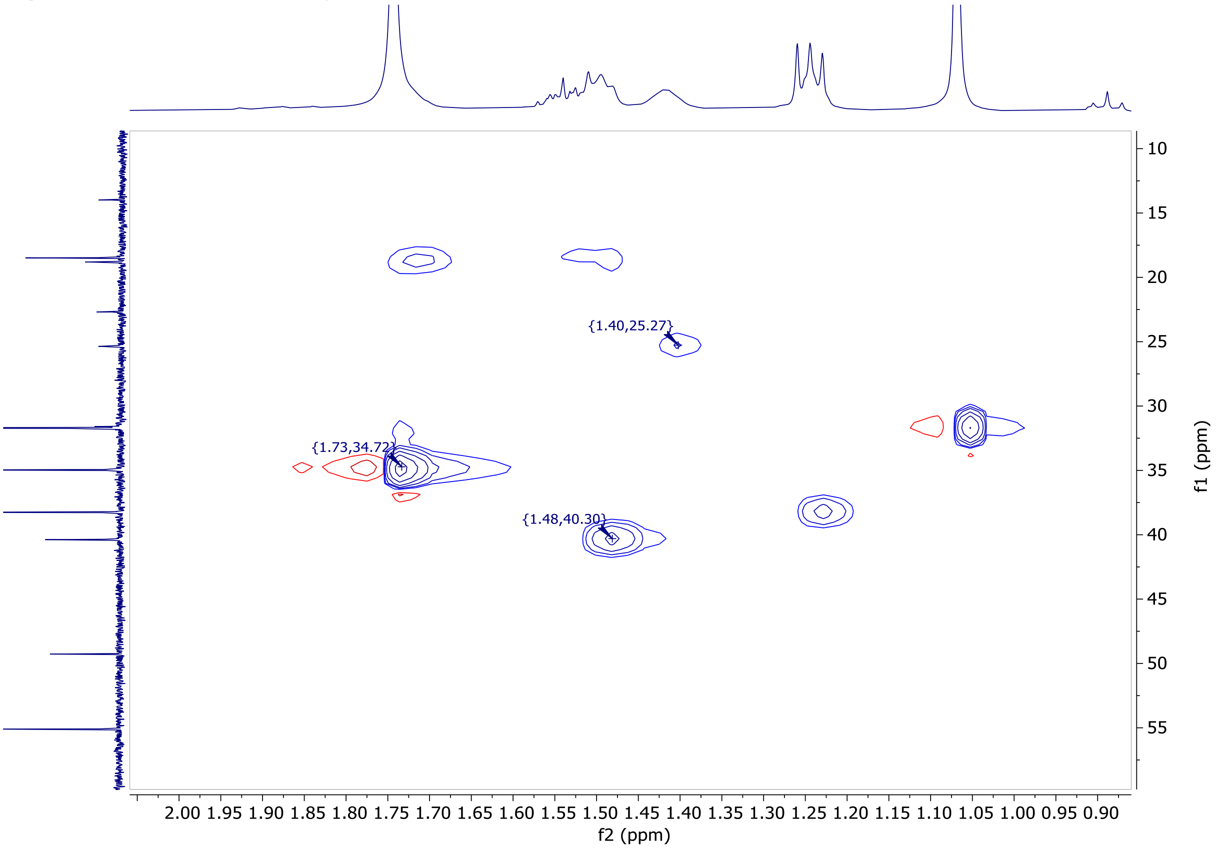

Figure S30 ^{1}H ^{13}C HSQC NMR spectrum of **3** in benzene-d_6.

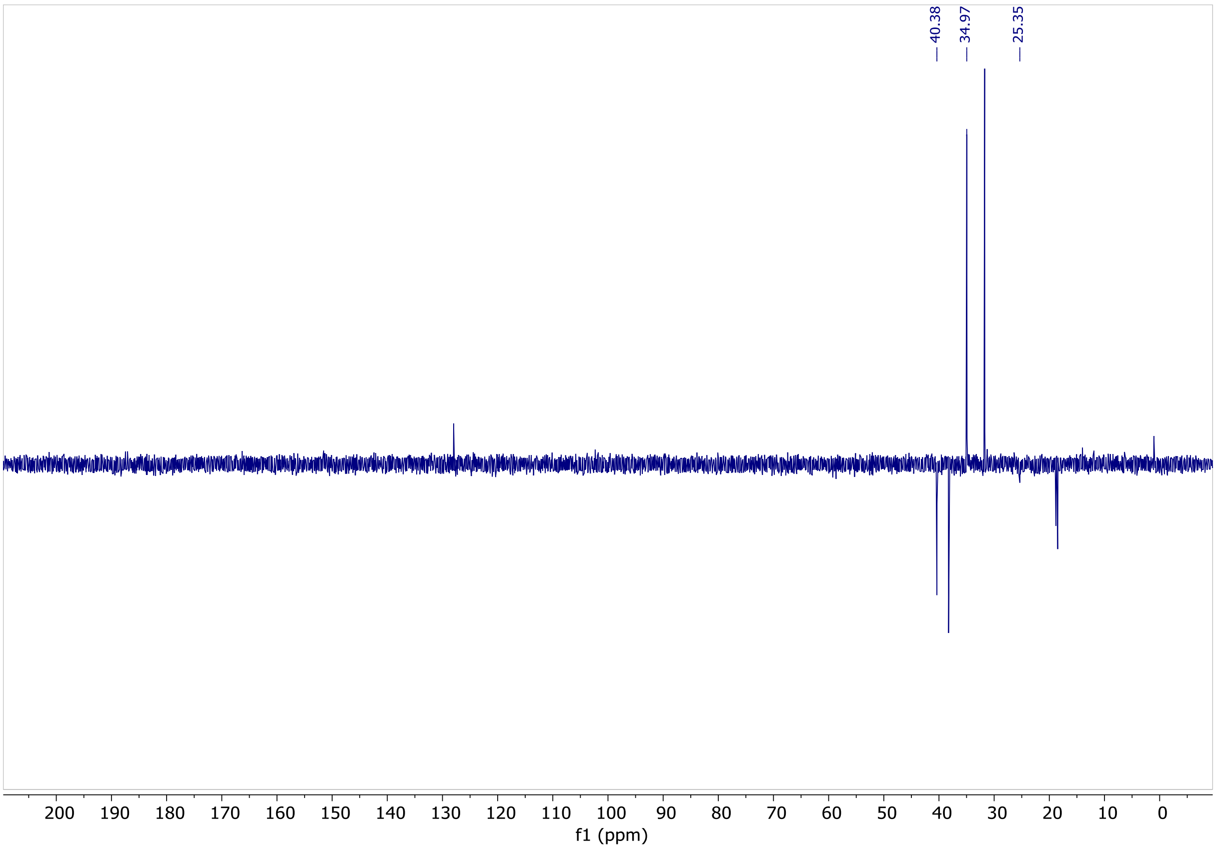

Figure S31 DEPT-135 NMR spectrum of **3** in benzene-d_6 (full spectrum)

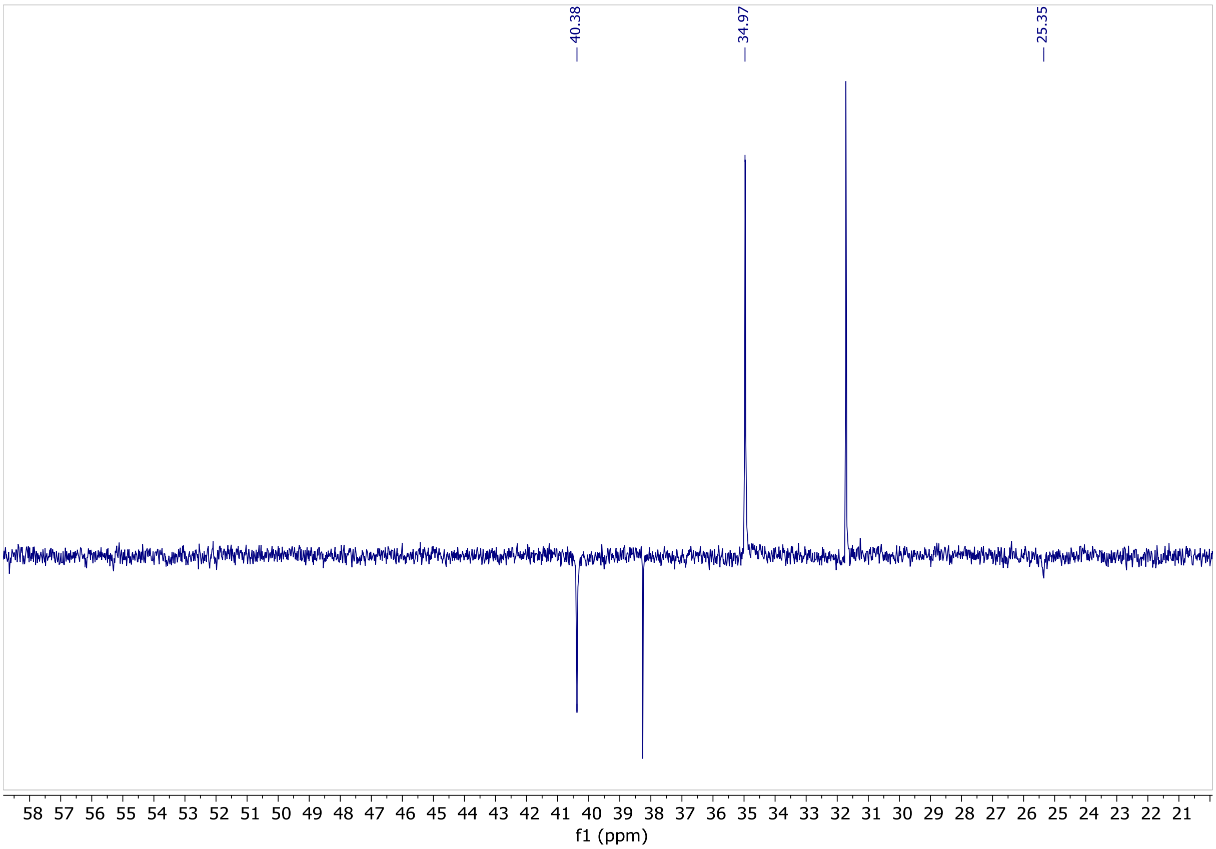

Figure S32 DEPT-135 NMR spectrum of **3** in benzene-d_6 (close-up spectrum).

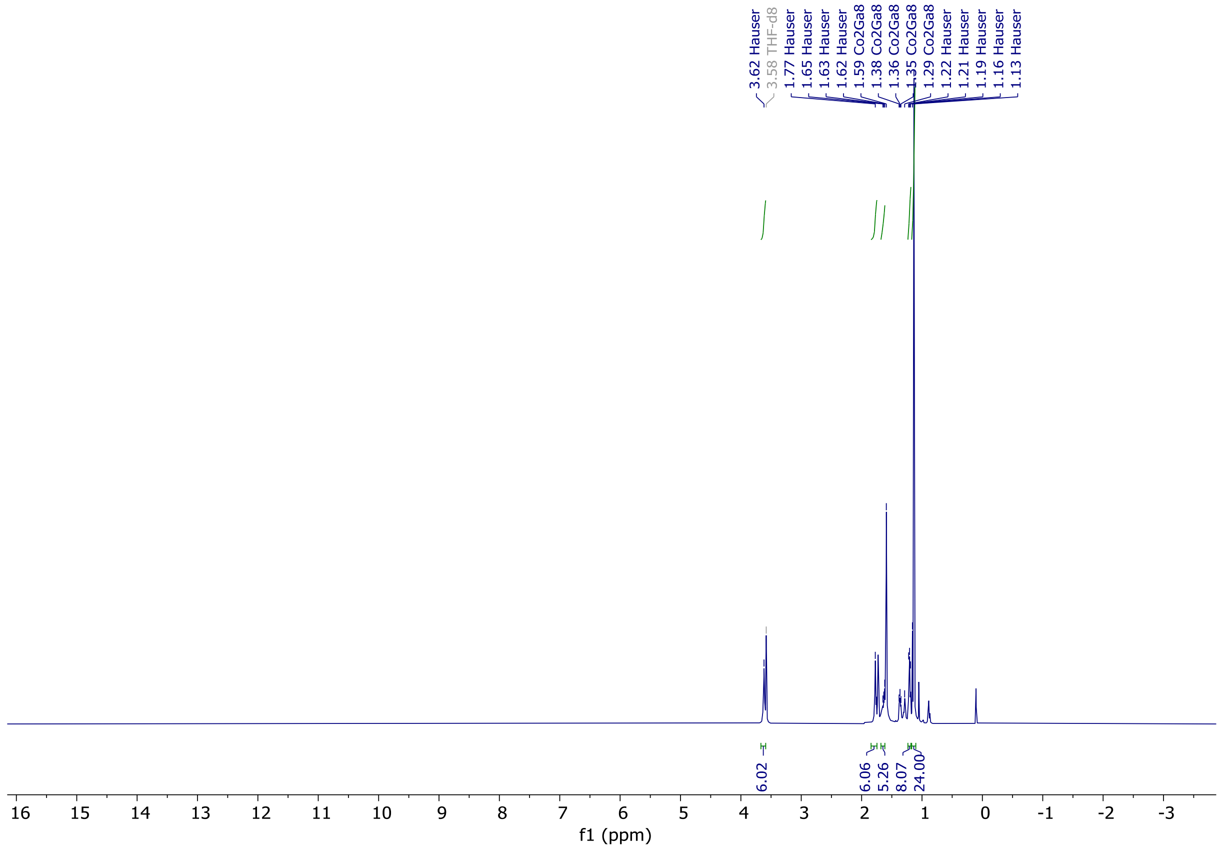

Figure S33 ^{1}H NMR spectrum of the *Hauser*-base $[Mg(TMP)(THF)\mu^{2}\text{-}Cl]_2$ and **3** in THF-d_8 (full spectrum).

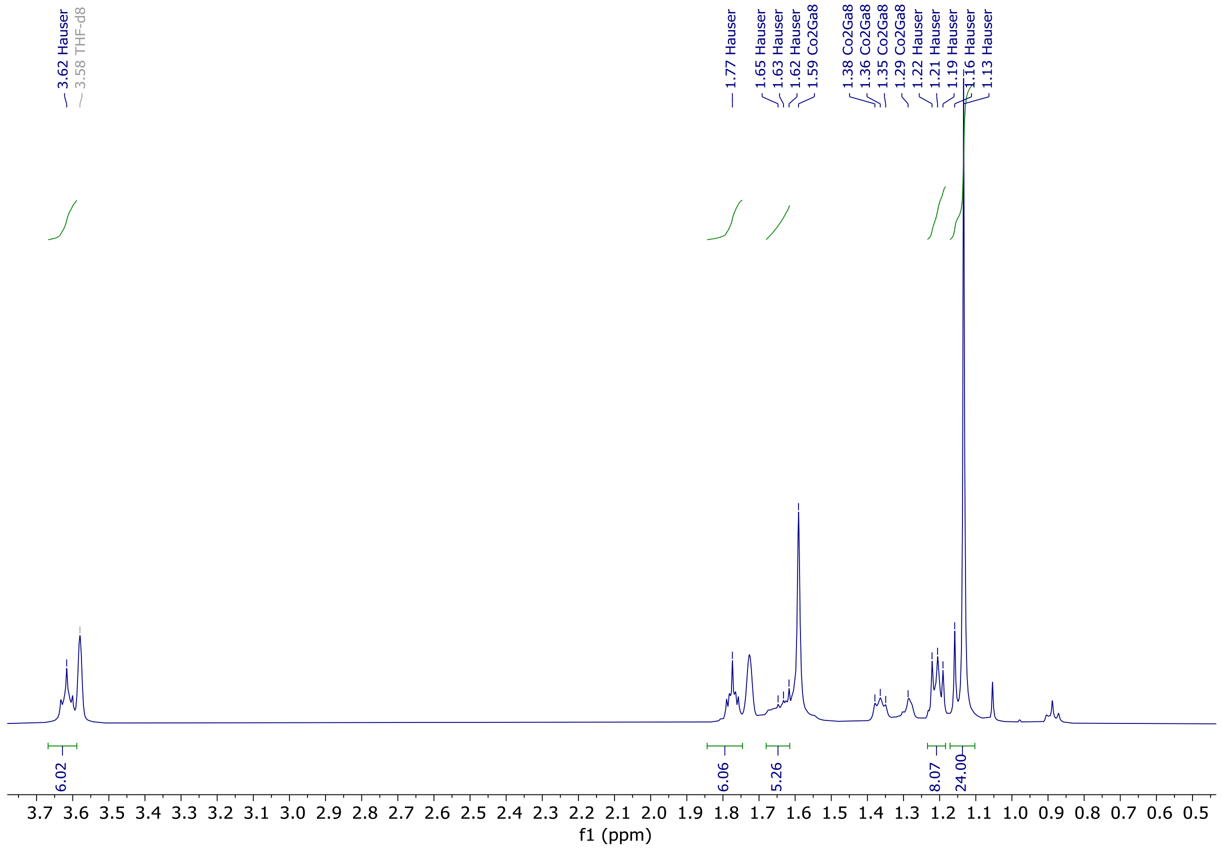

Figure S34 ^{1}H NMR spectrum of the *Hauser*-base $[Mg(TMP)(THF)\mu^{2}\text{-}Cl]_2$ and **3** in THF-d_8 (close-up spectrum).

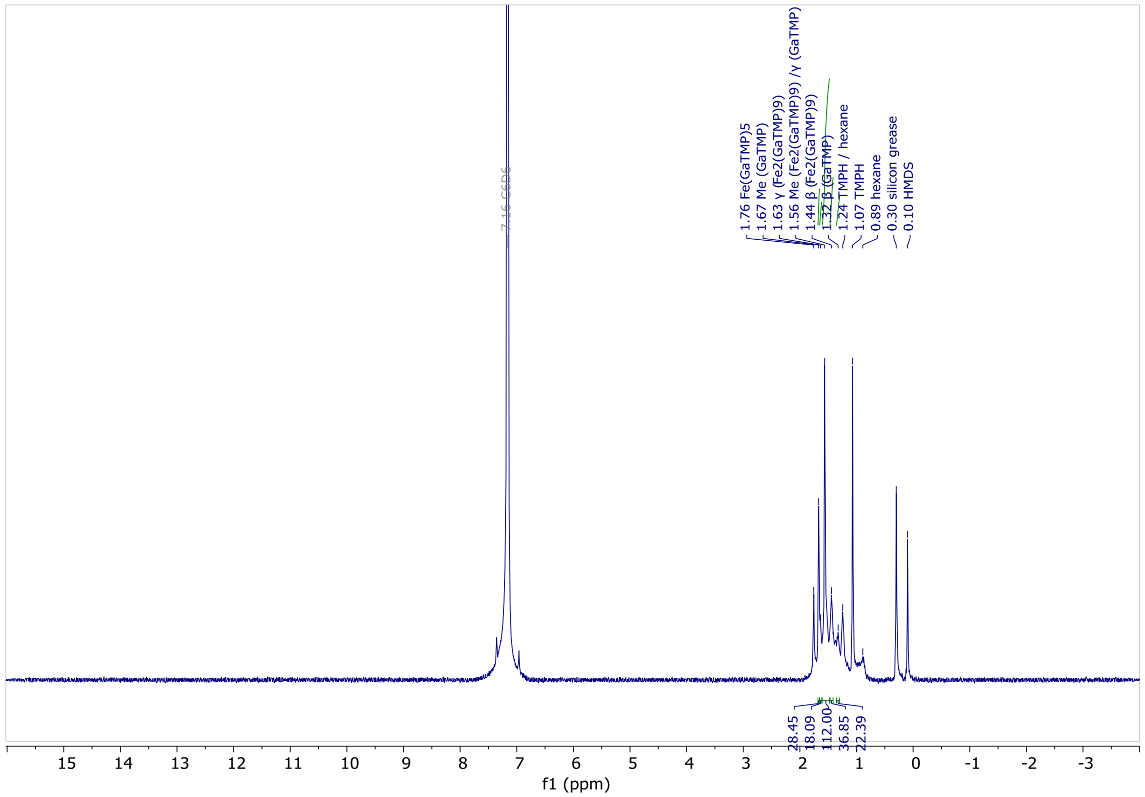

Figure S35 ^{1}H NMR spectrum of **4** in benzene-d_6, accompanied by Fe(GaTMP)$_5$ (side product) and GaTMP (substrate), beside impurities of silicon grease, hexane (crystallization) and HMDS (NMR tube inertization). Integral of the methyl group of the GaTMP ligand of **4** overestimated due to the overlap with the signal of the protons in γ-position of the uncoordinated GaTMP. See also Figure S36 and allocation in the spectrum.

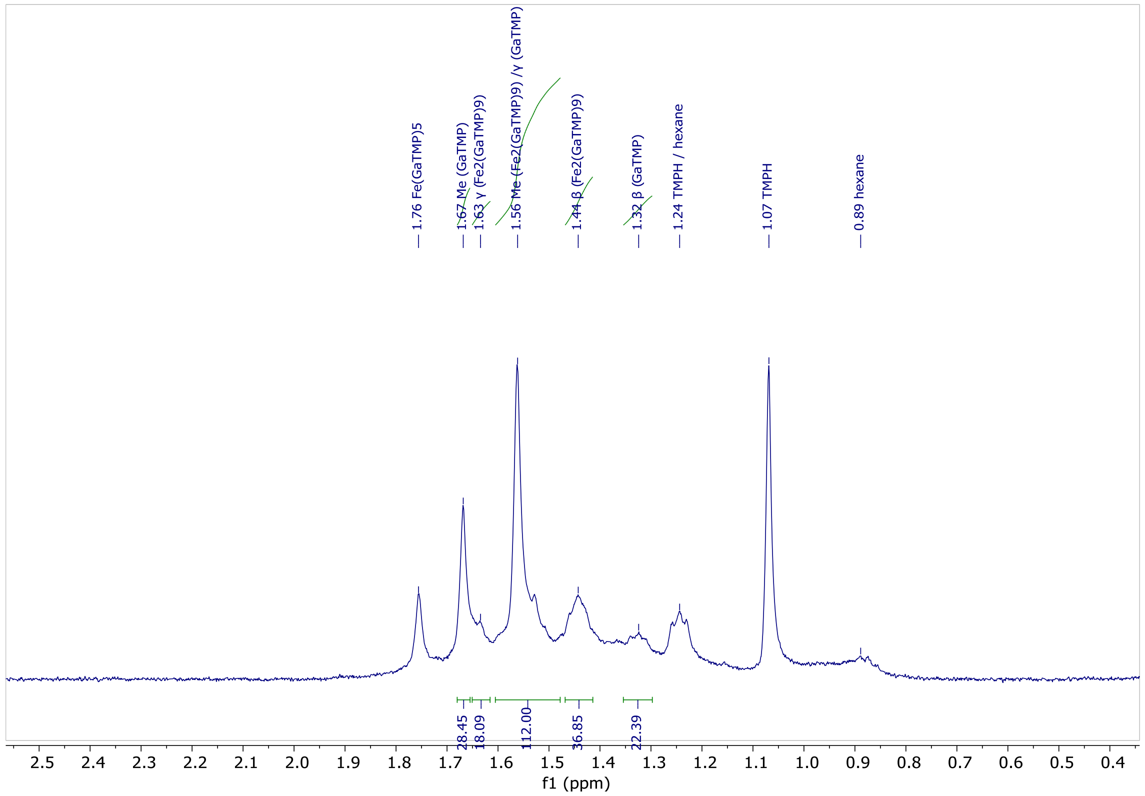

Figure S36 ^{1}H NMR spectrum of **4** in benzene-d_6 (close-up), accompanied by Fe(GaTMP)$_5$ (side product) and GaTMP (substrate), beside impurities of silicon grease, hexane (crystallization) and HMDS (NMR tube inertization). Integral of the methyl group of the GaTMP ligand of **4** overestimated due to the overlap with the signal of the protons in γ-position of the uncoordinated GaTMP. See also allocation in the spectrum.

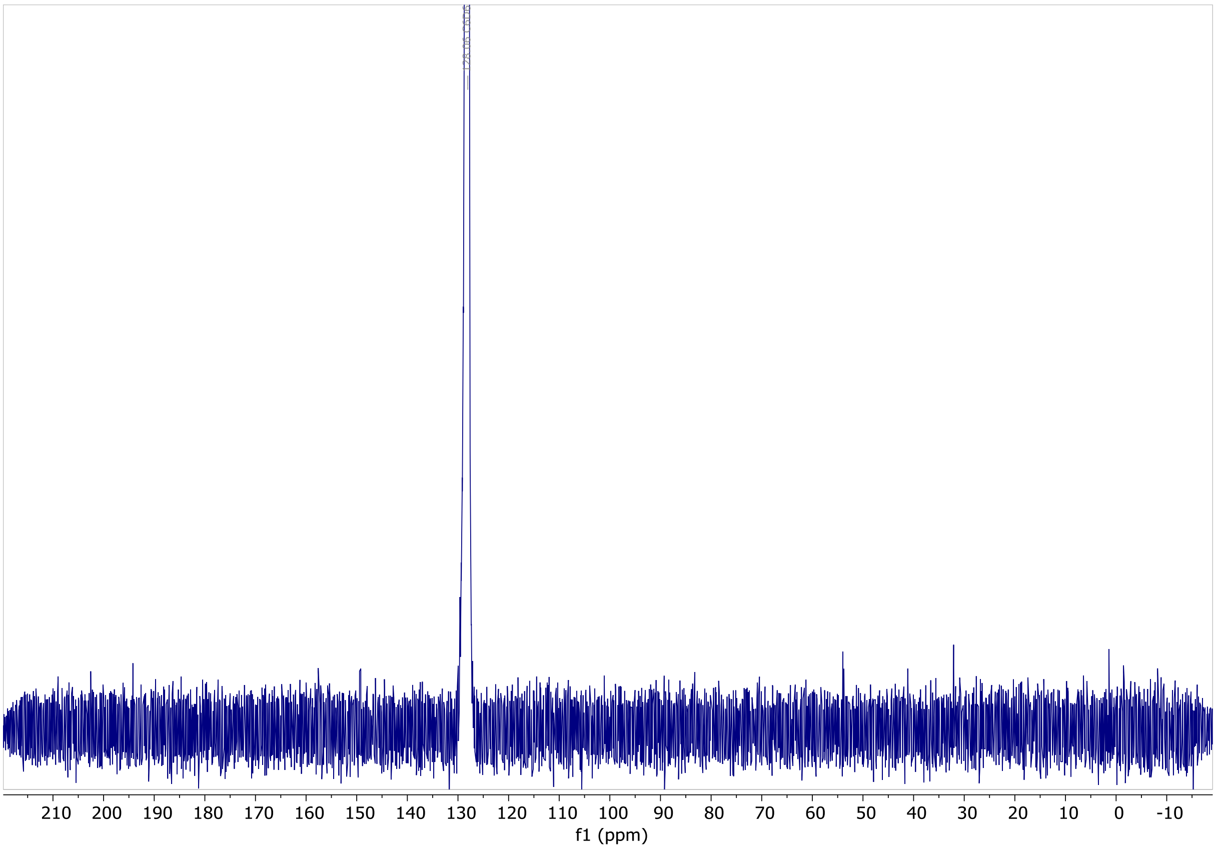

Figure S37 ^{13}C NMR spectrum of **4** in benzene-d_6. ^{13}C chemical shifts determined by HSQC and HMBC spectra (see Figures S39 and S40).

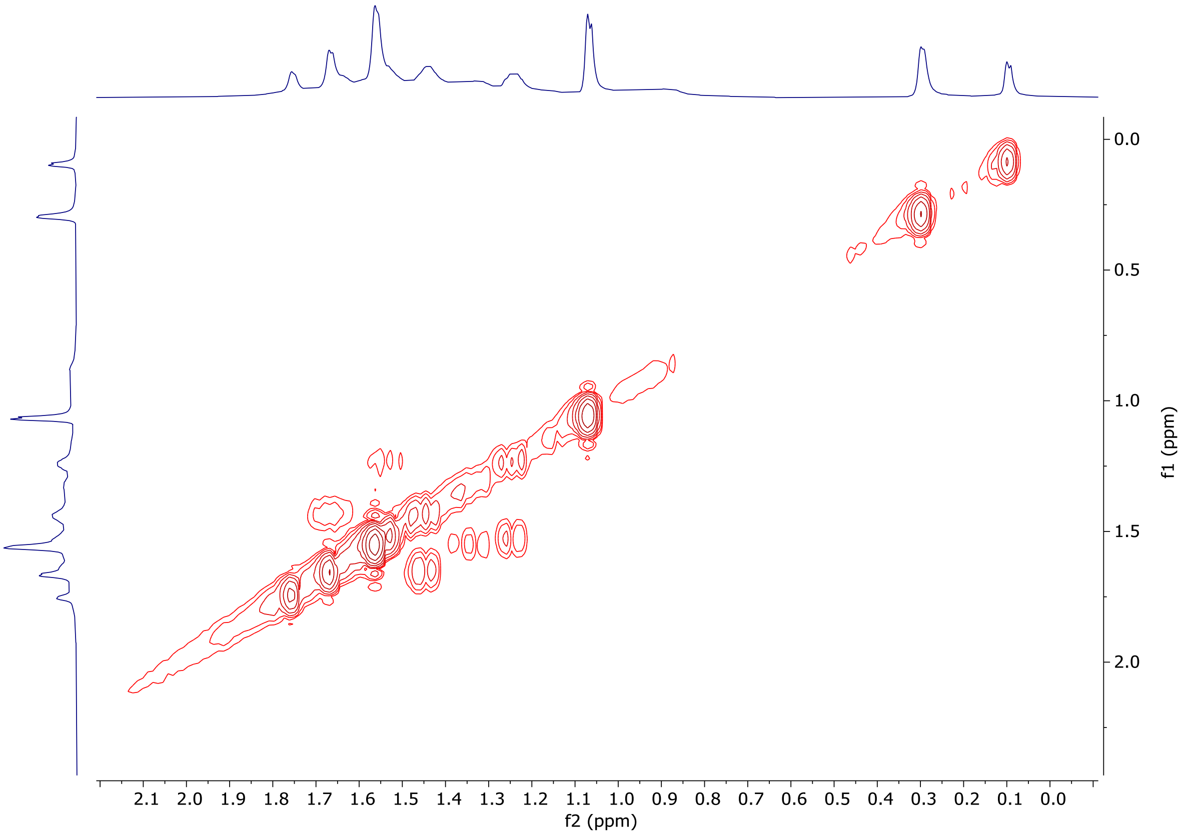

Figure S38 ^{1}H ^{1}H COSY NMR spectrum of **4** in benzene-d_6.

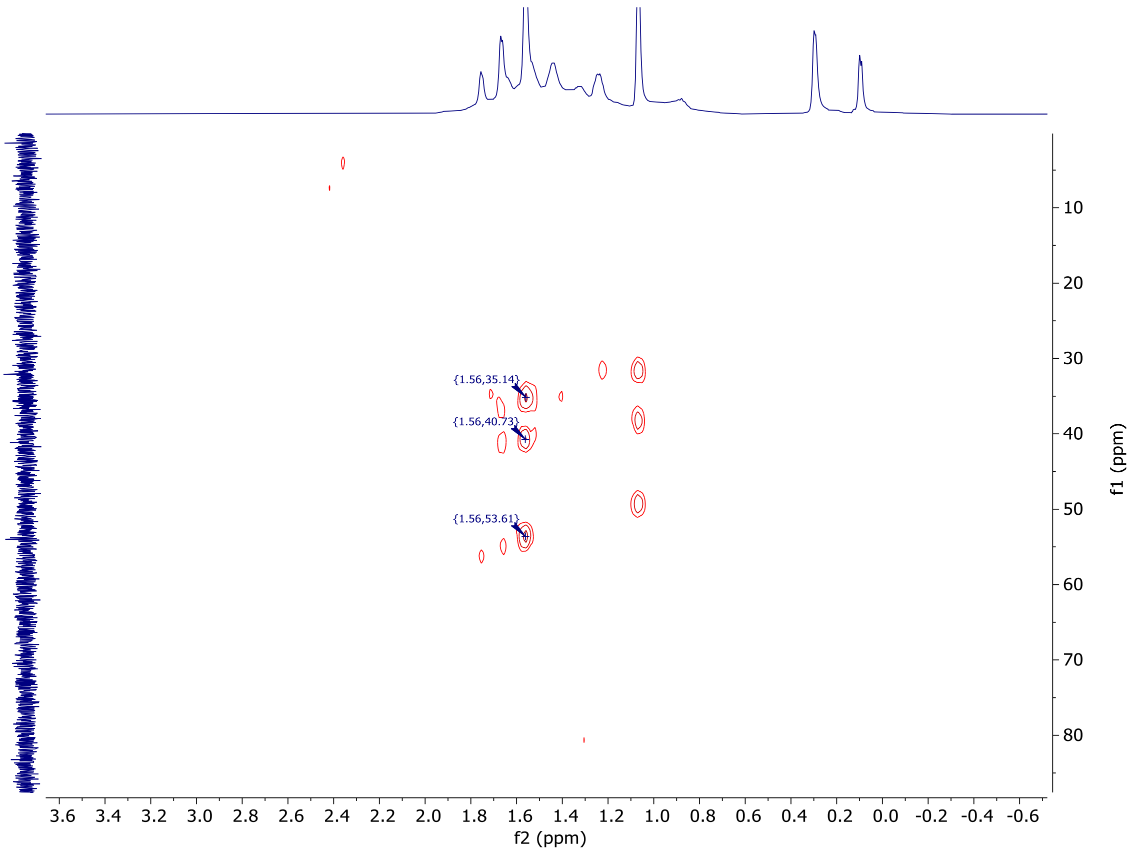

Figure S39 ^{1}H ^{13}C HMBC NMR spectrum of **4** in benzene-d_6.

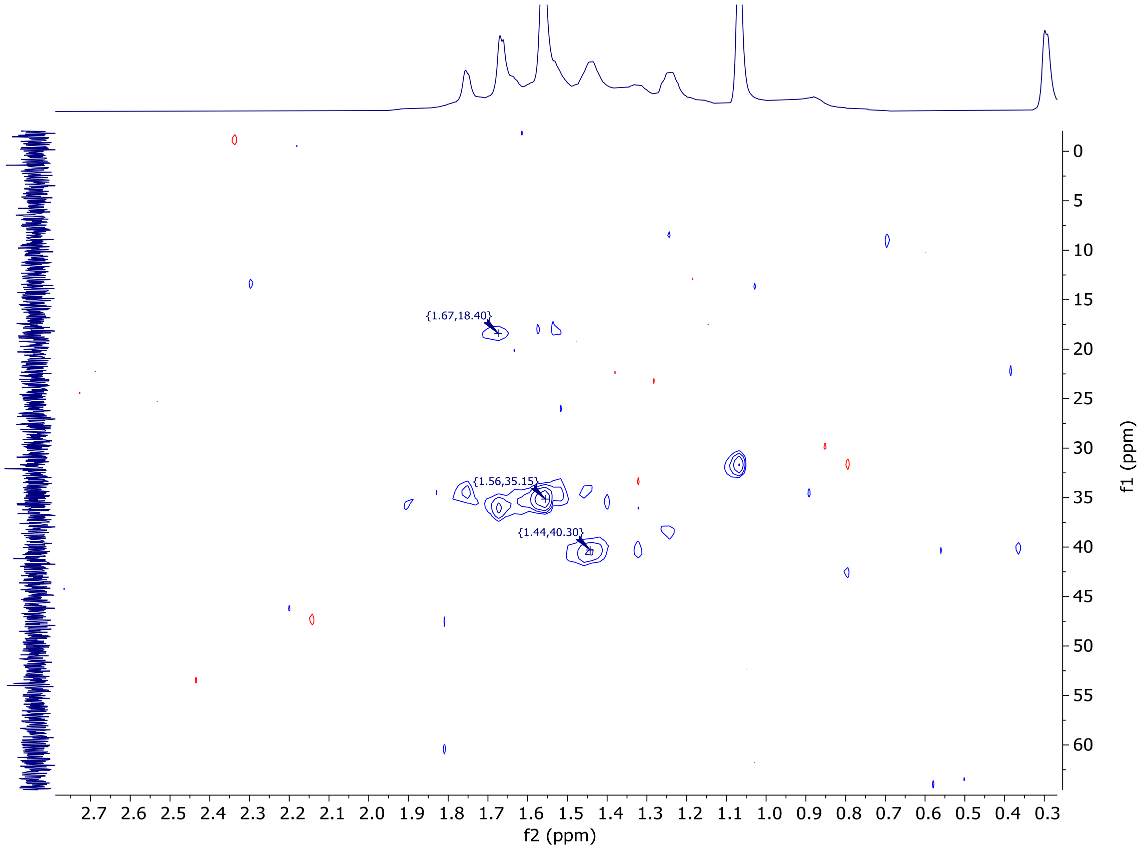

Figure S40 ^{1}H ^{13}C HSQC NMR spectrum of **4** in benzene-d_6.

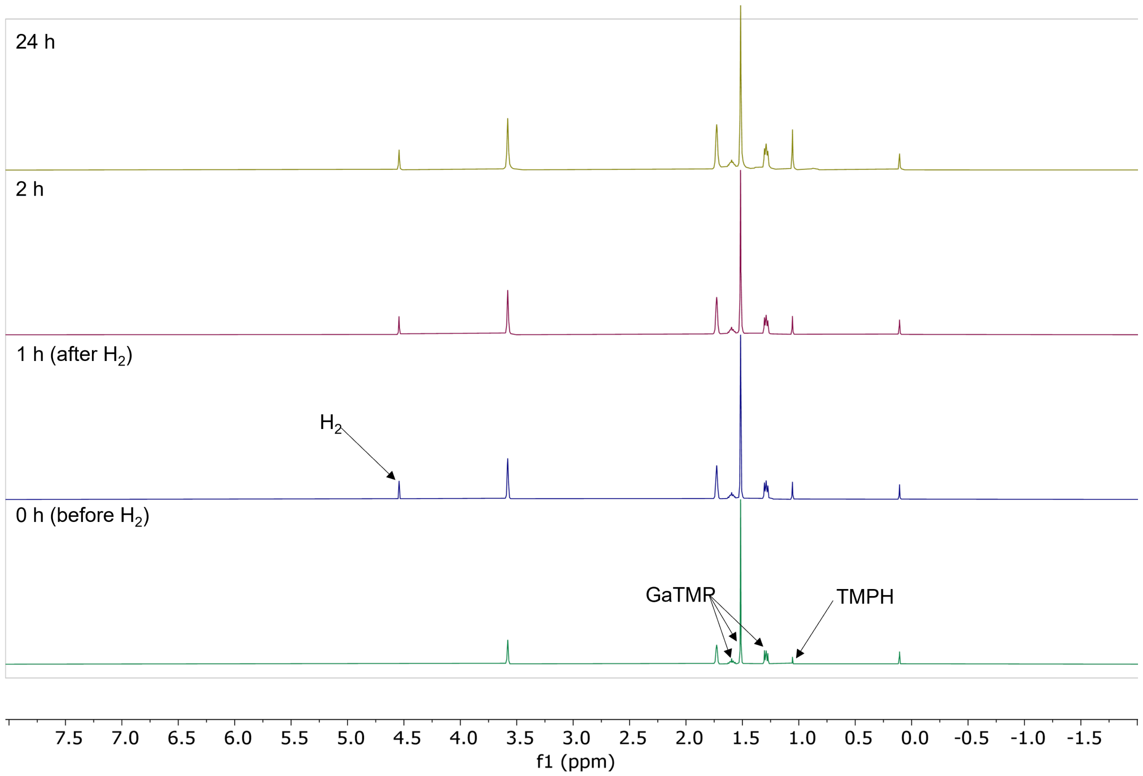

Figure S41 ^{1}H-NMR spectra of the reaction of GaTMP with 1 bar of H_2 over the course of 24 h in THF-d_8. No changes visible despite a small increase of TMPH over time.

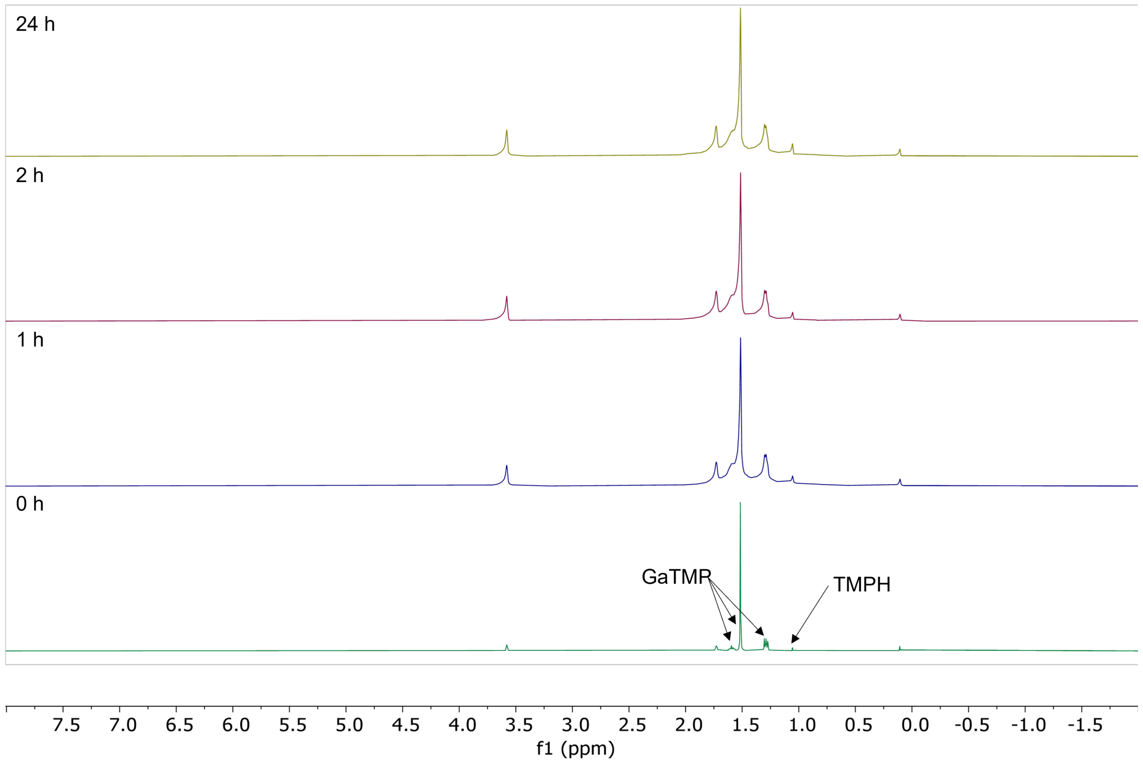

Figure S42 ^{1}H-NMR spectra of the reaction of GaTMP with 2.8 eq. Mg over the course of 24 h in THF-d_8. No changes visible.

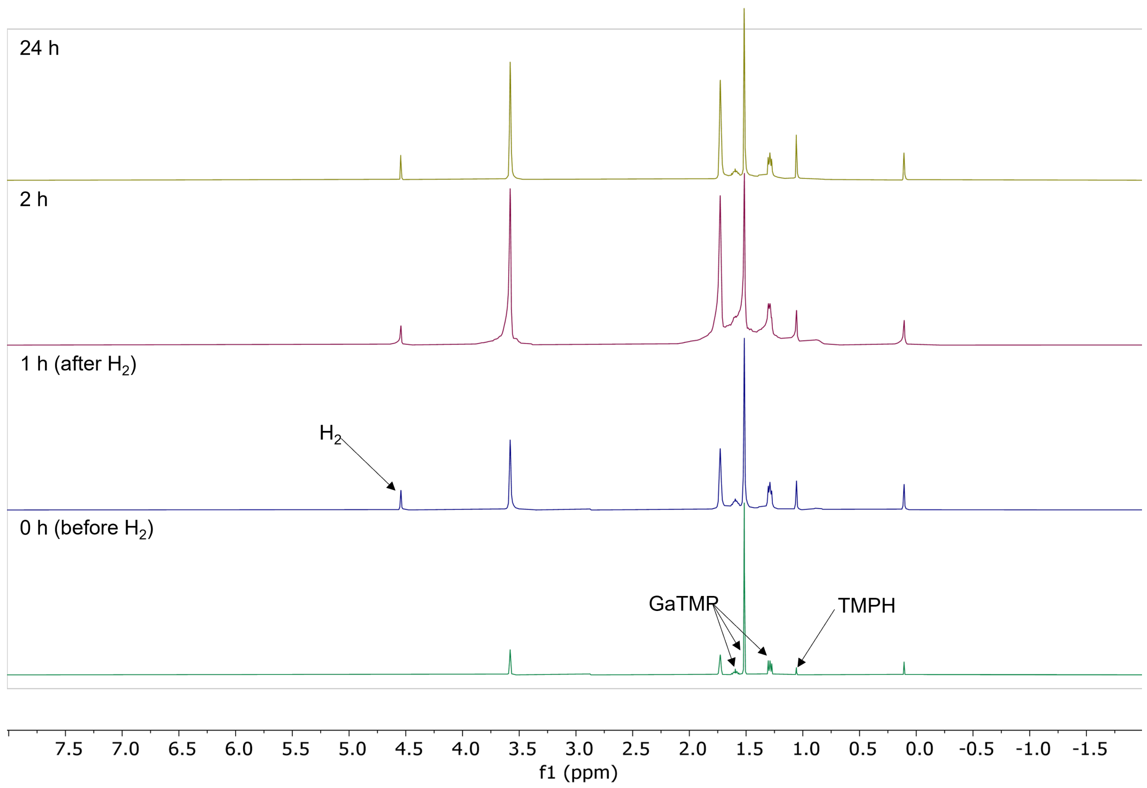

Figure S43 ^{1}H-NMR spectra of the reaction of GaTMP with 1 bar of H_2 and 2.8 eq. Mg over the course of 24 h in THF-d_8. No changes visible despite a small increase of TMPH over time, similar to experiment with only H_2 as additive (Figure S41).

IR Spectra

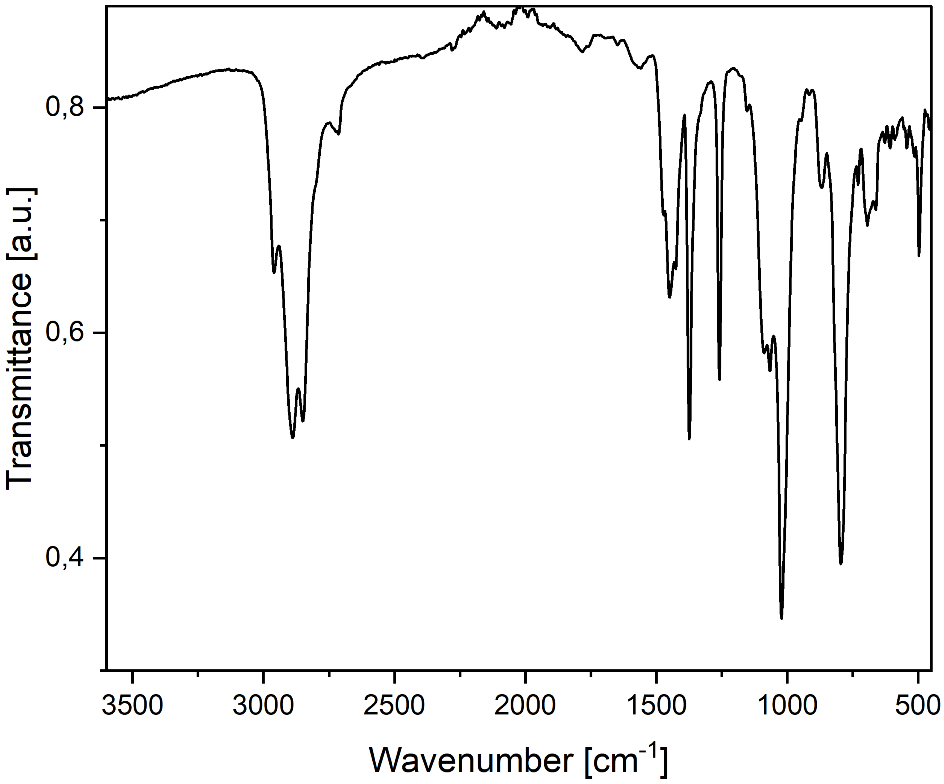

Figure S44 ATR-Infrared spectrum of the Co/Ga cluster library $[Ga_xCo_y](CoCp^*)_z$ (x = 5-11, y = 0,1 and z = 6,7).

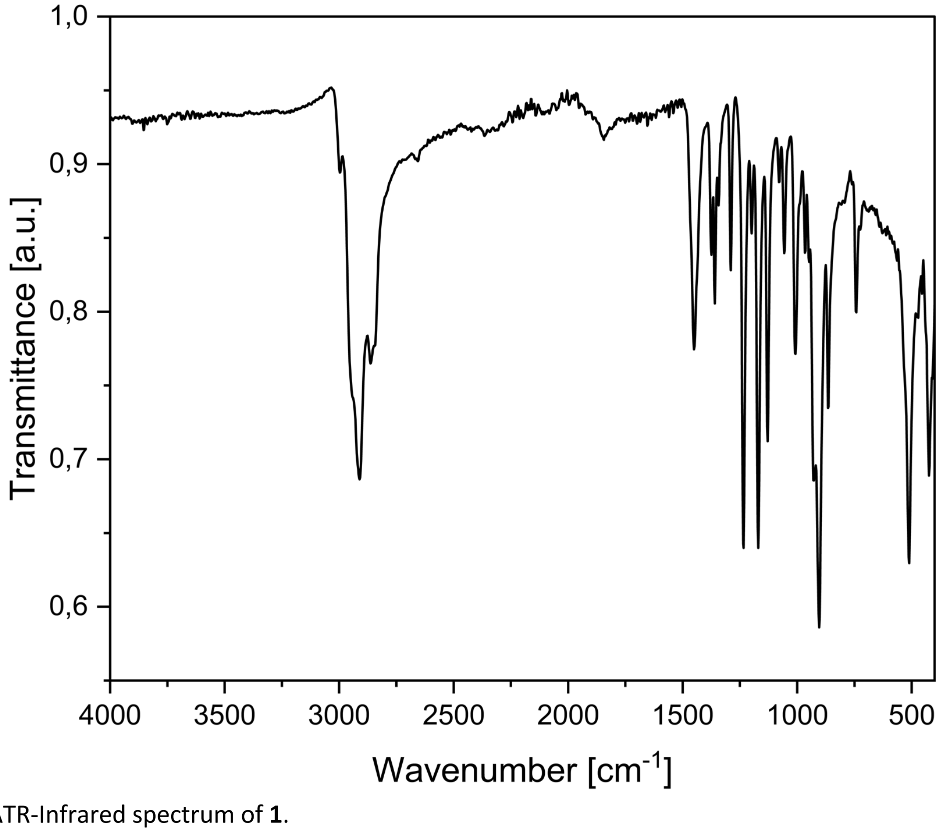

Figure S45 ATR-Infrared spectrum of **1**.

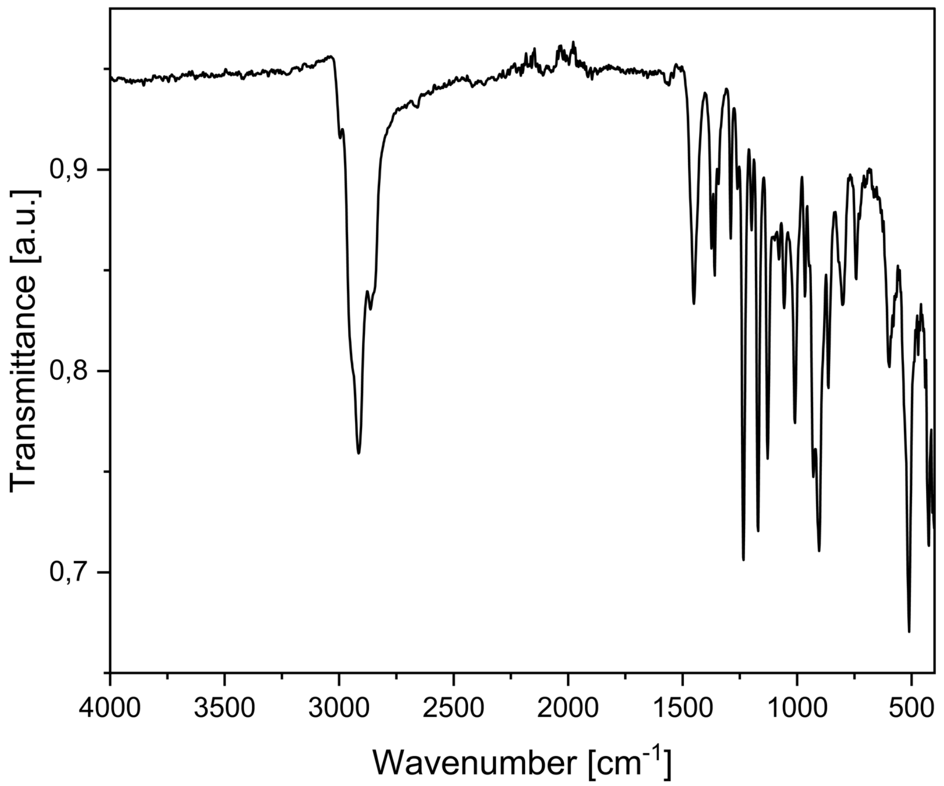

Figure S46 ATR-Infrared spectrum of **1-D**.

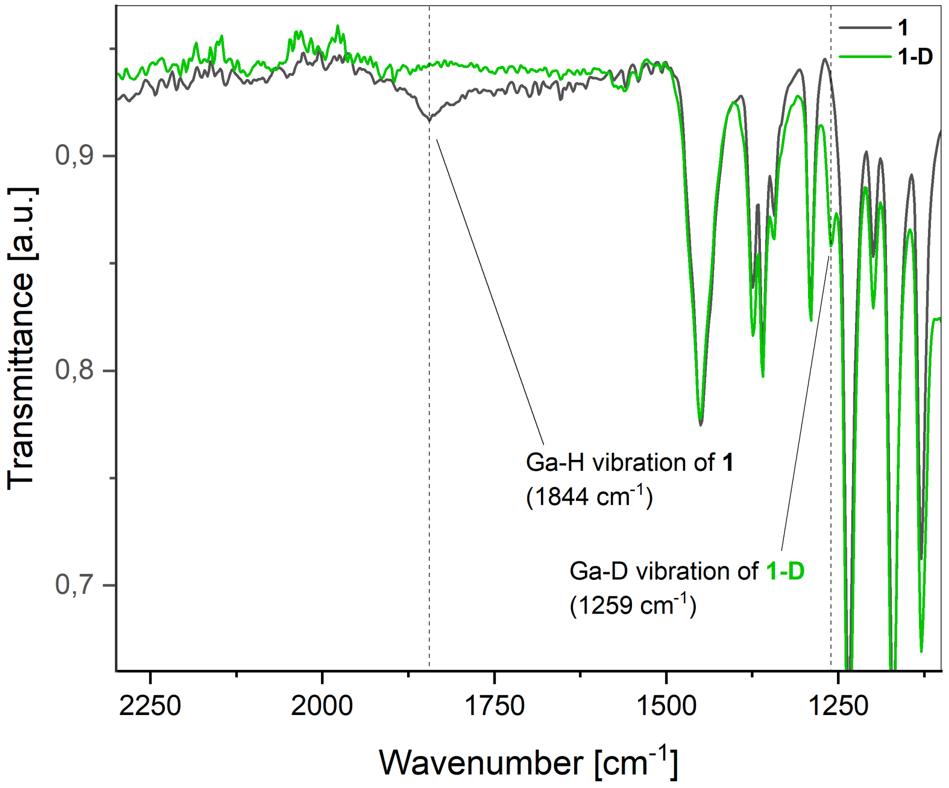

Figure S47 Cutouts of the ATR-Infrared spectra of **1** (black) and **1-D** (green).

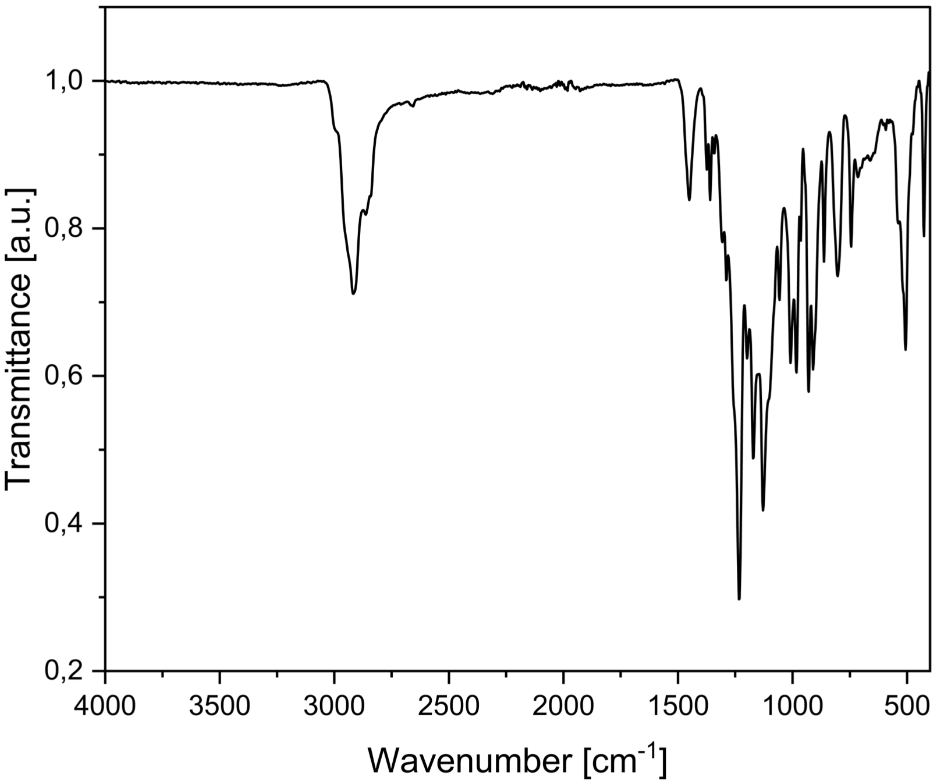

Figure S48 ATR-Infrared spectrum of **3**.

UV/Vis spectra

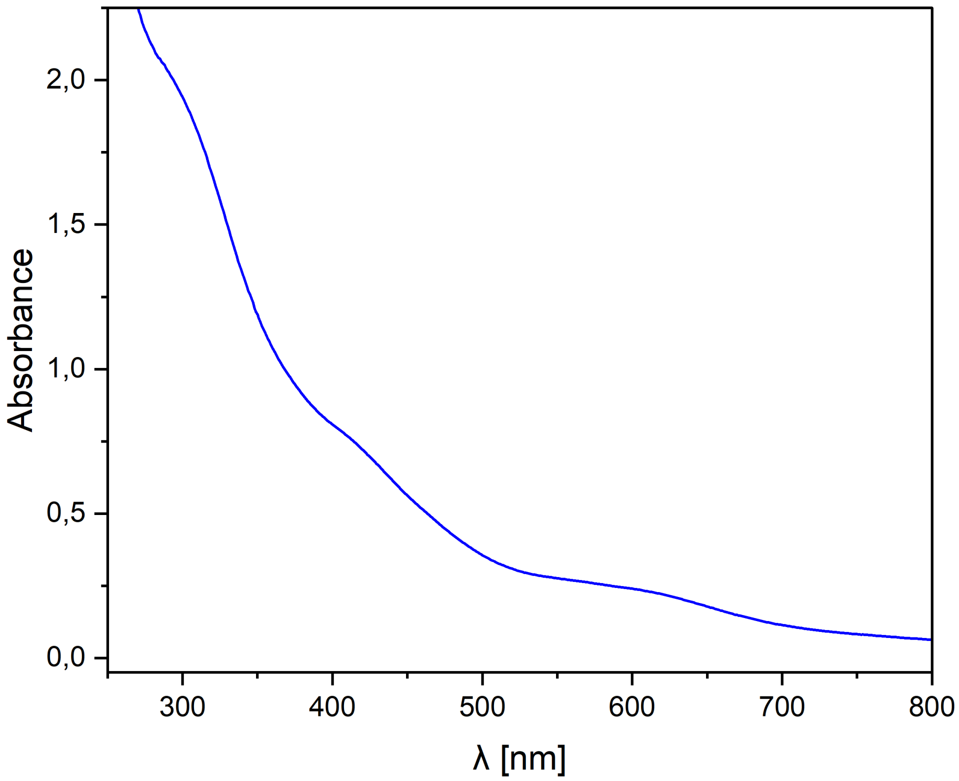

Figure S49 UV/Vis spectrum of **1**. No absorption maxima, only weak shoulders were observed: 296 (sh) nm, 410 (sh) nm, 604 (sh) nm.

LIFDI-MS spectra

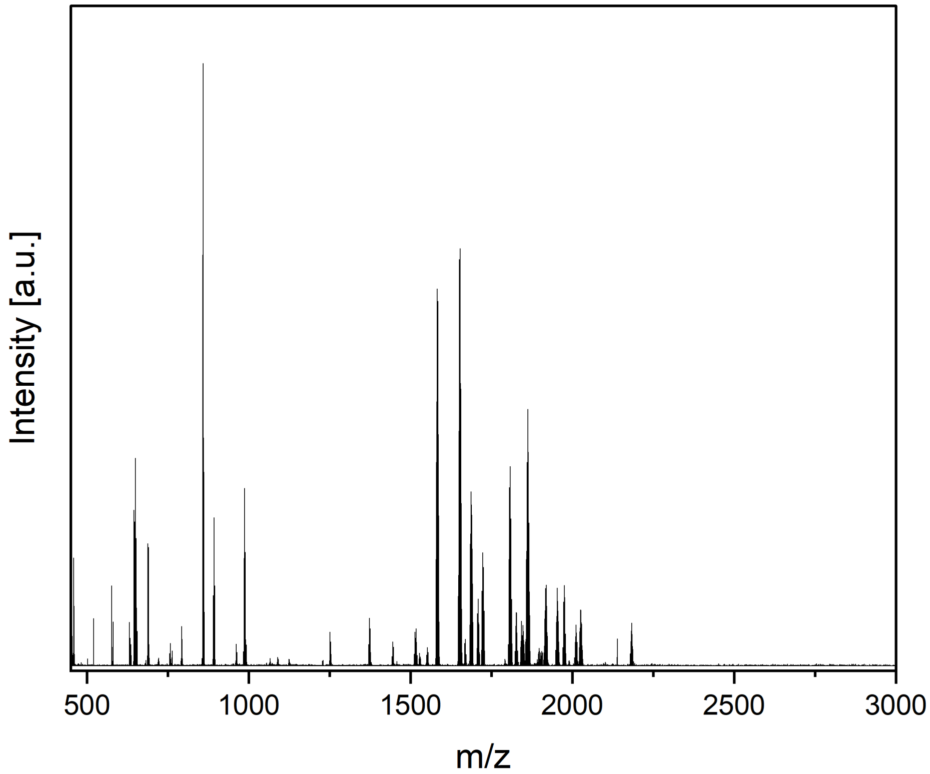

Figure S50 LIFDI mass spectrum of the Co/Ga cluster library $[Ga_xCo_y](CoCp^*)_z$ (x = 5-11, y = 0,1 and z = 6,7).

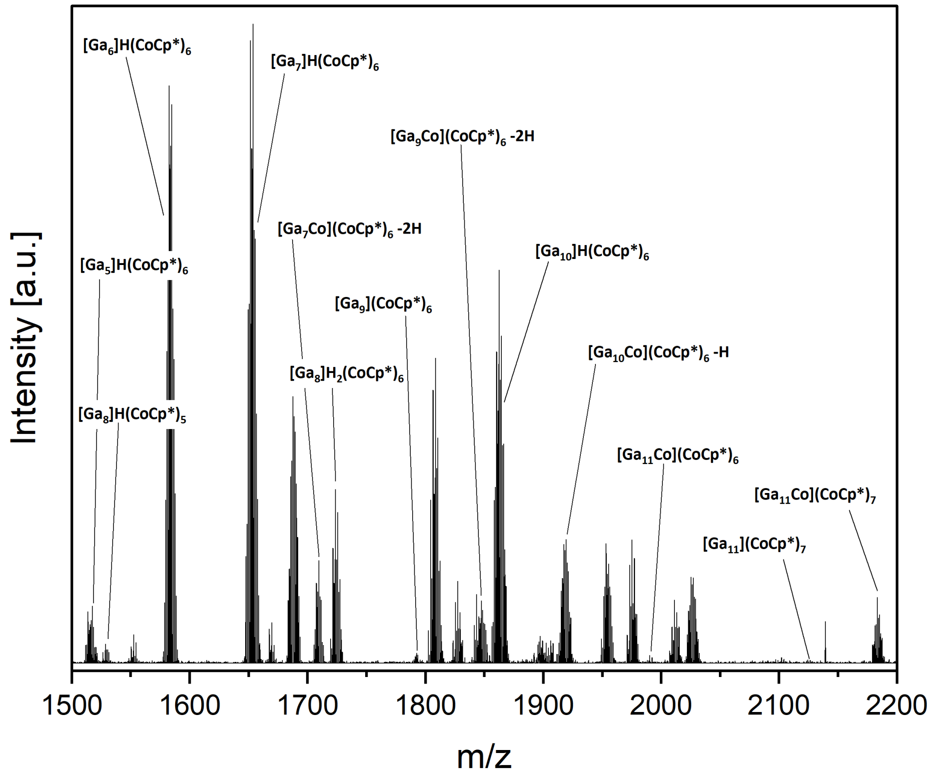

Figure S51 Cutout of the LIFDI mass spectrum of the Co/Ga cluster library $[Ga_xCo_y](CoCp^*)_z$ (x = 5-11, y = 0,1 and z = 6,7) and molecular ions of contained clusters depicted in the spectrum.

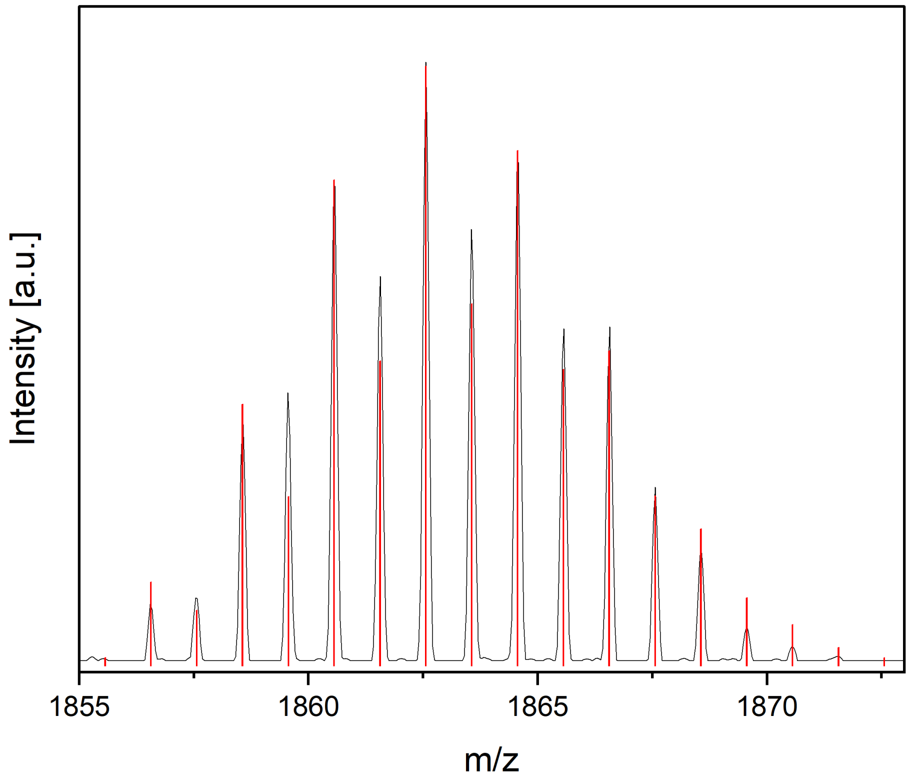

Figure S52 Cutout of the LIFDI mass spectrum of the Co/Ga cluster library $[Ga_xCo_y](CoCp^*)_z$ (x = 5-11, y = 0,1 and z = 6,7) showing the isotopic pattern of **2** (*m/z* = 1862.5625, black) and the theoretically predicted isotopic pattern of **2** (calc.: 1862.56473, red).

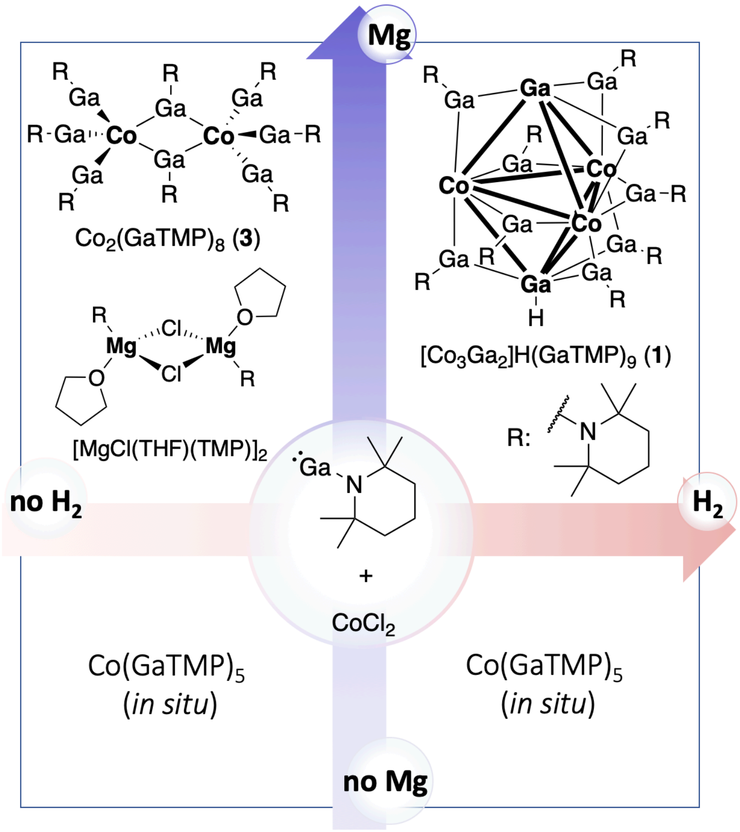

Figure S53 Overview of mixed Co/Ga heterobimetallic molecular complexes and clusters. Species variable in size, compositions and Co/Ga fraction, depending on the type and combination of reducing agents.

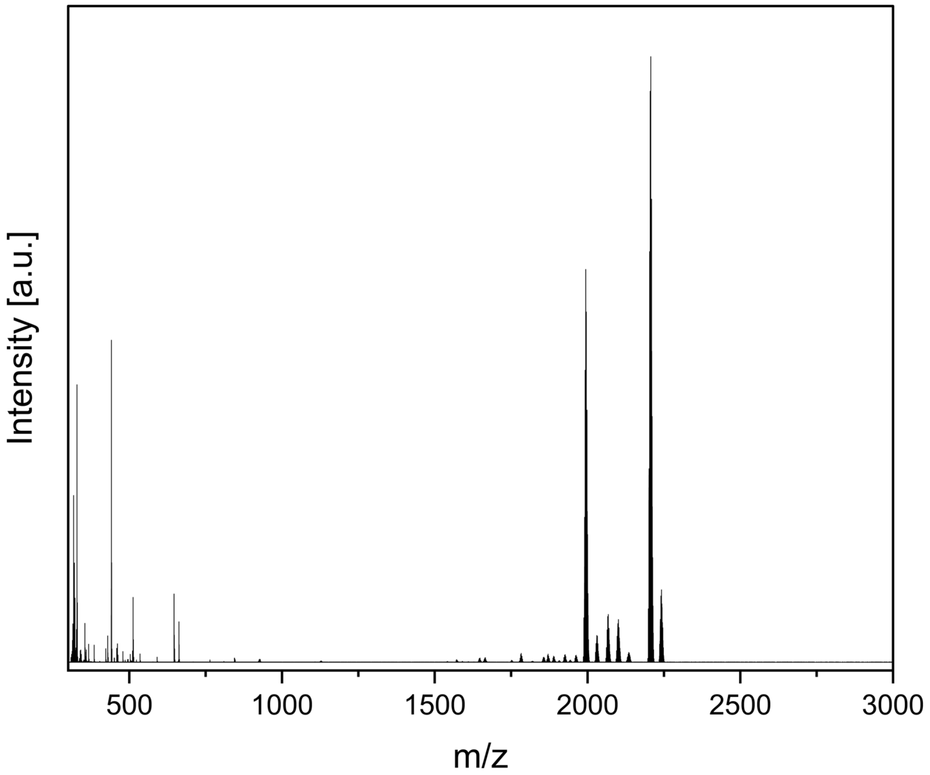

Figure S54 LIFDI mass spectrum of **1**.

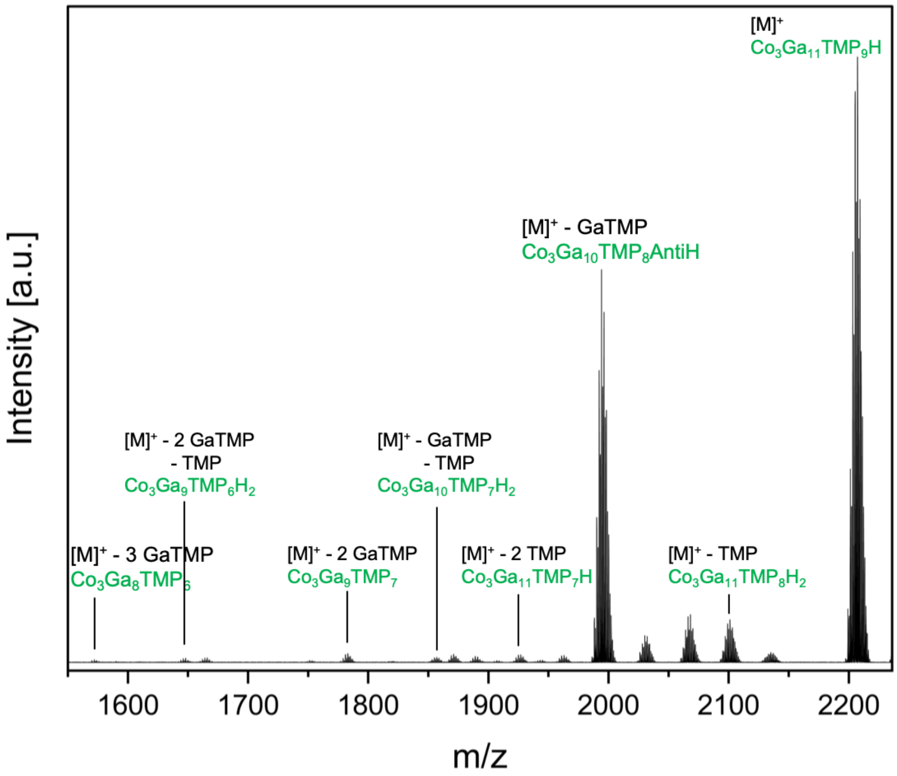

Figure S55 Cutout of the LIFDI mass spectrum of **1**. Molecular ion of **1** (*m/z* = 2207.2822) and fragment ions depicted in the spectrum.

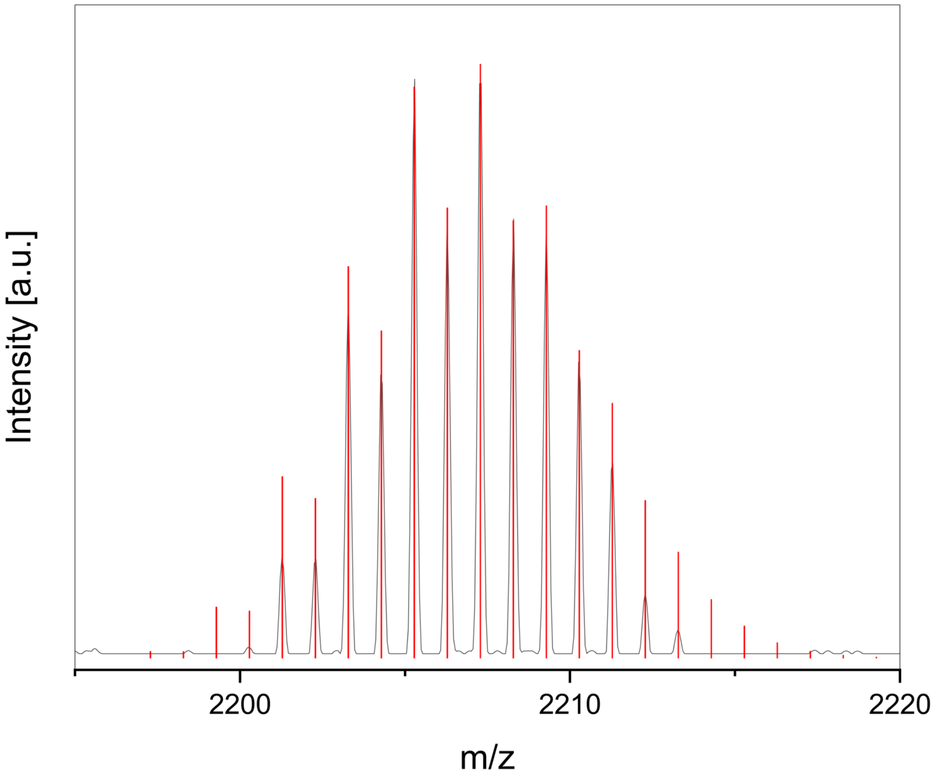

Figure S56 Cutout of the LIFDI mass spectrum of **1** showing the isotopic pattern of **1** (*m/z* = 2207.2822, black) and the theoretically predicted isotopic pattern of **1** (calc.: 2207.28215, red).

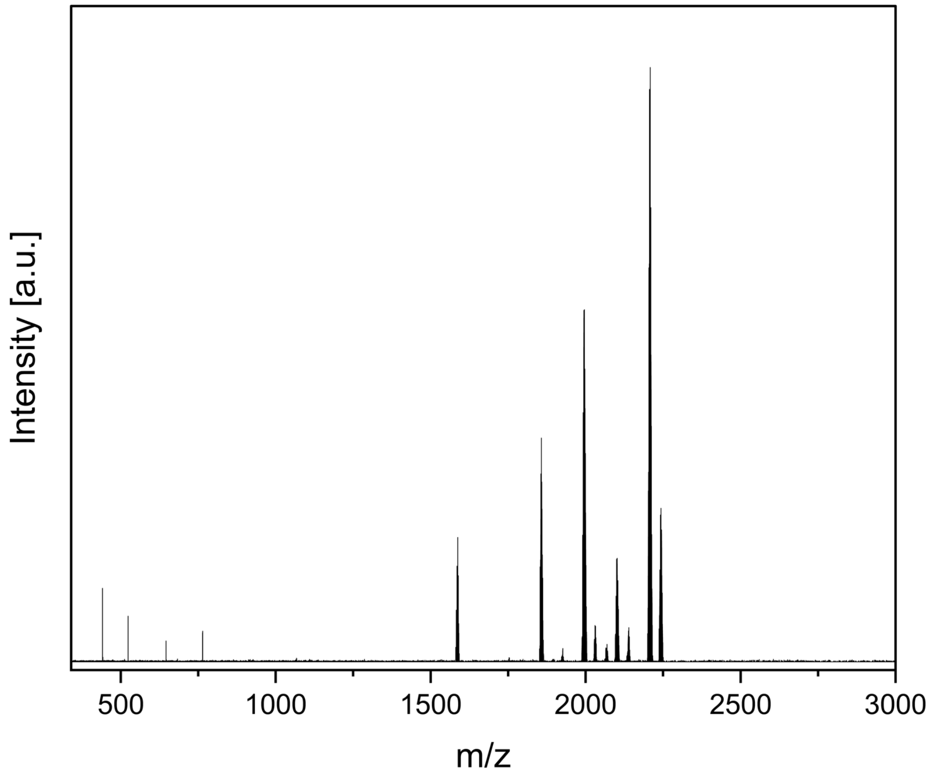

Figure S57 LIFDI mass spectrum of **1-D**.

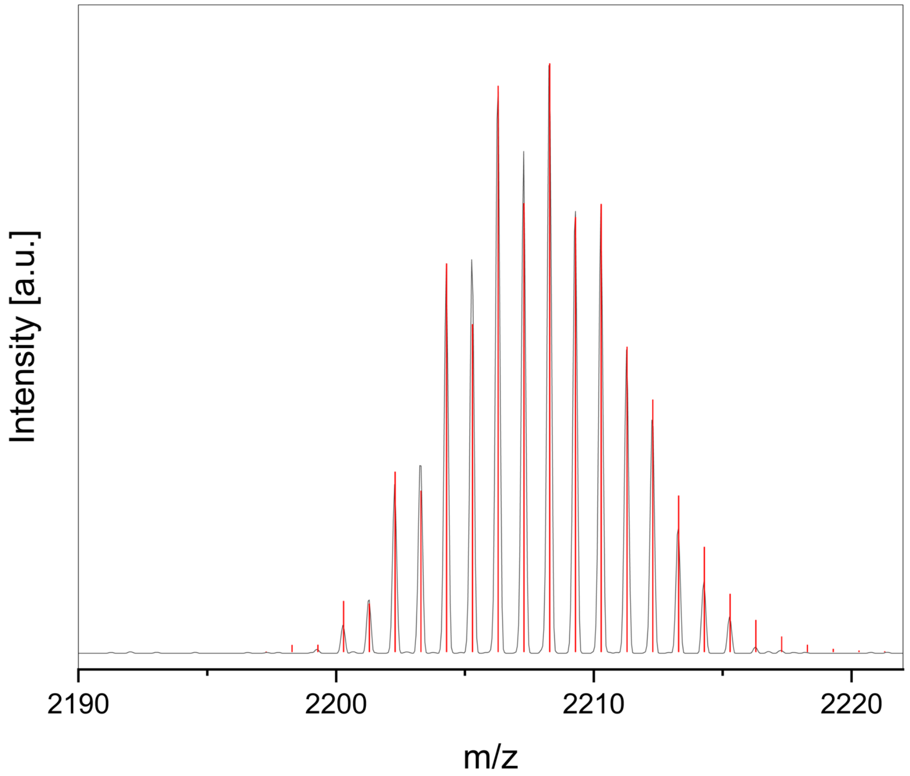

Figure S58 Cutout of the LIFDI mass spectrum of **1-D** showing the isotopic pattern of **1-D** (*m/z* = 2208.2763, black) and the theoretically predicted isotopic pattern of **1-D** (calc.: 2208.28843, red).

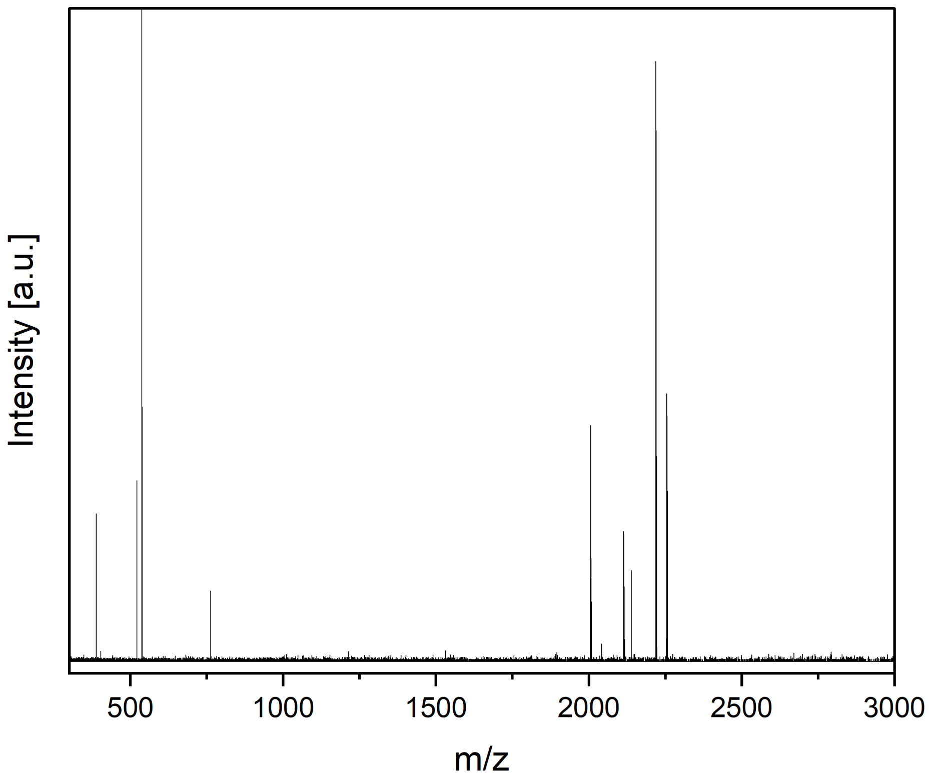

Figure S59 LIFDI mass spectrum of **1-^{71}Ga-D**.

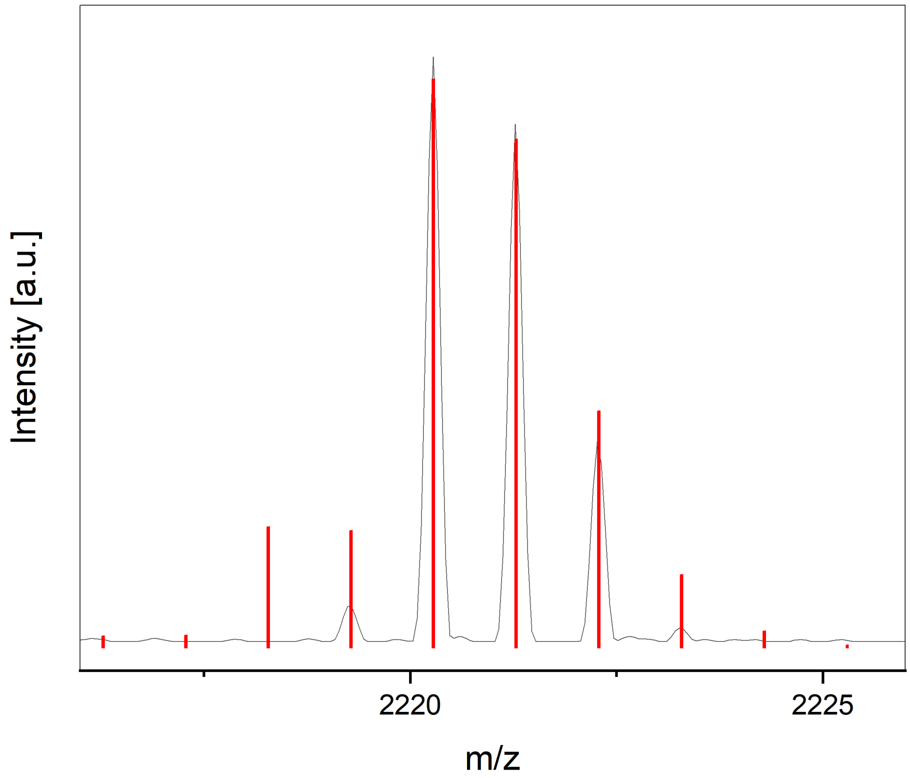

Figure S60 Cutout of the LIFDI mass spectrum of **1-^{71}Ga-D** showing the isotopic pattern of **1-^{71}Ga-D** (*m/z* = 2220.2809, black) and the theoretically predicted isotopic pattern of **1-^{71}Ga-D** (calc.: 2220.28138, red).

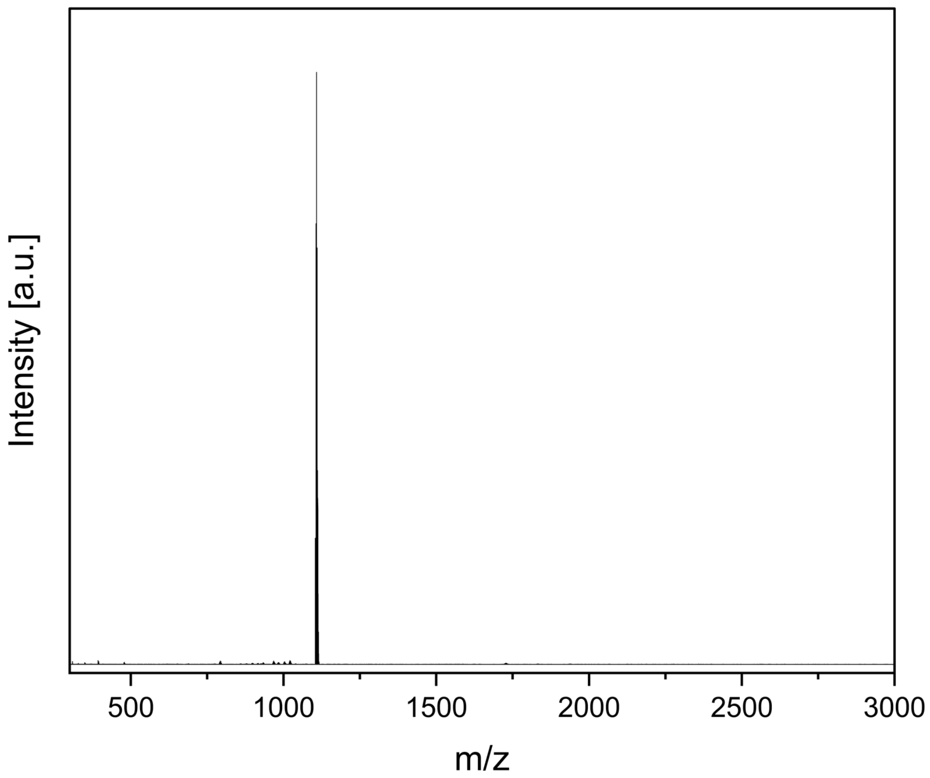

Figure S61 LIFDI mass spectrum of Co(GaTMP)$_5$, achieved by the reaction of $CoCl_2$ with GaTMP without the addition of Mg and H_2.

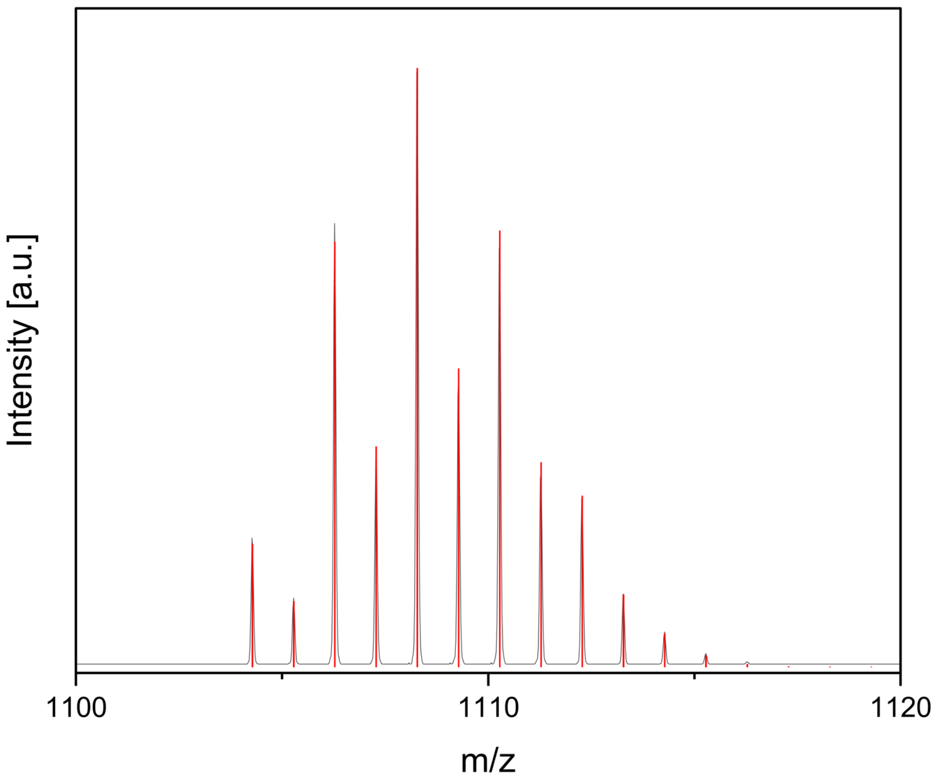

Figure S62 Cutout of the LIFDI mass spectrum of Co(GaTMP)$_5$ (synthesis without Mg/H_2) showing the isotopic pattern of [Co(GaTMP)$_5$]$^+$ (*m/z* = 1108,2824 black) and the theoretically predicted isotopic pattern of [Co(GaTMP)$_5$]$^+$ (calc.: 1108.27959, red).

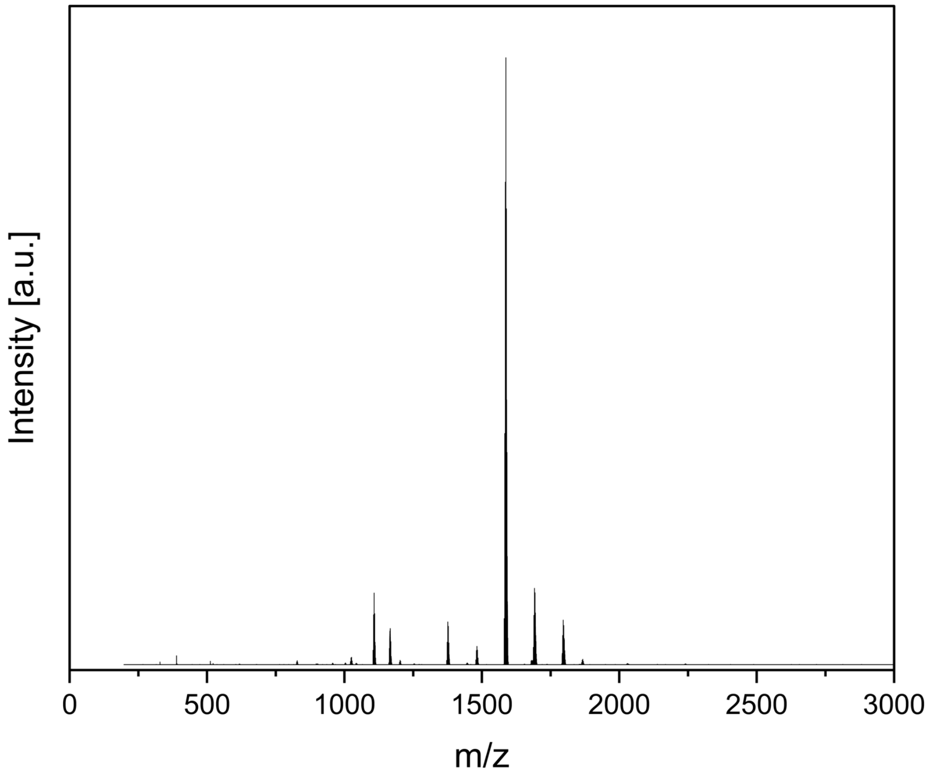

Figure S63 LIFDI mass spectrum of **3**.

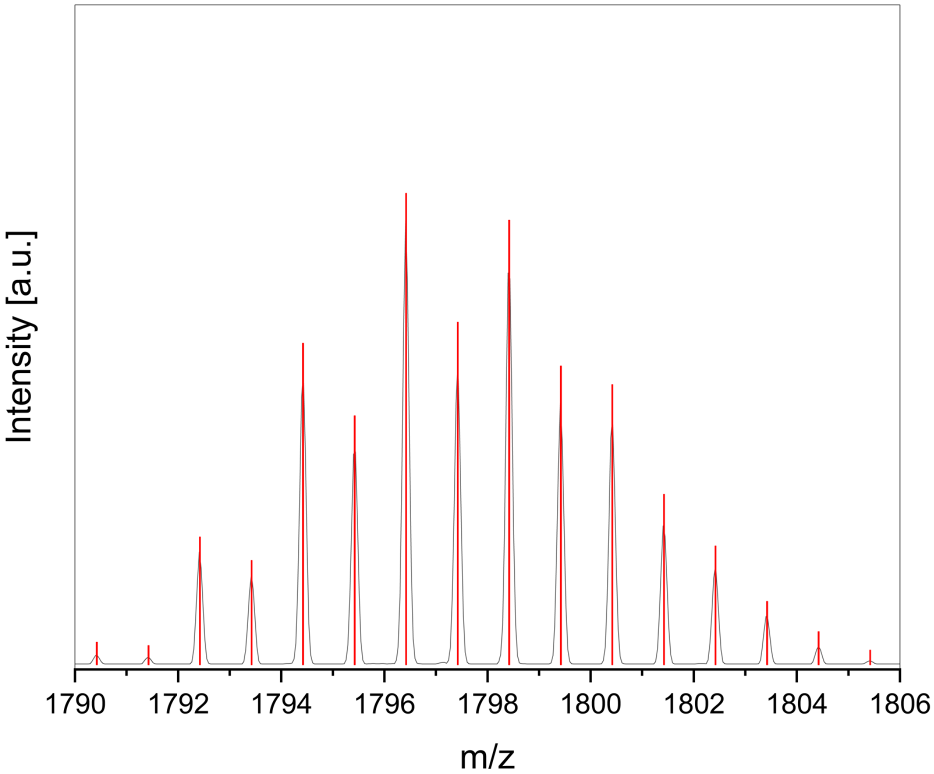

Figure S64 Cutout of the LIFDI mass spectrum of **3** showing the isotopic pattern of **3** (*m/z* = 1796.4160, black) and the theoretically predicted isotopic pattern of **3** (calc.: 1796.42128, red).

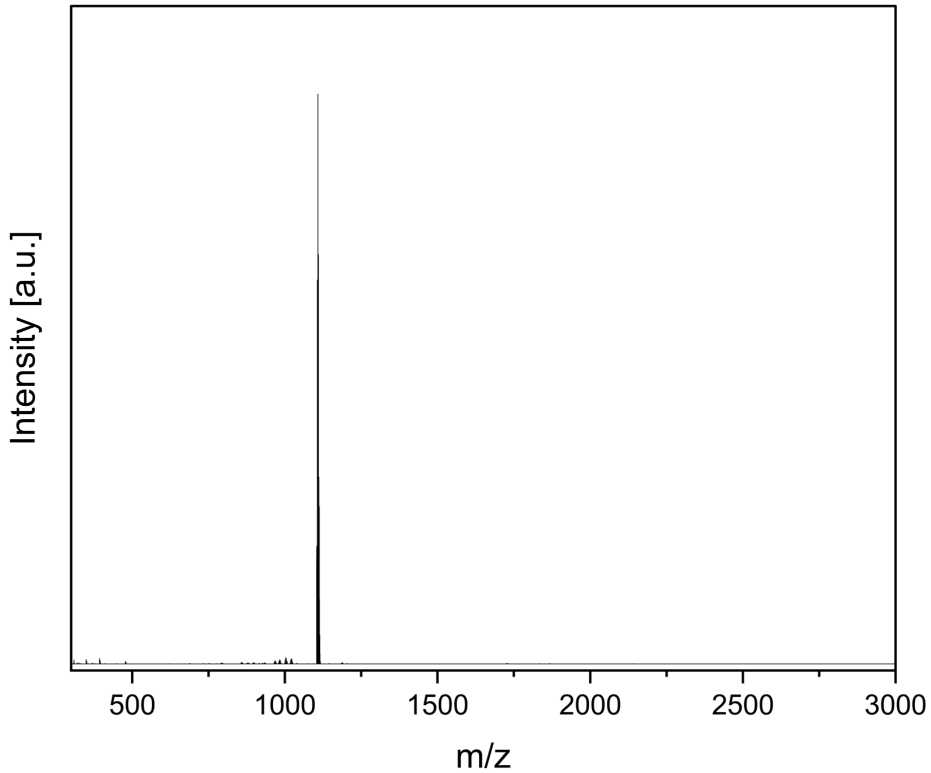

Figure S65 LIFDI mass spectrum of $Co(GaTMP)_5$, achieved by the reaction of $CoCl_2$ with GaTMP with only the addition of H_2.

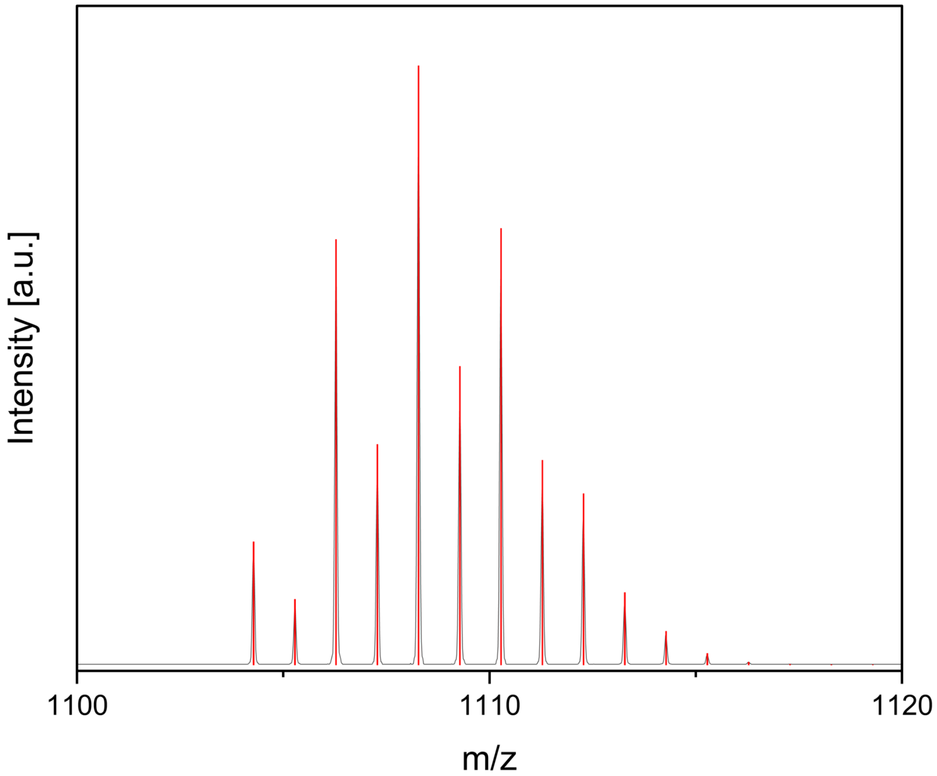

Figure S66 Cutout of the LIFDI mass spectrum of $Co(GaTMP)_5$ (synthesis only with H_2) showing the isotopic pattern of $[Co(GaTMP)_5]^+$ (*m/z* = 1108,2790, black) and the theoretically predicted isotopic pattern of $[Co(GaTMP)_5]^+$ (calc.: 1108.27959, red).

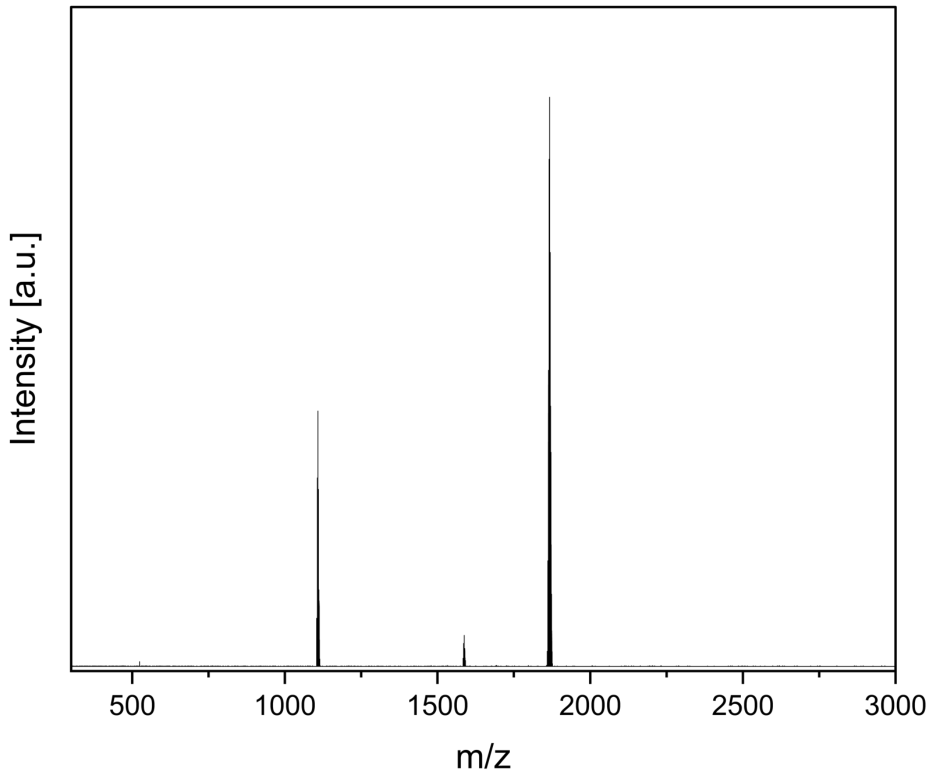

Figure S67 LIFDI mass spectrum of the reaction intermediate $[Co_2Ga](GaTMP)_8$ during the synthesis of **1** after a reaction time of 4 h (pattern at 1867.3422 m/z; pattern at m/z = 1108.6728 and m/z = 1587.4214 are $[Co(GaTMP)_5]^+$ and $[Co_2(GaTMP)_7]^+$, respectively).

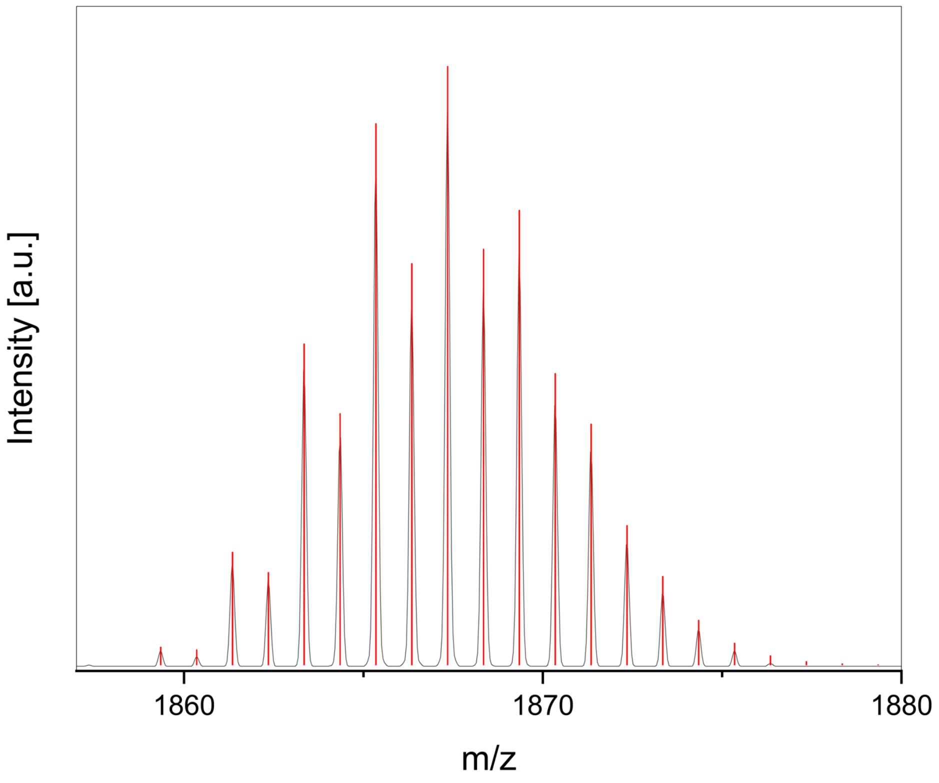

Figure S68 Cutout of the LIFDI mass spectrum of $[Co_2Ga](GaTMP)_8$ showing the isotopic pattern of $[[Co_2Ga](GaTMP)_8]^+$ (*m/z* = 1867.3422, black) and the theoretically predicted isotopic pattern of $[[Co_2Ga](GaTMP)_8]^+$ (calc.: 1867.34640, red).

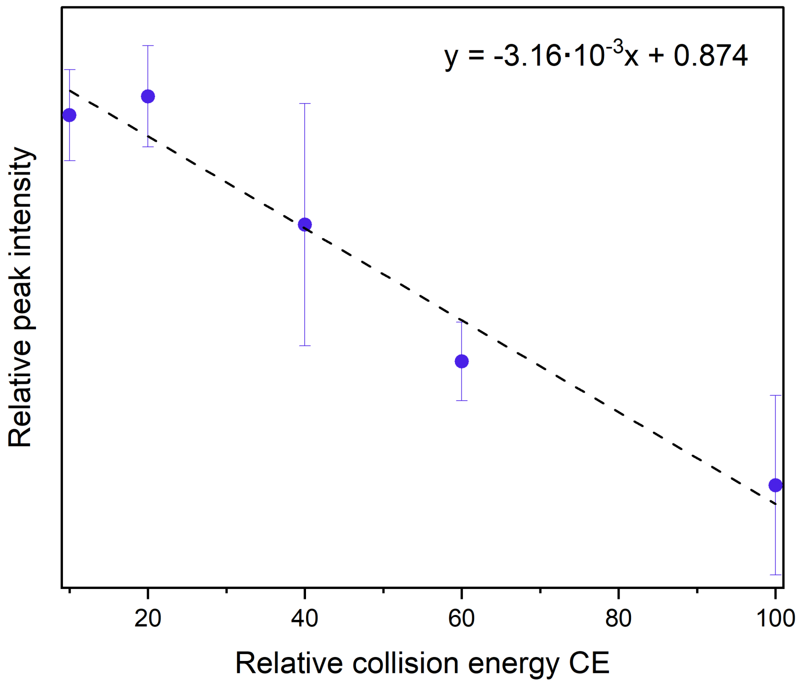

Figure S69 Relative peak intensity of the ion $[Co(GaTMP)_5]^+$ observed *via* LIFDI-mass spectrometry as a function of the relative collision energy CE. Due to the negative slope of $-3.16*10^{-3}$ of the regression, the species $[Co(GaTMP)_5]^+$ is assigned as molecular ion. Beside the collision energy, the set of measurements was performed under identical measurement conditions.

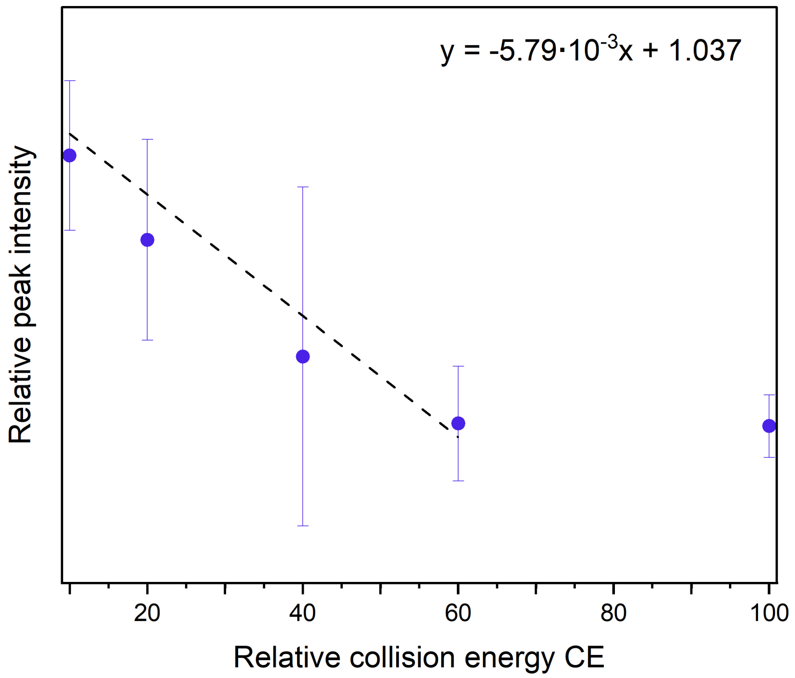

Figure S70 Relative peak intensity of the ion $[[Co_2Ga](GaTMP)_8]^+$ observed *via* LIFDI-mass spectrometry as a function of the relative collision energy CE. Due to the negative slope of $-5.79*10^{-3}$ of the regression, the species $[(Co_2Ga)(GaTMP)_8]^+$ is assigned as molecular ion. Only peak intensities up to a collision energy of 60 CE were considered, since for CE 100, no change in peak intensity could be observed. Beside the collision energy, the set of measurements was performed under identical measurement conditions.

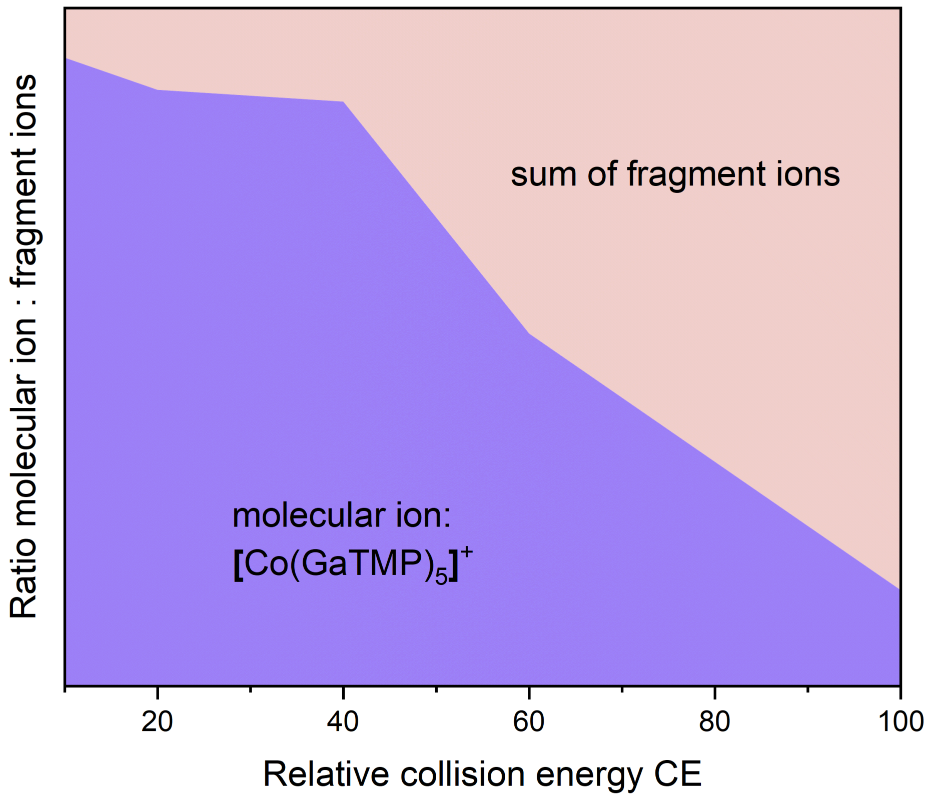

Figure S71 Ratio of the normalized peak intensity of the identified molecular ion $[Co(GaTMP)_5]^+$ and the sum of fragment ions as function of the relative collision energy.

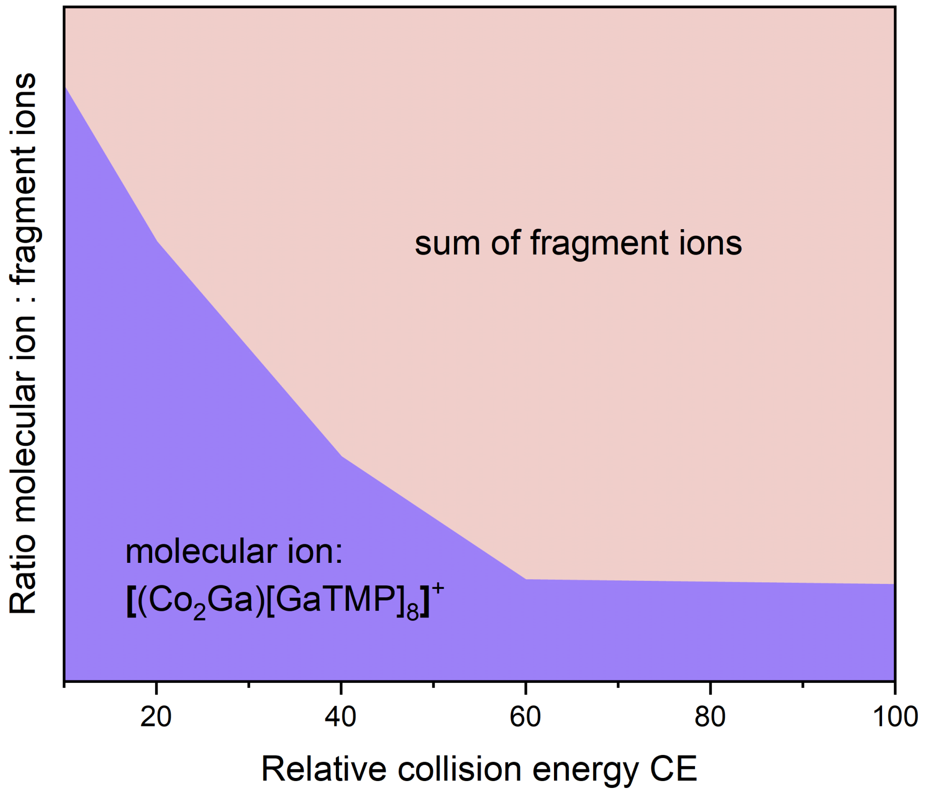

Figure S72 Ratio of the normalized peak intensity of the identified molecular ion $[[Co_2Ga](GaTMP)_8]^+$ and the sum of fragment ions as function of the relative collision energy.

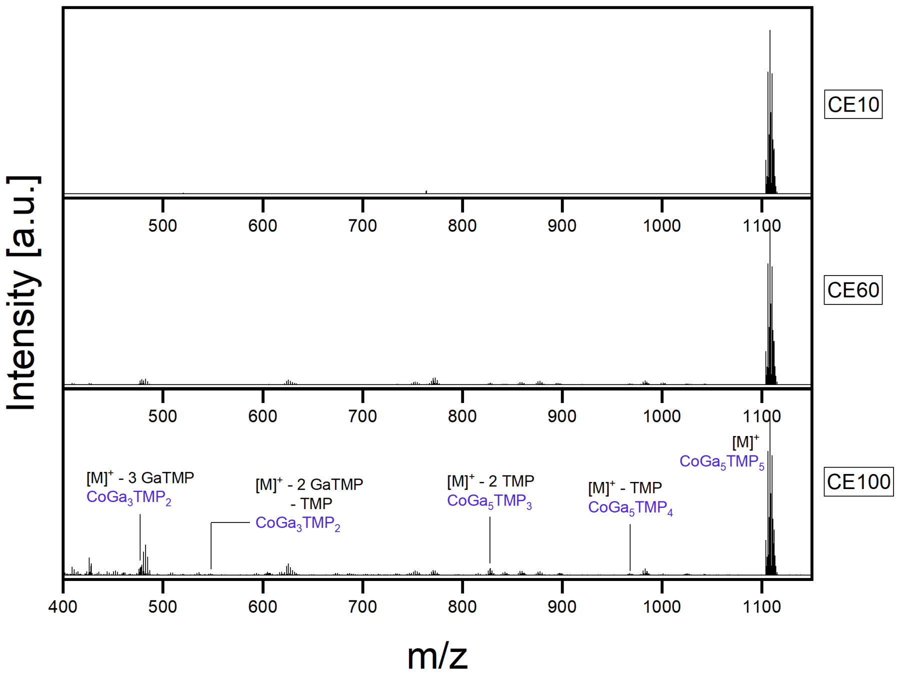

Figure S73 LIFDI mass spectra of $[Co(GaTMP)_5]^+$ over increasing relative collision energy and allocation of selected fragment ions to the pattern in the spectrum with highest collision energy (bottom).

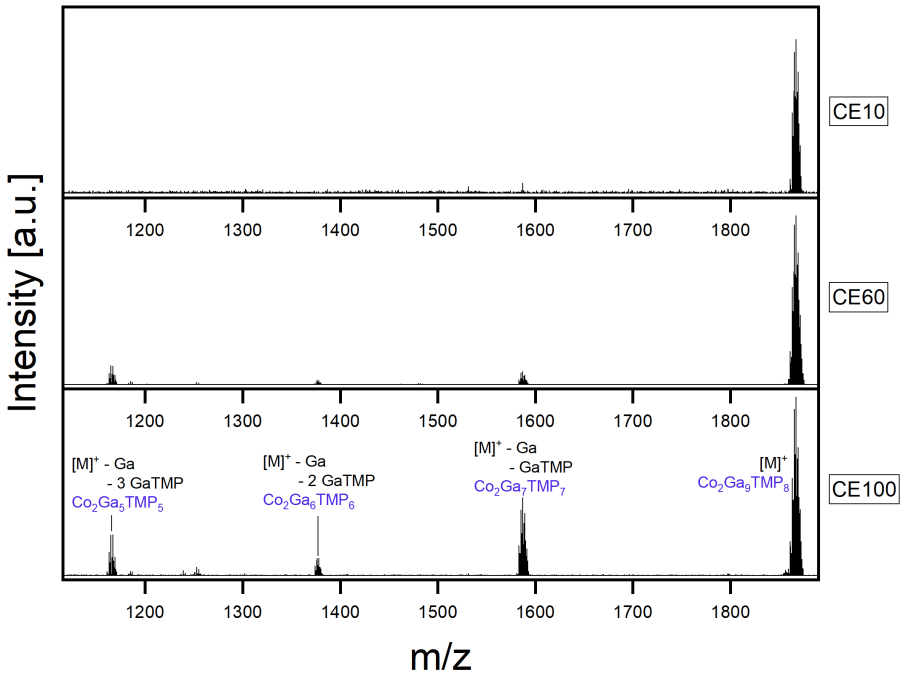

Figure S74 LIFDI mass spectra of $[[Co_2Ga](GaTMP)_8]^+$ over increasing relative collision energy and allocation of selected fragment ions to the pattern in the spectrum with highest collision energy (bottom).

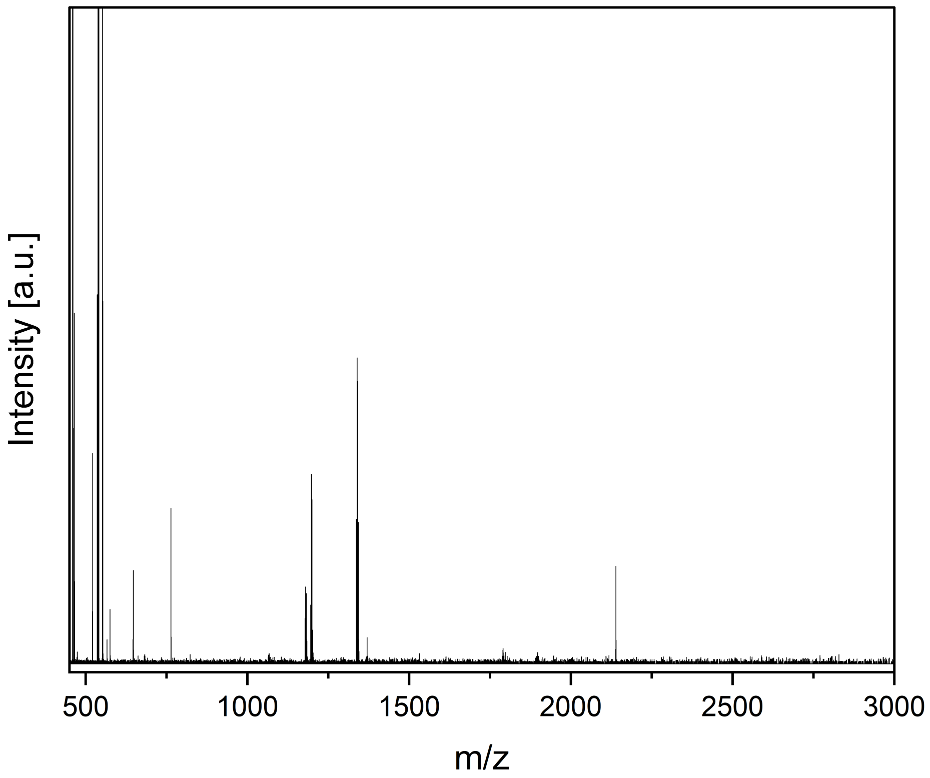

Figure S75 LIFDI mass spectrum of **4**.

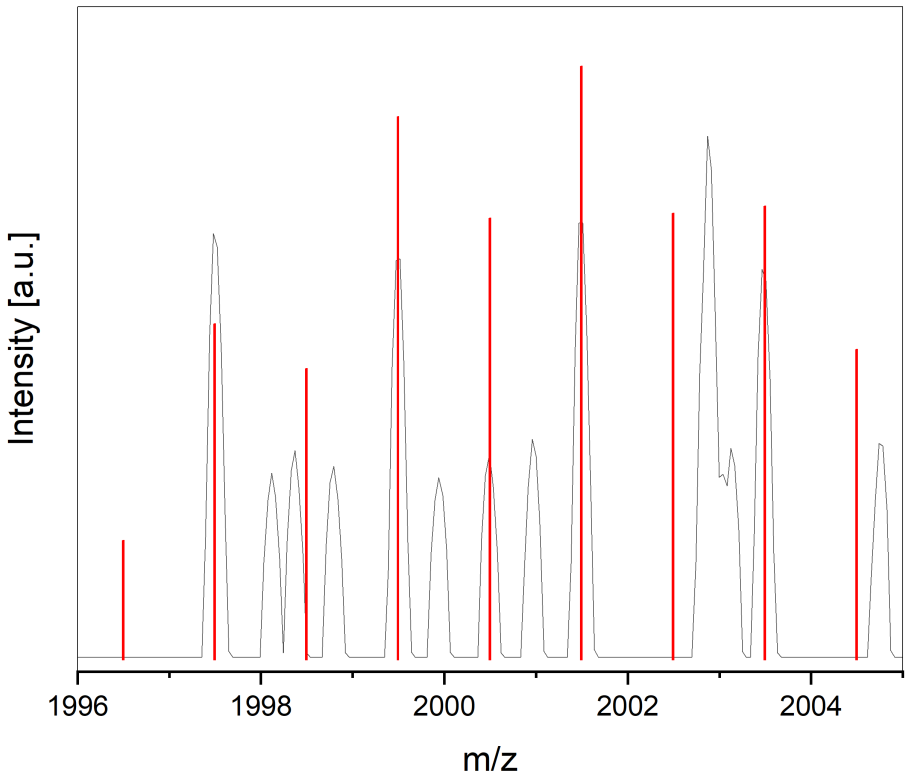

Figure S76 Cutout of the LIFDI mass spectrum of **4** showing the isotopic pattern of **4** (*m/z* = 2001.5098, black) in a low intensity and the theoretically predicted isotopic pattern of **4** (calc.: 2001.49470, red, see also extended discussion on **4**).

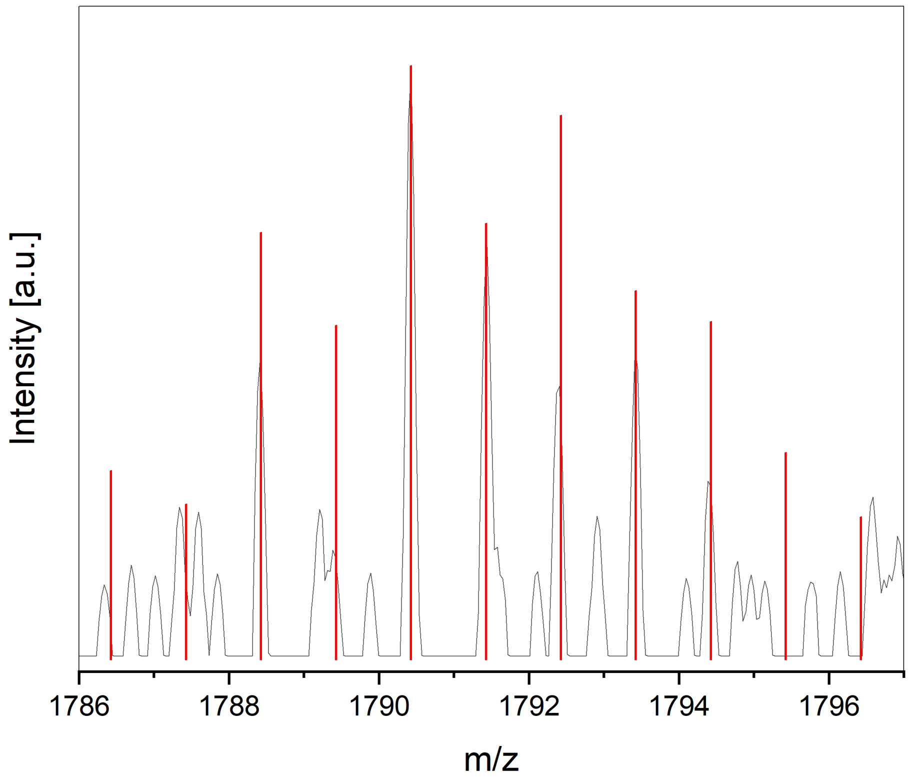

Figure S77 Cutout of the LIFDI mass spectrum of **4** showing the isotopic pattern of [M-GaTMP]$^+$ (*m/z* = 1790.4278, black) in a low intensity and the theoretically predicted isotopic pattern of **4** (calc.: 1790.42533, red, see also extended discussion on **4**).

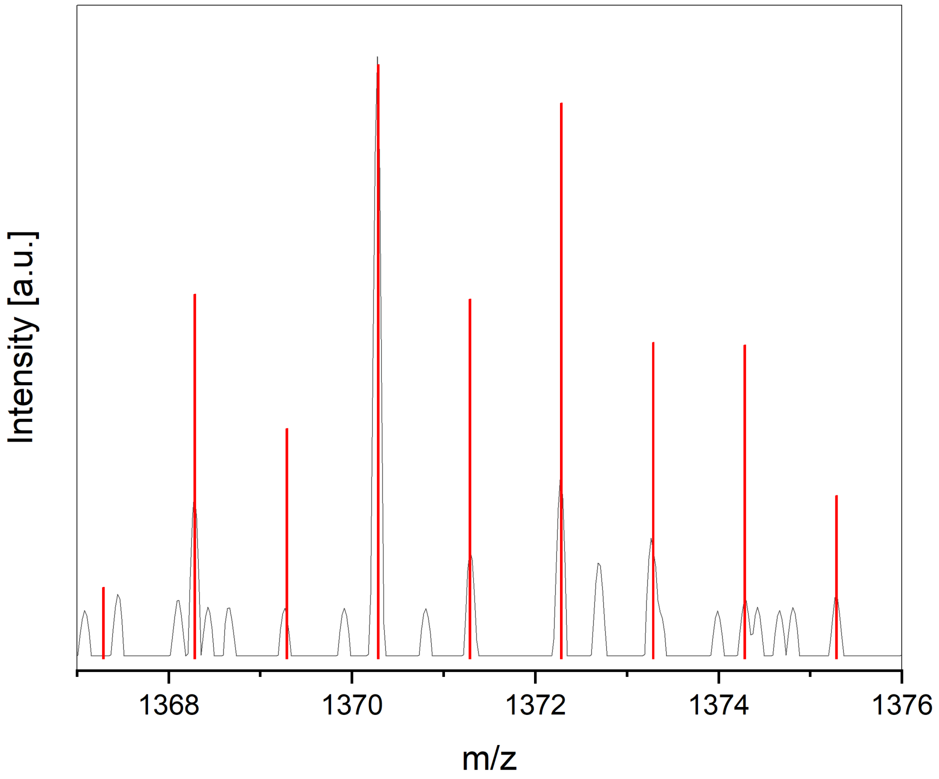

Figure S78 Cutout of the LIFDI mass spectrum of **4** showing the isotopic pattern of [M-3GaTMP]$^+$ (*m/z* = 1370.2768, black) in a low intensity and the theoretically predicted isotopic pattern of **4** (calc.: 1370.28642, red, see also extended discussion on **4**).

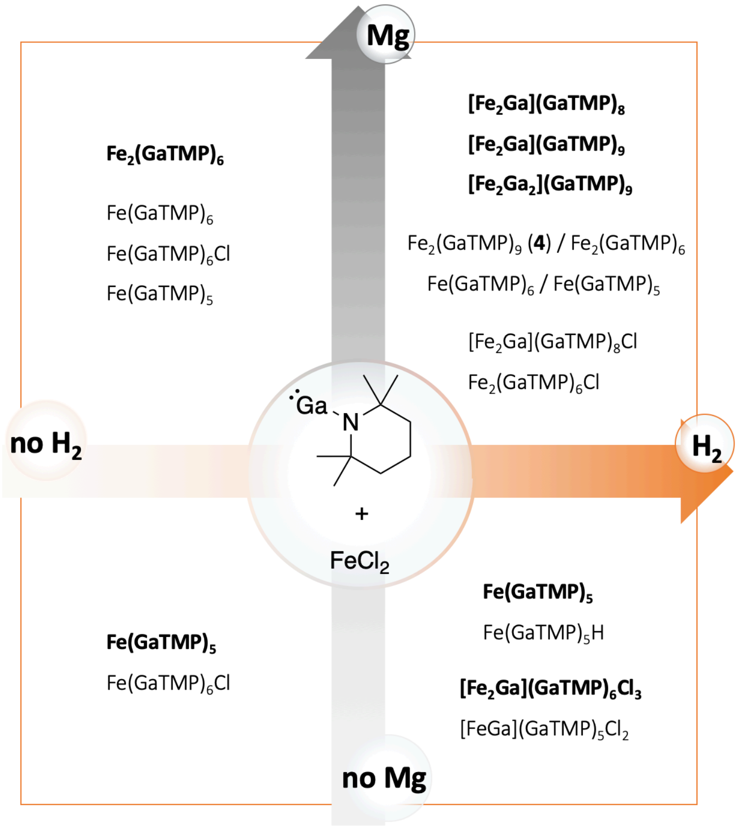

Figure S79 Overview of mixed Fe/Ga heterobimetallic molecular complexes and clusters observed *via* LIFDI-MS, following the analogous synthetic concept as for Co/Ga species. Species variable in size, compositions and Fe/Ga fraction, depending on the type and combination of reducing agents.

In the following, the individual mass spectra and the associated theoretical pattern are depicted, starting with $FeCl_2$ as precursor, followed by the spectra of the aryl-/amide-precursors ($FeMes_2$/$FeTMP_2$), every time in the following sequence of the mass spectra: H_2/Mg as additive, only H_2, only Mg, no additive.

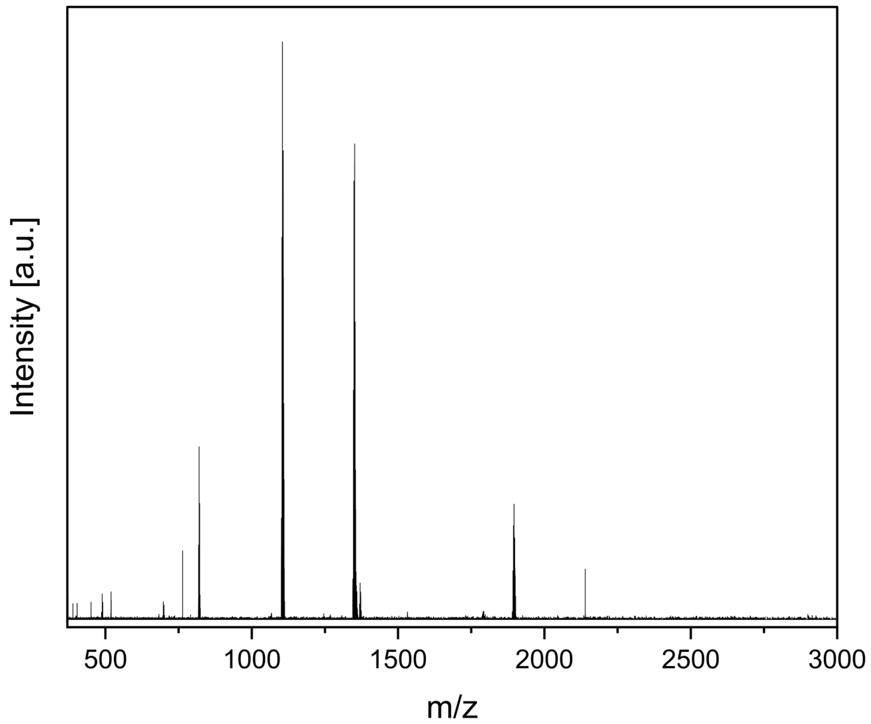

Figure S80 LIFDI mass spectrum of the reaction of $FeCl_2$ with GaTMP in THF in presence of Mg powder and H_2 after a reaction time of 18 h.

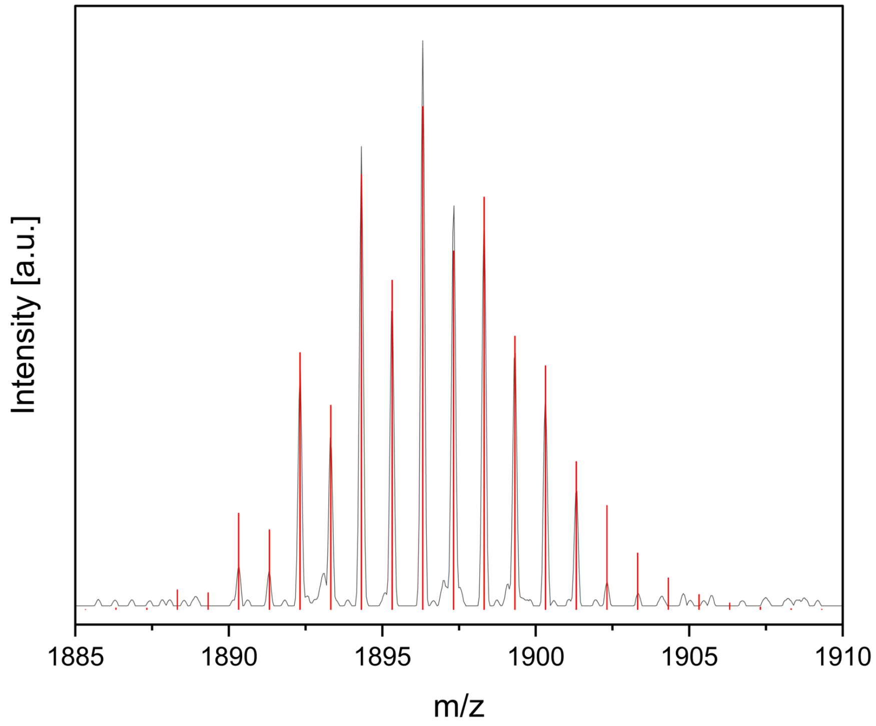

Figure S81 Cutout of the LIFDI mass spectrum of the reaction of $FeCl_2$ with GaTMP in THF in presence of Mg powder and H_2 after a reaction time of 18 h showing the isotopic pattern of $[[Fe_2Ga](GaTMP)_8Cl]^+$ (*m/z* = 1896.4919, black) and the theoretically predicted isotopic pattern of $[[Fe_2Ga](GaTMP)_8Cl]^+$ (calc.: 1896.31874, red).

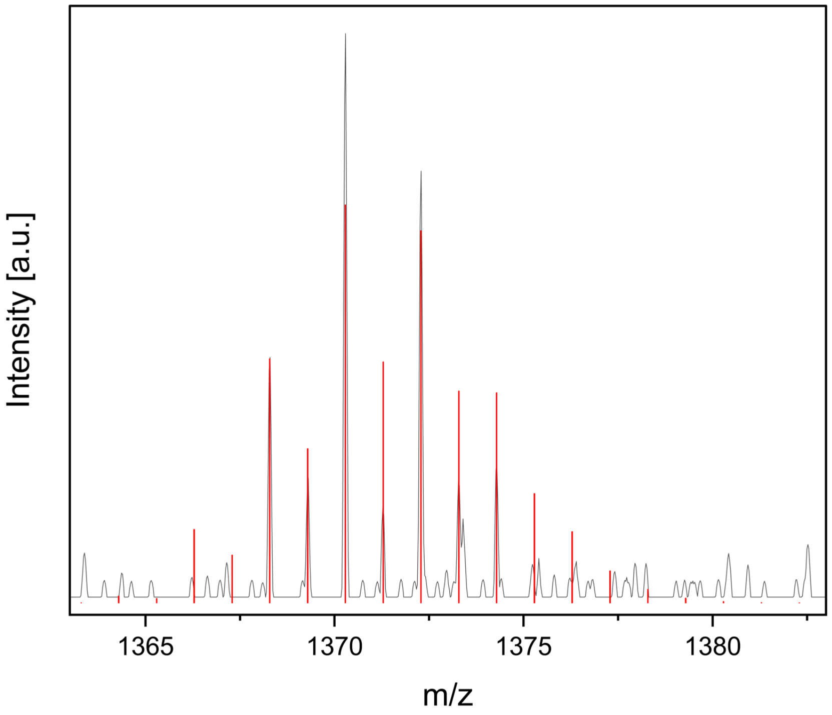

Figure S82 Cutout of the LIFDI mass spectrum of the reaction of $FeCl_2$ with GaTMP in THF in presence of Mg powder and H_2 after a reaction time of 18 h showing the isotopic pattern of $[Fe_2(GaTMP)_6]^+$ (*m/z* = 1370.2908, black) and the theoretically predicted isotopic pattern of $[Fe_2(GaTMP)_6]^+$ (calc.: 1370.28647, red).

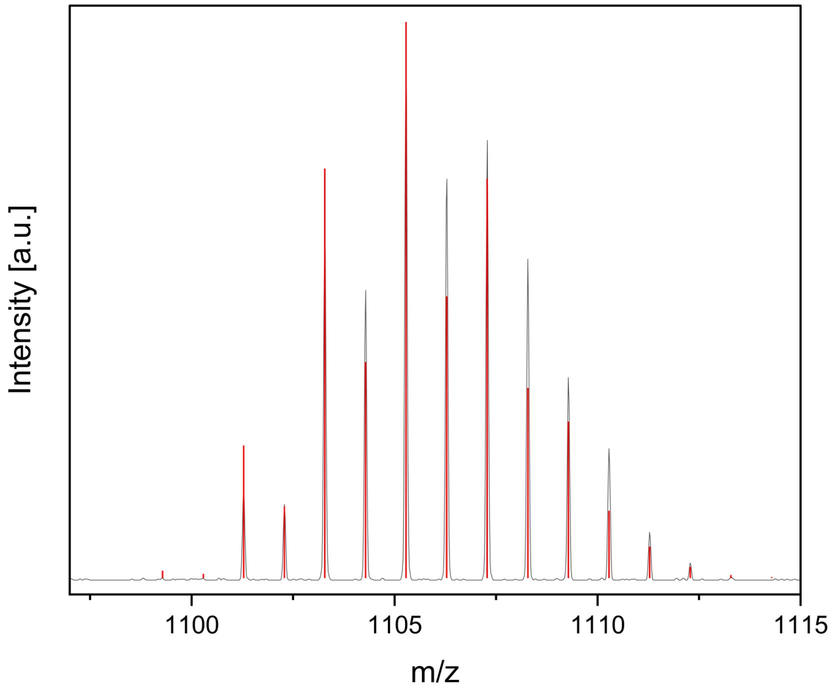

Figure S83 Cutout of the LIFDI mass spectrum of the reaction of $FeCl_2$ with GaTMP in THF in presence of Mg powder and H_2 after a reaction time of 18 h showing the isotopic pattern of $[Fe(GaTMP)_5]^+$ (*m/z* = 1105.2788, black) and the theoretically predicted isotopic pattern of $Fe(GaTMP)_5$ (calc.: 1105.28161, red).

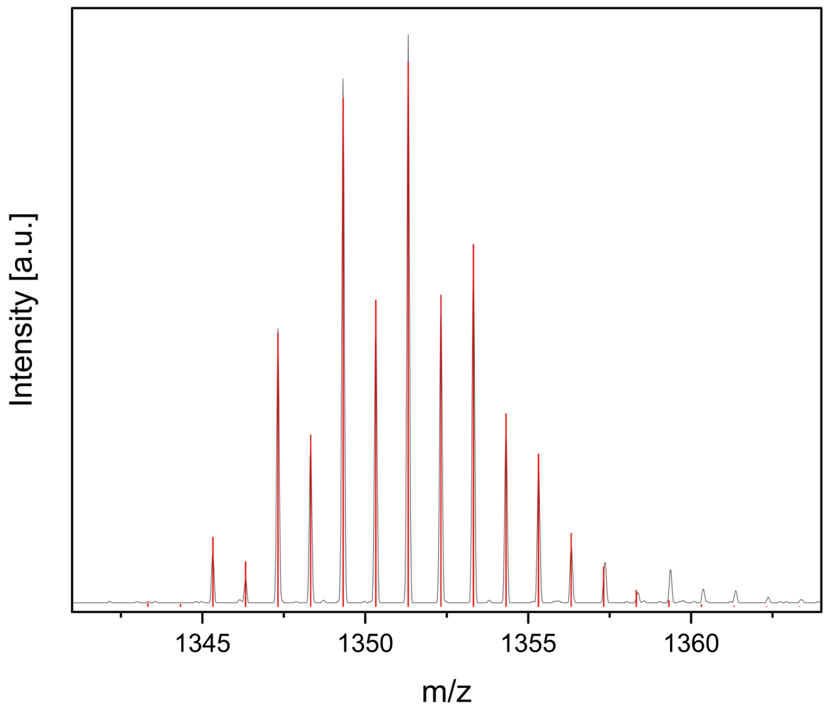

Figure S84 Cutout of the LIFDI mass spectrum of the reaction of $FeCl_2$ with GaTMP in THF in presence of Mg powder and H_2 after a reaction time of 18 h showing the isotopic pattern of $[Fe_2(GaTMP)_6Cl]^+$ (*m/z* = 1351.1988, black) and the theoretically predicted isotopic pattern of $[Fe_2(GaTMP)_6Cl]^+$ (calc.: 1351.31911, red).

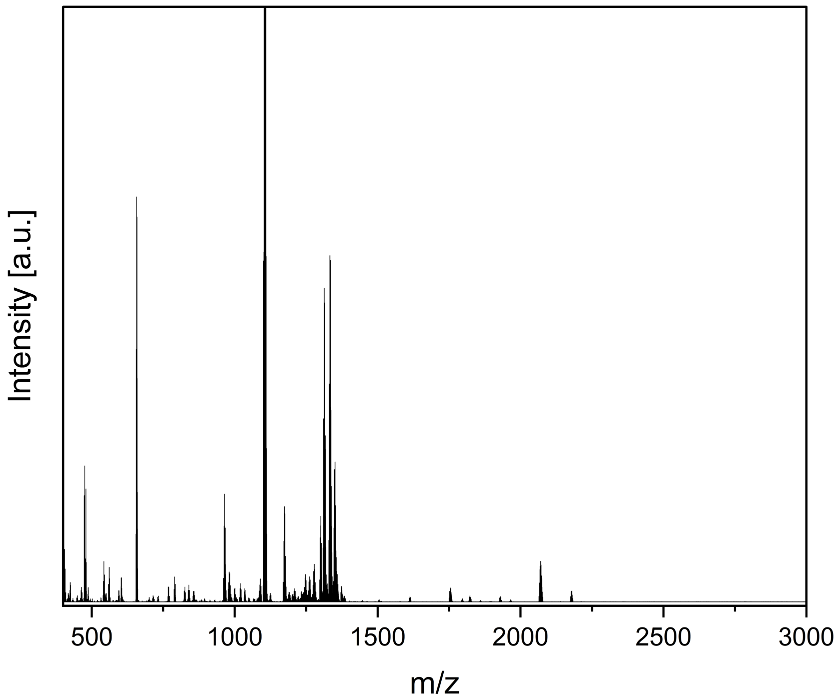

Figure S85 LIFDI mass spectrum of the reaction of $FeTMP_2$ with GaTMP in THF in presence of Mg powder and H_2 after a reaction time of 18 h.

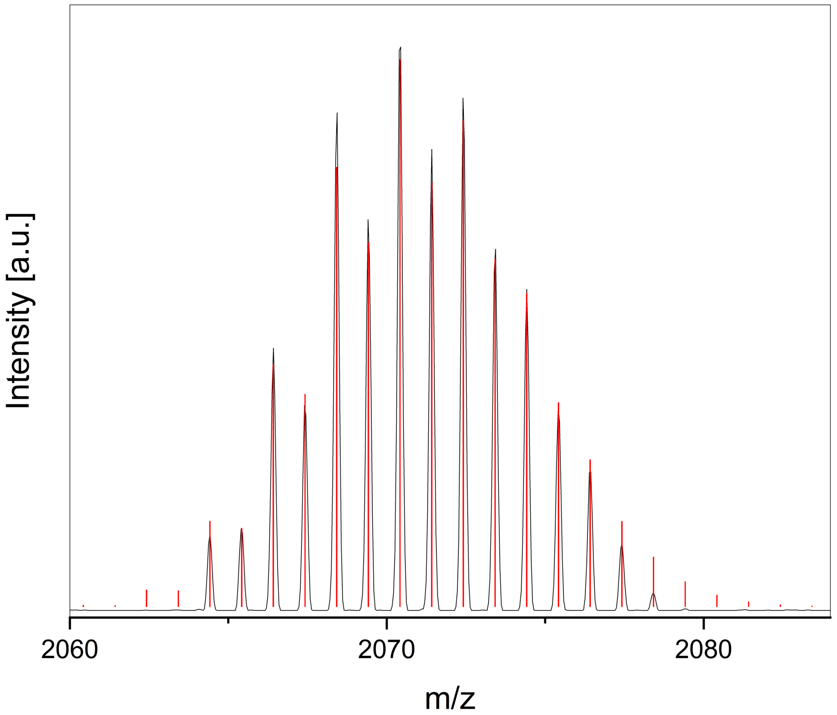

Figure S86 Cutout of the LIFDI mass spectrum of the reaction of $FeTMP_2$ with GaTMP in THF in presence of Mg powder and H_2 after a reaction time of 18 h showing the isotopic pattern of $[[Fe_2Ga](GaTMP)_9]^+$ (*m/z* = 2070.4181, black) and the theoretically predicted isotopic pattern of $[[Fe_2Ga](GaTMP)_9]^+$ (calc.: 2070.41696, red).

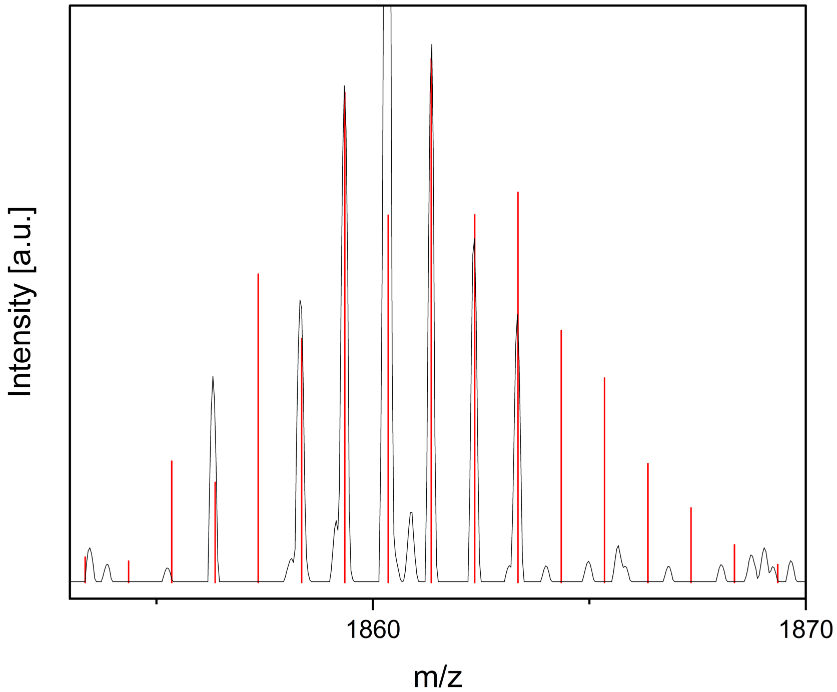

Figure S87 Cutout of the LIFDI mass spectrum of the reaction of $FeTMP_2$ with GaTMP in THF in presence of Mg powder and H_2 after a reaction time of 18 h showing the isotopic pattern of $[[Fe_2Ga](GaTMP)_8]^+$ (*m/z* = 1861.3501, black) and the theoretically predicted isotopic pattern of $[[Fe_2Ga](GaTMP)_8]^+$ (calc.: 1861.34746, red).

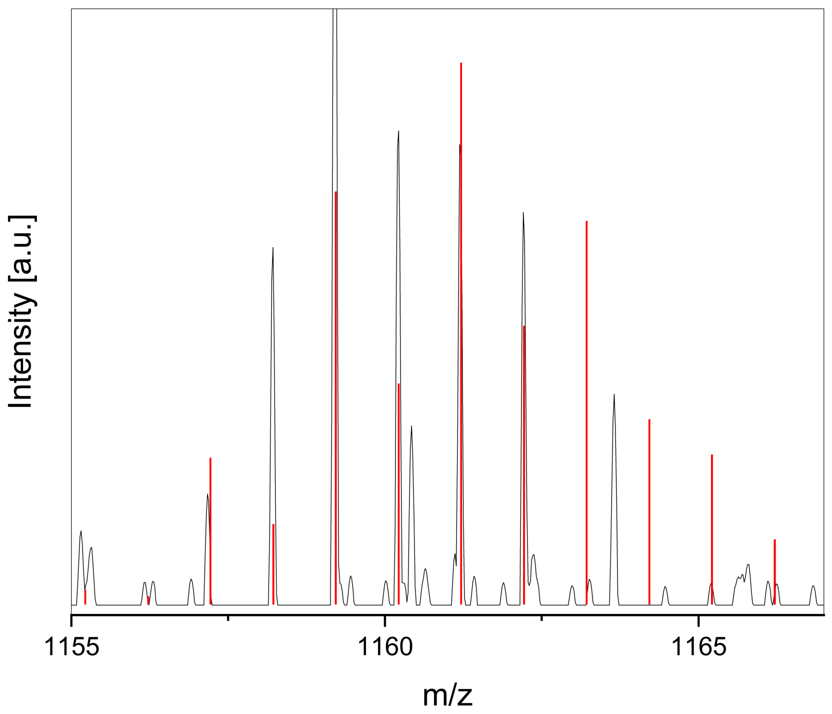

Figure S88 Cutout of the LIFDI mass spectrum of the reaction of $FeTMP_2$ with GaTMP in THF in presence of Mg powder and H_2 after a reaction time of 18 h showing the isotopic pattern of $[Fe_2(GaTMP)_5]^+$ (*m/z* = 1161.2014, black) and the theoretically predicted isotopic pattern of $[Fe_2(GaTMP)_5]^+$ (calc.: 1161.21511, red).

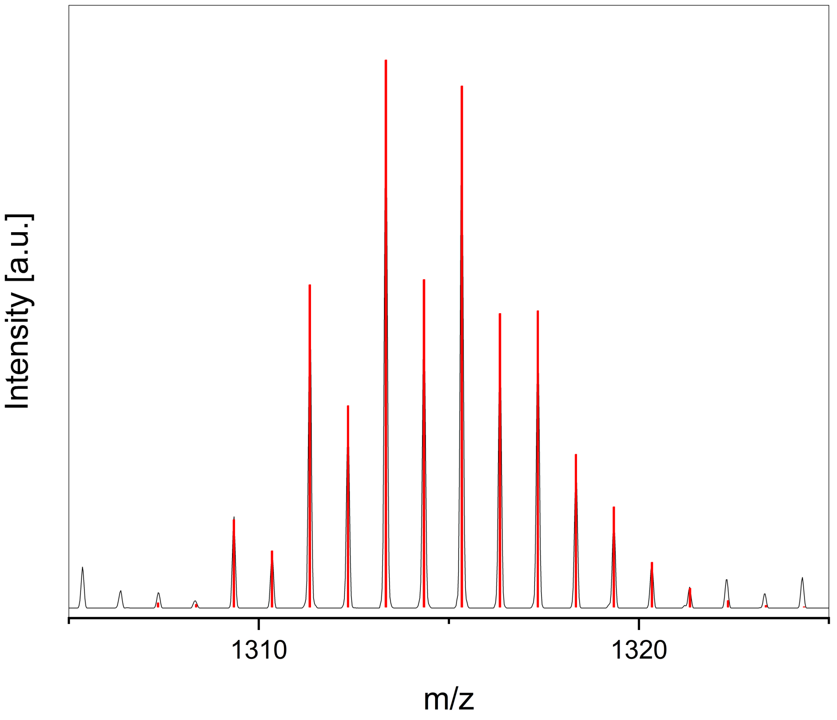

Figure S89 Cutout of the LIFDI mass spectrum of the reaction of $FeTMP_2$ with GaTMP in THF in presence of Mg powder and H_2 after a reaction time of 18 h showing the isotopic pattern of $[Fe(GaTMP)_6AntiH]^+$ (*m/z* = 1313.3403, black) and the theoretically predicted isotopic pattern of $[Fe(GaTMP)_6AntiH]^+$ (calc.: 1313.34185, red).

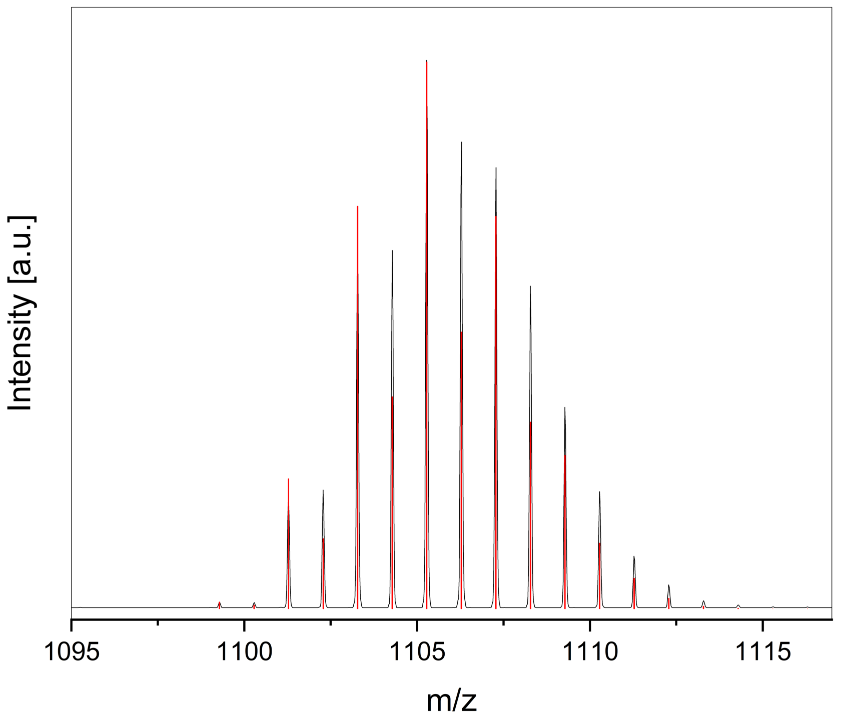

Figure S90 Cutout of the LIFDI mass spectrum of the reaction of $FeTMP_2$ with GaTMP in THF in presence of Mg powder and H_2 after a reaction time of 18 h showing the isotopic pattern of $[Fe(GaTMP)_5]^+$ (*m/z* = 1105.2798, black) and the theoretically predicted isotopic pattern of $[Fe(GaTMP)_5]^+$ (calc.: 1105.28017, red).

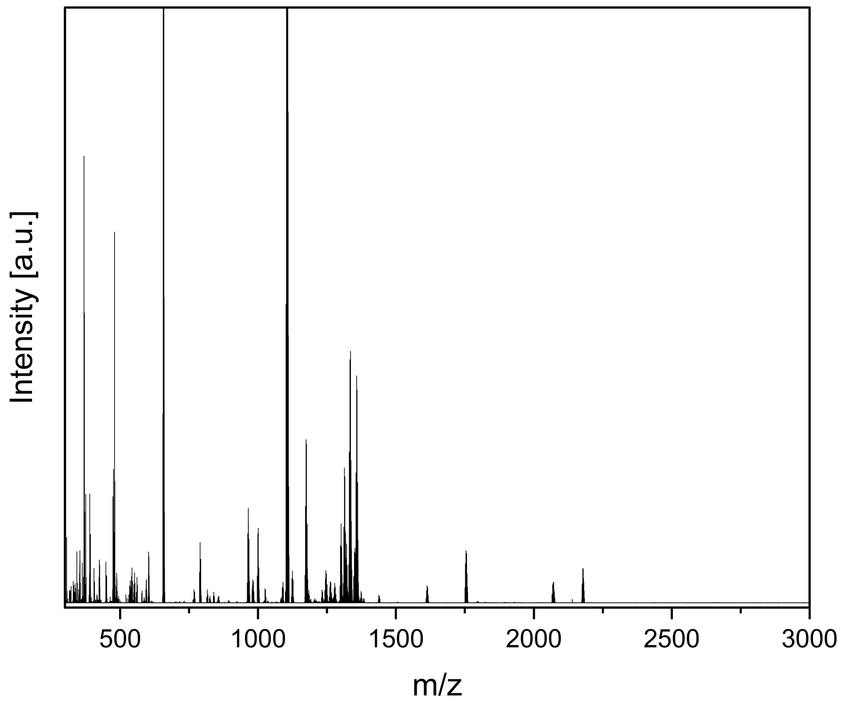

Figure S91 LIFDI mass spectrum of an additional reaction of $FeTMP_2$ with GaTMP in THF in presence of Mg powder and H_2 again after a reaction time of 18 h.

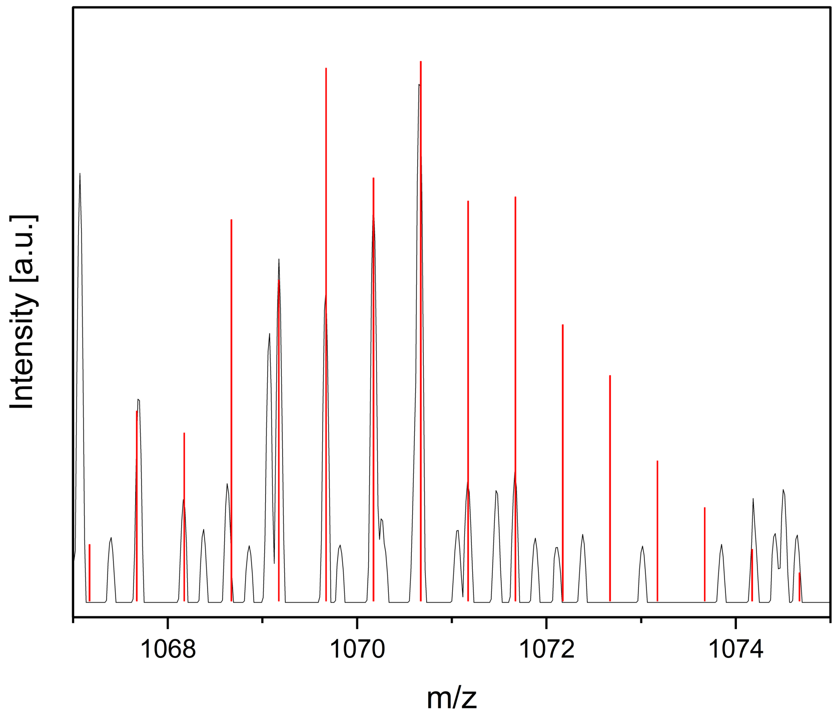

Figure S92 Cutout of the LIFDI mass spectrum of the reaction of $FeTMP_2$ with GaTMP in THF in presence of Mg powder and H_2 after a reaction time of 18 h showing the isotopic pattern of $[[Fe_2Ga_2](GaTMP)_9]^+$ (*m/z* = 1070.1731, black, twice positively charged) and the theoretically predicted isotopic pattern of $[[Fe_2Ga_2](GaTMP)_9]^+$ (calc.: 1070.17268, red).

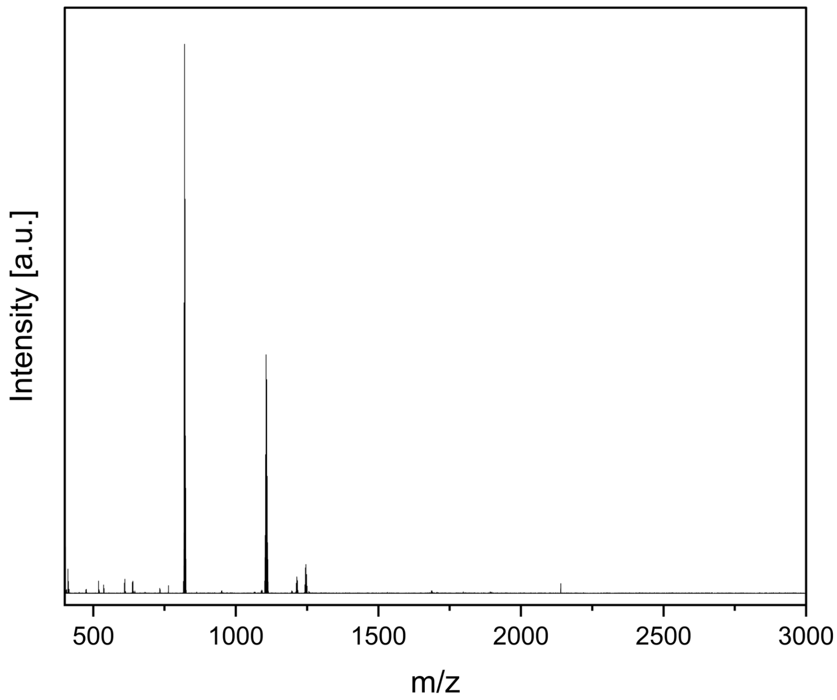

Figure S93 LIFDI mass spectrum of the reaction of $FeCl_2$ with GaTMP in THF in presence of H_2 only, after a reaction time of 18 h.

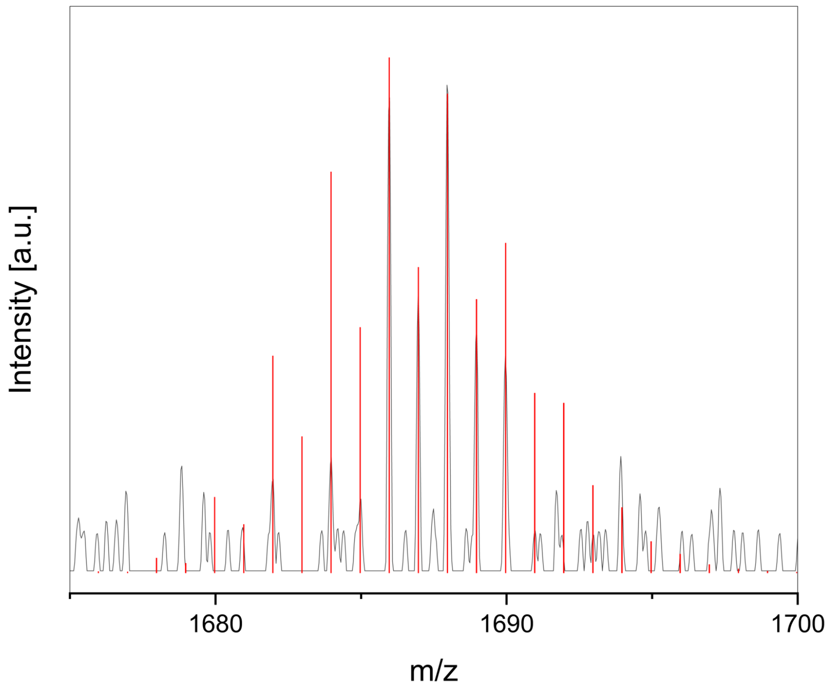

Figure S94 Cutout of the LIFDI mass spectrum of the reaction of $FeCl_2$ with GaTMP in THF in presence of H_2 only, after a reaction time of 18 h showing the isotopic pattern of [[Fe_2Ga](GaTMP)$_6$$Cl_3$]$^+$ (*m/z* = 1685.6951, black) and the theoretically predicted isotopic pattern of [[Fe_2Ga](GaTMP)$_6$$Cl_3$]$^+$ (calc.: 1685.96690, red).

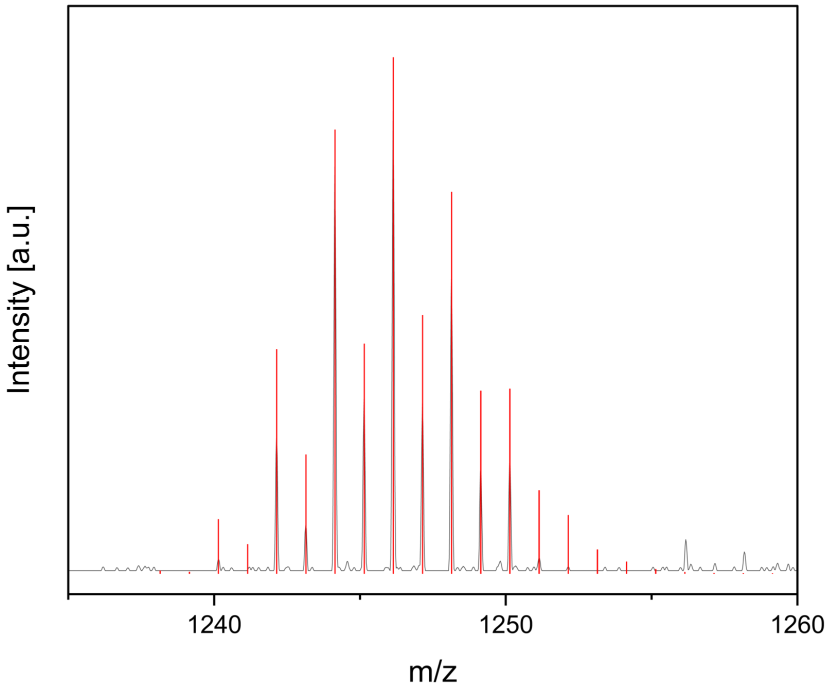

Figure S95 Cutout of the LIFDI mass spectrum of the reaction of $FeCl_2$ with GaTMP in THF in presence of H_2 only, after a reaction time of 18 h showing the isotopic pattern of [[FeGa](GaMP)$_5$$Cl_2$]$^+$ (*m/z* = 1246.1097, black) and the theoretically predicted isotopic pattern of [[FeGa](GaMP)$_5$$Cl_2$]$^+$ (calc.: 1264.14316, red).

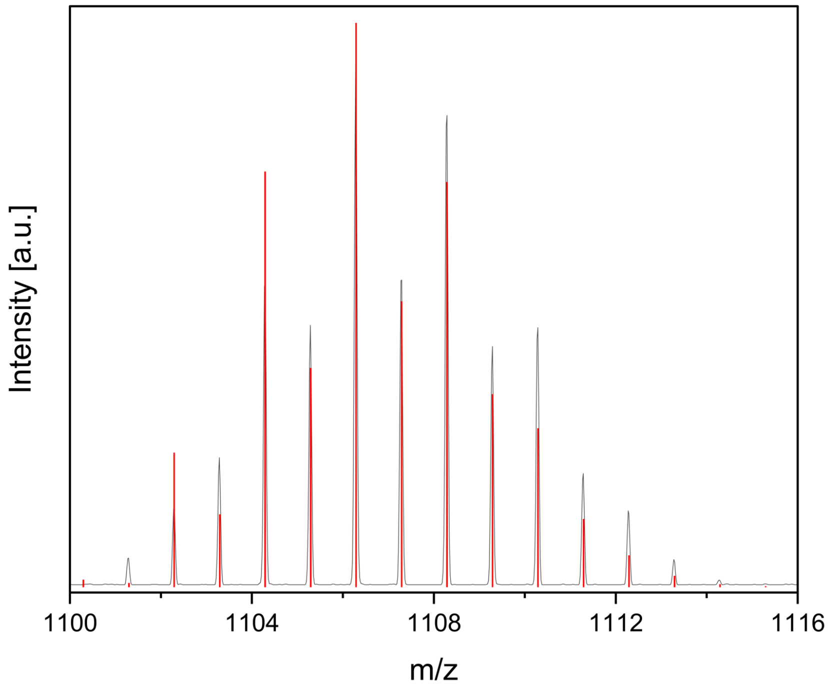

Figure S96 Cutout of the LIFDI mass spectrum of the reaction of $FeCl_2$ with GaTMP in THF in presence of H_2 only, after a reaction time of 18 h showing the isotopic pattern of $[Fe(GaMP)_5H]^+$ (*m/z* = 1106.2805, black) and the theoretically predicted isotopic pattern of $[Fe(GaMP)_5H]^+$ (calc.: 1106.28944, red).

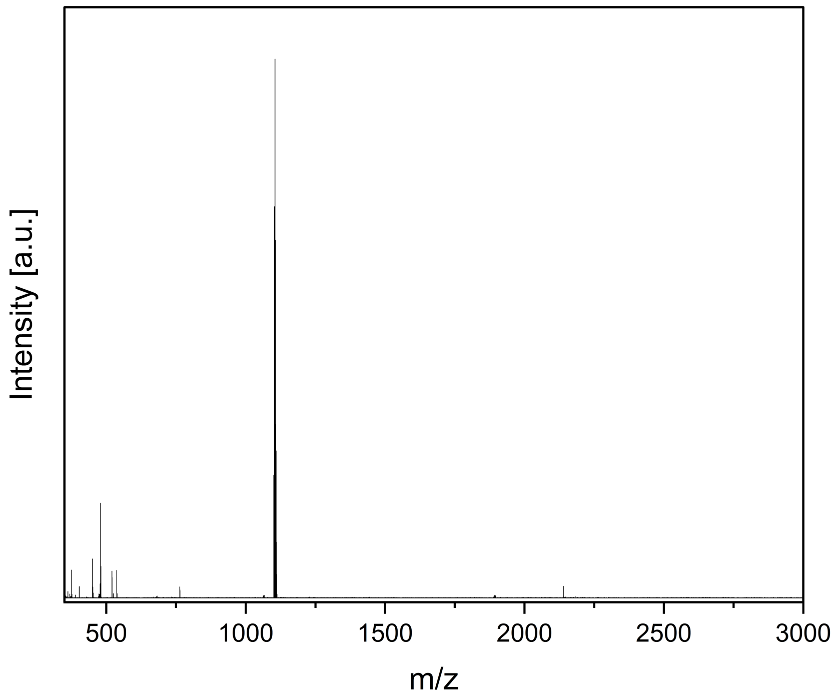

Figure S97 LIFDI mass spectrum of the reaction of $FeTMP_2$ with GaTMP in THF in presence of H_2 after a reaction time of 18 h.

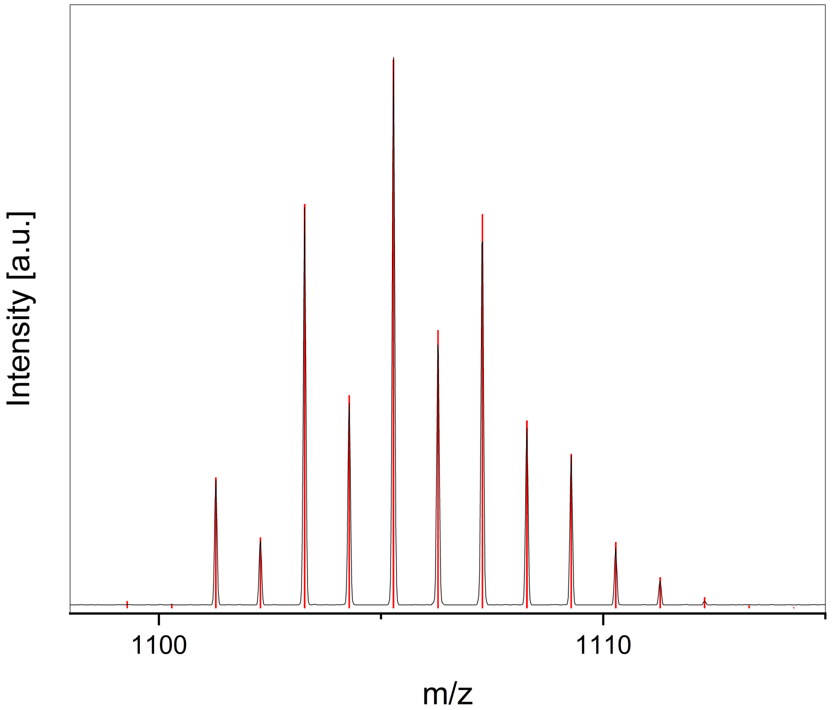

Figure S98 Cutout of the LIFDI mass spectrum of the reaction of $FeTMP_2$ with GaTMP in THF in presence of H_2 after a reaction time of 18 h showing the isotopic pattern of $[Fe(GaTMP)_5]^+$ (*m/z* = 1105.2779, black) and the theoretically predicted isotopic pattern of $[Fe(GaTMP)_5]^+$ (calc.: 1105.28017, red).

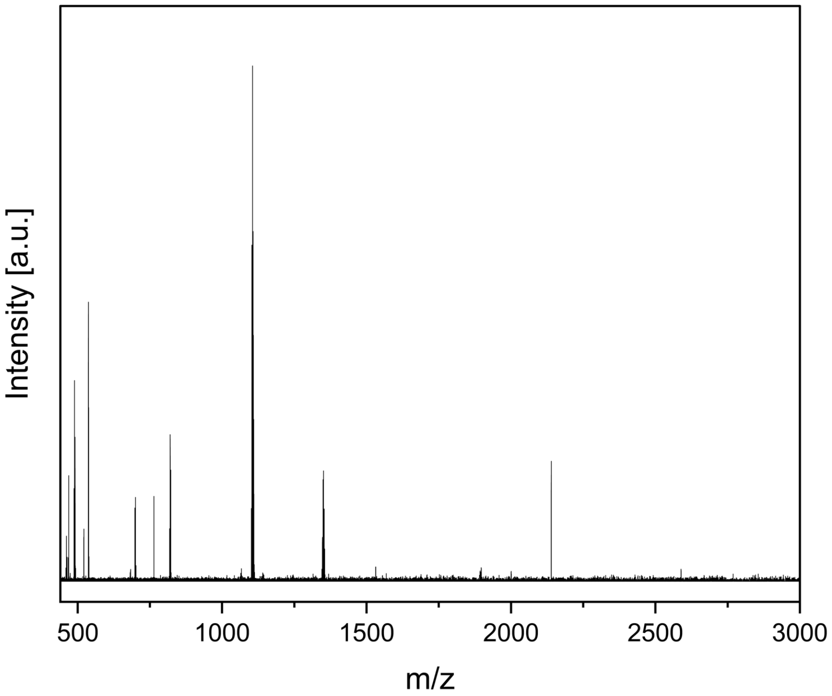

Figure S99 LIFDI mass spectrum of the reaction of $FeCl_2$ with GaTMP in THF in presence of Mg powder only, after a reaction time of 18 h.

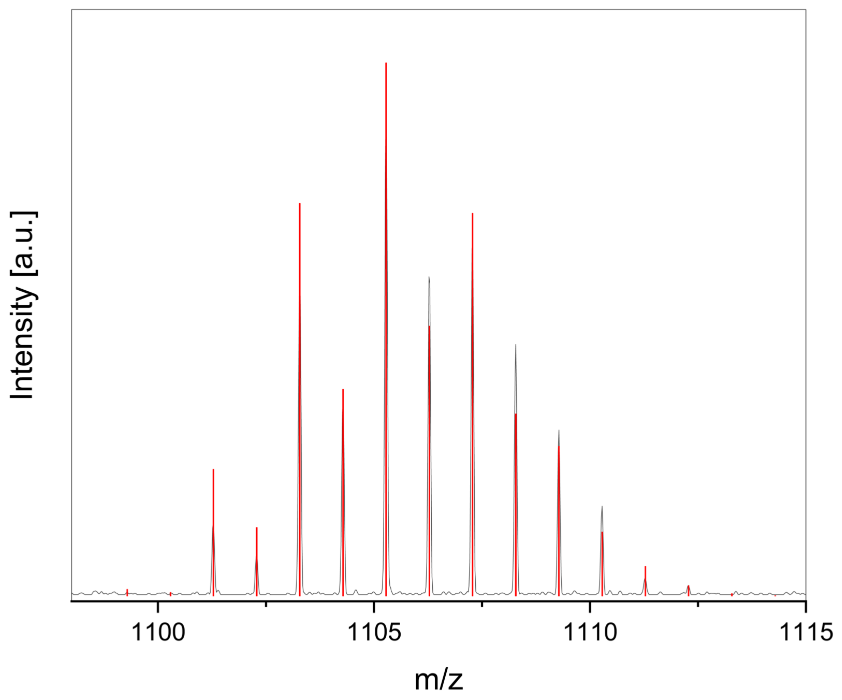

Figure S100 Cutout of the LIFDI mass spectrum of the reaction of $FeCl_2$ with GaTMP in THF in presence of Mg powder only, after a reaction time of 18 h showing the isotopic pattern of $[Fe(GaMP)_5]^+$ (*m/z* = 1105.2806, black) and the theoretically predicted isotopic pattern of $[Fe(GaMP)_5]^+$ (calc.: 1105.28161, red).

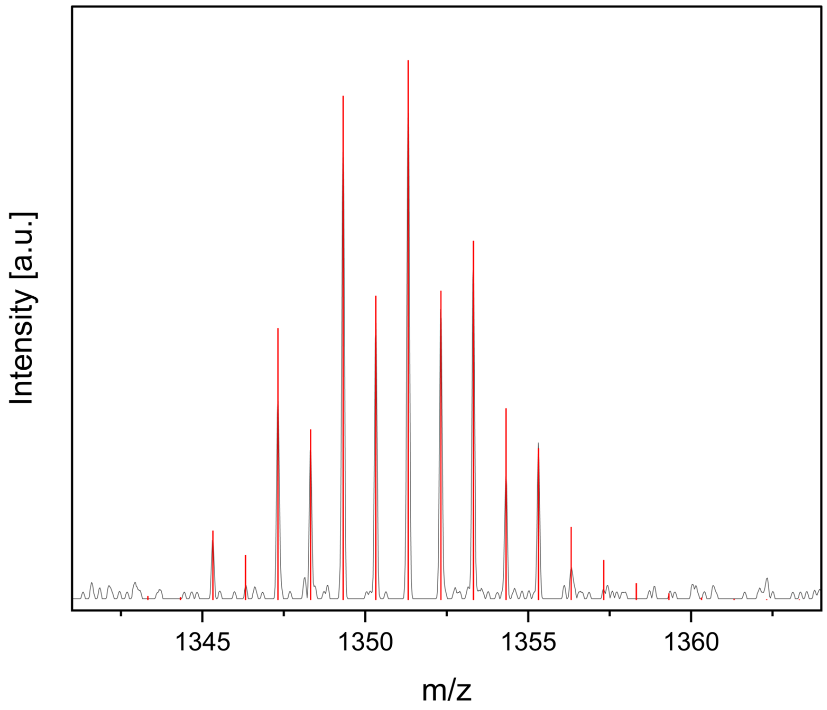

Figure S101 Cutout of the LIFDI mass spectrum of the reaction of $FeCl_2$ with GaTMP in THF in presence of Mg powder only, after a reaction time of 18 h showing the isotopic pattern of $[Fe(GaMP)_6Cl]^+$ (*m/z* = 1351.3203, black) and the theoretically predicted isotopic pattern of $[Fe(GaMP)_6Cl]^+$ (calc.: 1351.31911, red).

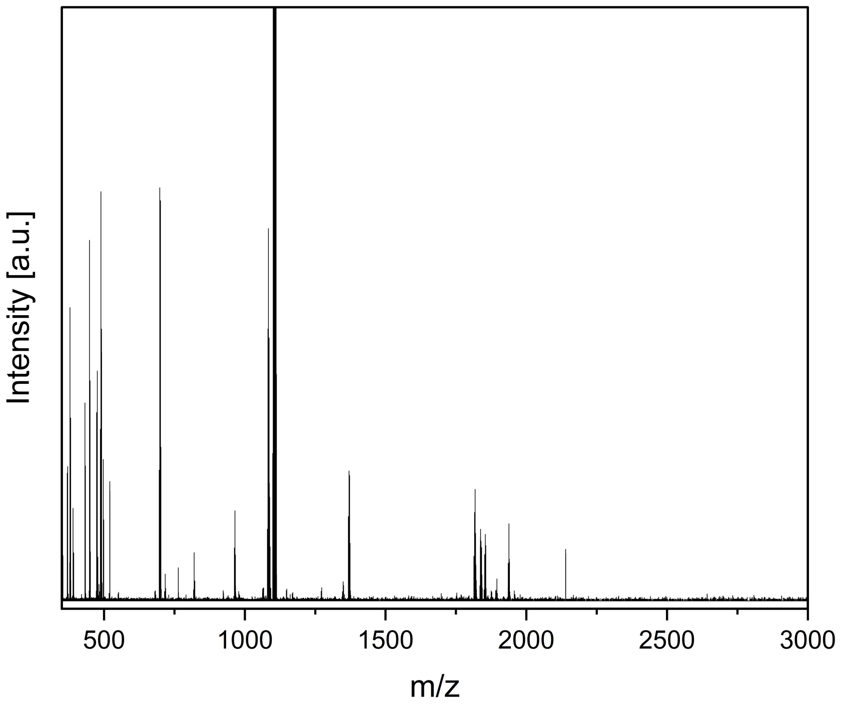

Figure S102 LIFDI mass spectrum of the reaction of $FeMes_2$ with GaTMP in THF in presence of Mg powder after a reaction time of 18 h.

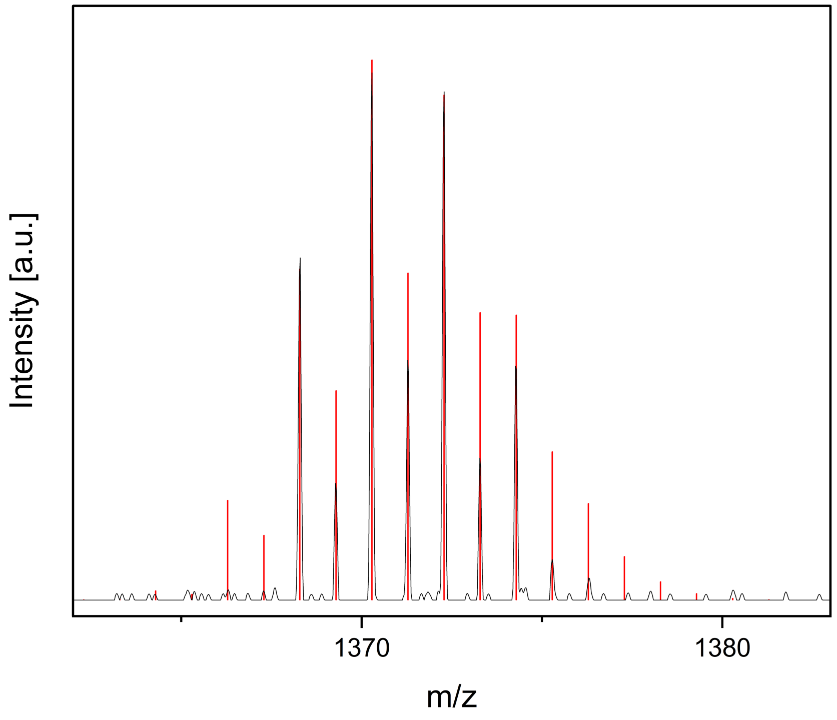

Figure S103 Cutout of the LIFDI mass spectrum of the reaction of $FeMes_2$ with GaTMP in THF in presence of Mg powder after a reaction time of 18 h showing the isotopic pattern of $[Fe_2(GaTMP)_6]^+$ (*m/z* = 1370.2822, black) and the theoretically predicted isotopic pattern of $[Fe_2(GaTMP)_6]^+$ (calc.: 1370.28462, red).

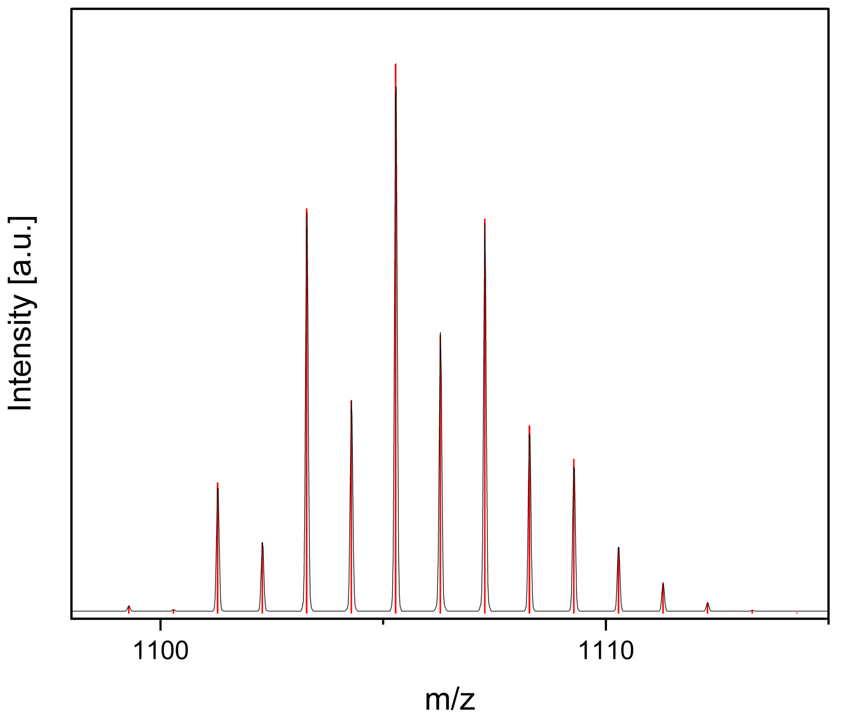

Figure S104 Cutout of the LIFDI mass spectrum of the reaction of $FeMes_2$ with GaTMP in THF in presence of Mg powder after a reaction time of 18 h showing the isotopic pattern of $[Fe(GaTMP)_5]^+$ (*m/z* = 1105.2850, black) and the theoretically predicted isotopic pattern of $[Fe(GaTMP)_5]^+$ (calc.: 1105.28017, red).

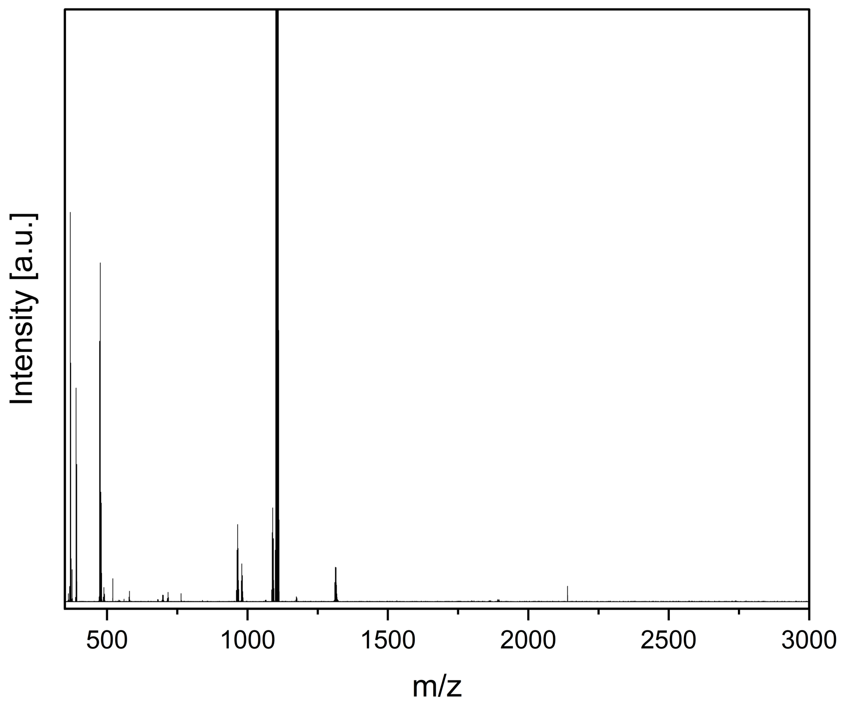

Figure S105 LIFDI mass spectrum of the reaction of $FeTMP_2$ with GaTMP in THF in presence of Mg powder after a reaction time of 18 h.

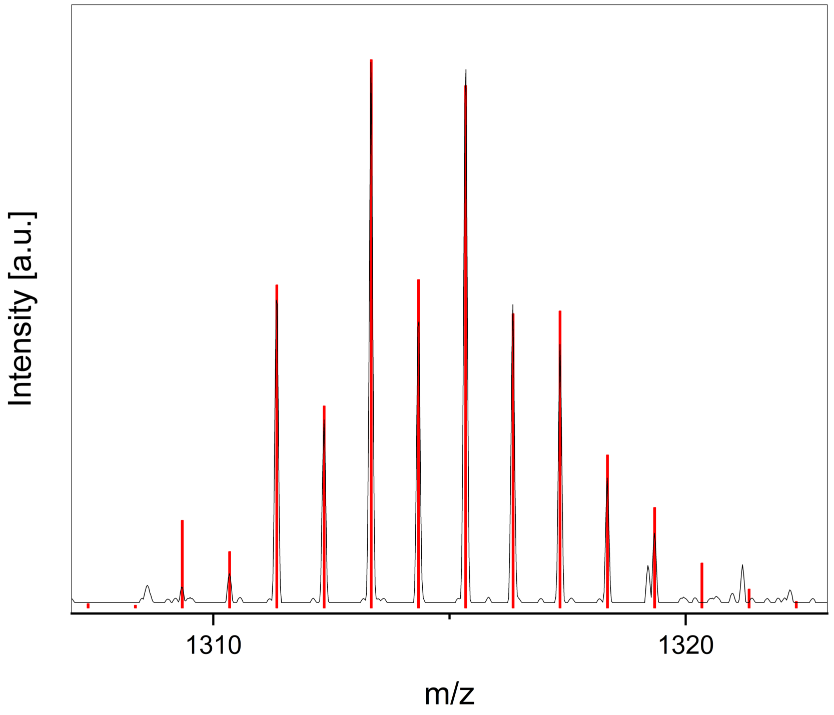

Figure S106 Cutout of the LIFDI mass spectrum of the reaction of $FeTMP_2$ with GaTMP in THF in presence of Mg powder after a reaction time of 18 h showing the isotopic pattern of $[Fe(GaTMP)_6AntiH]^+$ (*m/z* = 1313.3397, black) and the theoretically predicted isotopic pattern of $[Fe(GaTMP)_6AntiH]^+$ (calc.: 1313.34185, red).

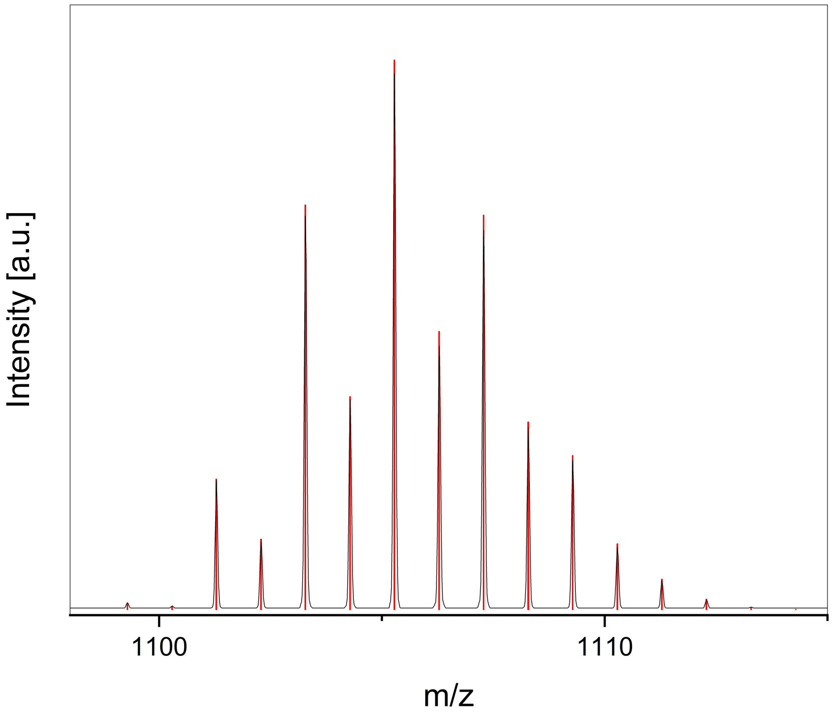

Figure S107 Cutout of the LIFDI mass spectrum of the reaction of $FeTMP_2$ with GaTMP in THF in presence of Mg powder after a reaction time of 18 h showing the isotopic pattern of $[Fe(GaTMP)_5]^+$ (*m/z* = 1105.2849, black) and the theoretically predicted isotopic pattern of $[Fe(GaTMP)_5]^+$ (calc.: 1105.28017, red).

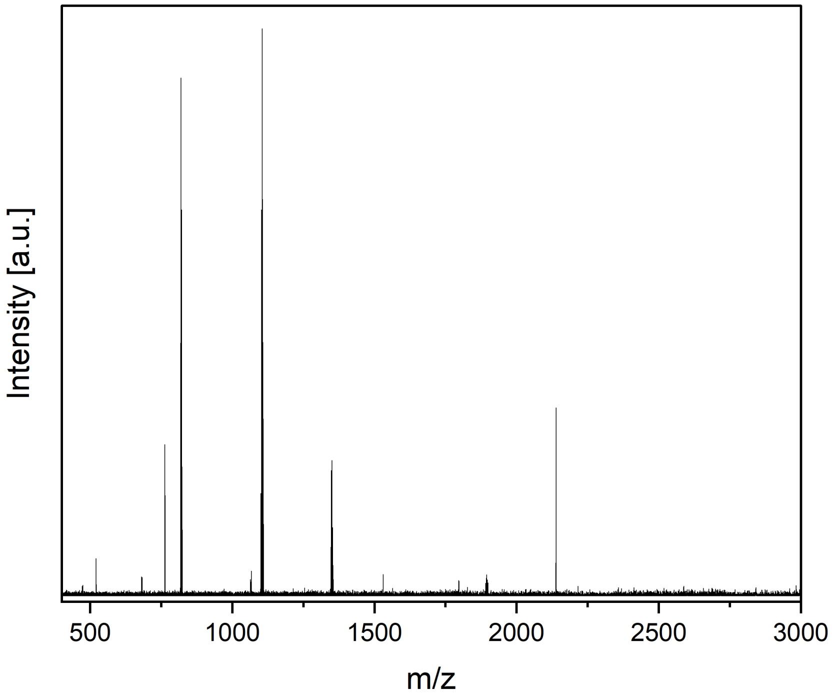

Figure S108 LIFDI mass spectrum of the reaction of $FeCl_2$ with GaTMP in THF in presence of no additives, after a reaction time of 18 h.

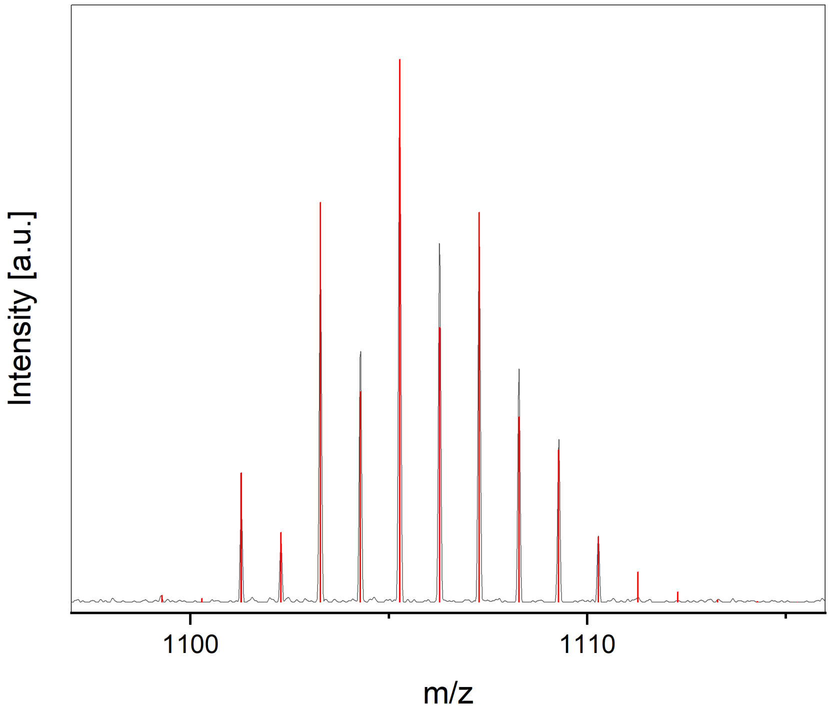

Figure S109 Cutout of the LIFDI mass spectrum of the reaction of $FeCl_2$ with GaTMP in THF in presence of no additives, after a reaction time of 18 h showing the isotopic pattern of $[Fe(GaMP)_5]^+$ (*m/z* = 1105.2829, black) and the theoretically predicted isotopic pattern of $[Fe(GaMP)_5]^+$ (calc.: 1105.28161, red).

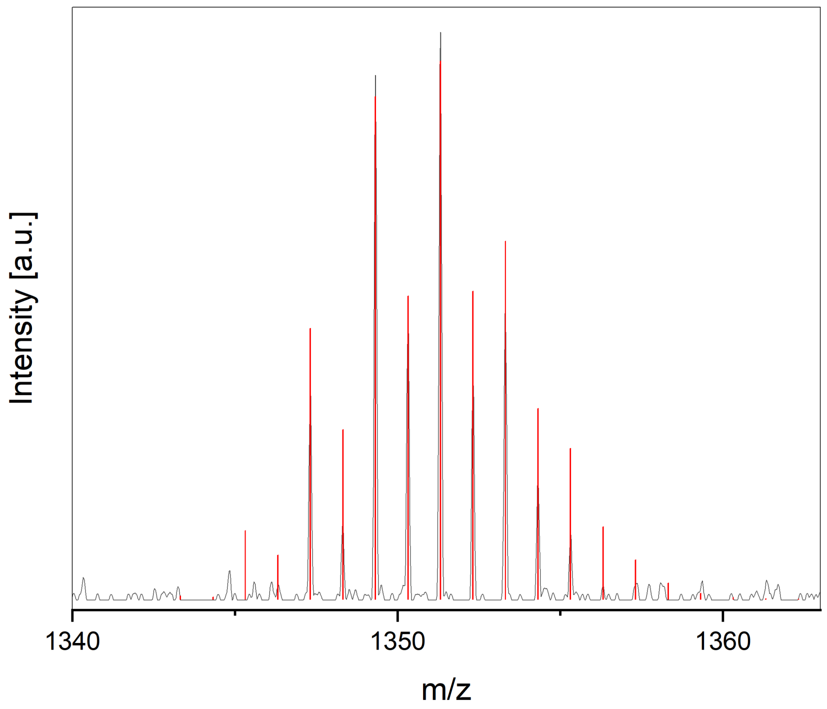

Figure S110 Cutout of the LIFDI mass spectrum of the reaction of $FeCl_2$ with GaTMP in THF in presence of no additives, after a reaction time of 18 h showing the isotopic pattern of $[Fe(GaMP)_6Cl]^+$ (*m/z* = 1351.3232, black) and the theoretically predicted isotopic pattern of $[Fe(GaMP)_6Cl]^+$ (calc.: 1351.31911, red).

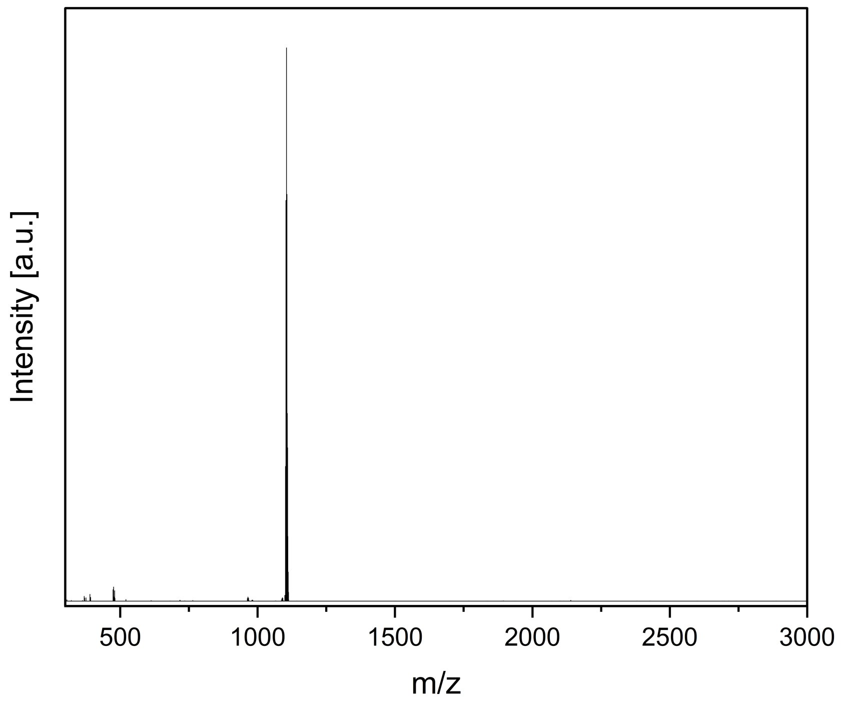

Figure S111 LIFDI mass spectrum of the reaction of $FeTMP_2$ with GaTMP in THF without an additive after a reaction time of 18 h.

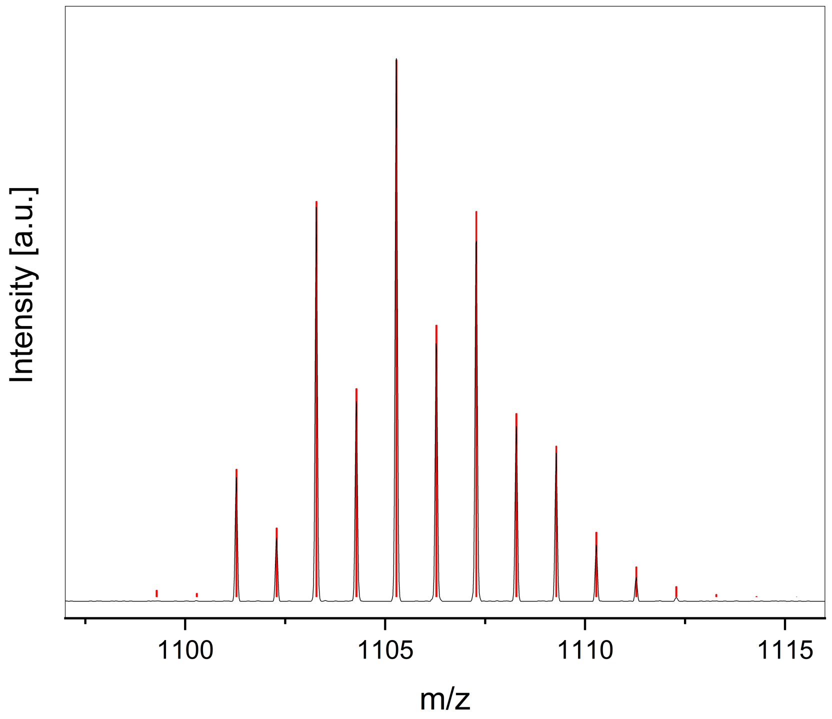

Figure S112 Cutout of the LIFDI mass spectrum of the reaction of $FeTMP_2$ with GaTMP in THF without an additive after a reaction time of 18 h showing the isotopic pattern of $[Fe(GaTMP)_5]^+$ (*m/z* = 1105.2778, black) and the theoretically predicted isotopic pattern of $[Fe(GaTMP)_5]^+$ (calc.: 1105.28017, red).

Extended bonding analysis of 1 from DFT calculations

The geometry of **1** was fully optimized, stating from its X-ray structure. Several hydride locations were tested and in fact, the by far most stable minimum was unsurprisingly found to have the hydride bonded in a terminal fashion to one of the core Ga atoms (Ga-H = 1.57 Å). Its position was also ascertained by the consistency between the computed Ga-H vibrational frequency 1854 cm^{-1} and its experimental counterpart (1844 cm^{-1}). In the deuterated relative **1-D**, these values shift to 1317 cm^{-1} (calc.) and 1259 cm^{-1} (exp.), respectively. Additionally, there is also a good matching between the computed ^{1}H -NMR chemical shift (6.4 ppm) of the hydride in **1**, and the observed ^{2}H-NMR chemical shift of the deuteride in **1-D** (6.60 and 6.49 ppm, see above).

In the DFT-optimized structure, the GaH unit lies closer to the Co_3 triangle than the bare Ga atom, rendering all the Co-Ga distances lying on this side shorter than those situated on the side of the bare Ga atom (Table S7). On the other hand, the Ga…Ga contacts discussed in the main text are significantly shorter on the bare Ga side (2.70 *vs*. 3.11 Å).

We assume that in the X-ray structure the GaH unit and the bare Ga atom both occupy statistically the two apices of the Co_3Ga_2 trigonal bipyramid, rendering the top and bottom sides of the whole cluster equivalent.

Not considering in a first step the six long Ga…Ga contacts linking each of the two core Ga atoms to their three GaTMP neighbors, it is *a priori* possible to describe the bonding in **1** from a localized point of view, with any of the Co-Ga and Co-Co contacts drawn in Figure 3 being a 2-electron/2-center bond. In such a view the Co atoms are 18-electron centers. With a total of 3x9 (3xCo) + 9x2 (9xGaTMP) + 1 (Ga) + 2 (GaH) = 48 electrons, **1** would belong to the large family of the triangular M_3L_n clusters having three 18-electron centers linked by three M-M single bonds (18x3 -2x3 = 48 electrons). This classical situation is schematized in Figure S113a which roughly sketches the major orbital interactions in a 48-electron triangular cluster comprising 18 metal-ligand 2-electron bonds. The Co-Co and Co-Ga Wiberg bond indices (WBIs, see Table S7) are not contradicting this description of single bonds, although the former should be considered as associated with particularly weak metal-metal σ bonds, barely stronger than for pure metallophilic interactions,[28] consistently with the fairly long Co-Co separations. The Kohn-Sham MO diagram of **1** is shown in Figure 4. It exhibits a significant HOMO-LUMO gap of 1.49 eV, in agreement with its stability and diamagnetism. Its HOMO can be considered as containing the lone pair on the bare gallium apex. The 16 orbitals lowest to the HOMO are a mixture of the 7 nitrogen lone pair orbitals with 9 metal combinations. Whereas one might expect three of them to be associated with the three Co-Co bonds discussed above (see Figure S113a), none of these occupied MOs contains any significant metal-metal bonding character. On the other hand, no vacant metal-dominant orbital with Co-Co antibonding character was found, but rather some ligand-based vacant

MOs with very weak additional metal-metal antibonding character. The picture which emerges then is a full occupation of the 9 metal-centered combinations shown in Figure S113a. Occupying the three M-M antibonding orbitals cancels the M-M bonds. Since the 48 electrons are still there, it means that now the ligand sphere is less electron-rich. Such a situation is sketched in Figure S113b. Note that it corresponds to a delocalized system since there are only 15 bonding electron pairs for 18 metal-ligand contacts. It can be explained when at least three ligand frontier orbitals lie at higher energy than the nd metal AOs. This is what happens in **1** where the three frontier orbitals of the HGa and Ga ligands and the $4p_{\sigma}$(Ga) OAs of the Co_2-bridging GaTMP ligands lie at high energy. As a result, the nine M-L bonding orbitals of dominant ligand character derive from the σ lone pairs of the nine GaTMP ligands. The computed natural atomic charges (Table S8) are consistent with a non-negligible polar character of the Co-GaTMP bonds. Quantum theory of atoms in molecules (QTAIM, see computational details) calculations were also performed to characterize the bonds in **1** and in fact bond critical points (bcps) were found for the 3 Co-Co and the 18 Co-Ga contacts. However, the Co-Co bcp descriptors (Table S9) are consistent with weak Co-Co covalent bonding. Following the Co-Ga distance trends, the in-plane Co-GaTMP bonds are significantly weaker than the other ones. The Co-Ga bonds within the Co_3Ga_2 bipyramidal core are even weaker, especially those involving the bare Ga atom.

In conclusion, it is to be mentioned that there is a continuum linking the two limit situations sketched in Figures S113a and S113b. Overall, our results indicate that the bonding in **1** lies between the two, but much closer to that described in Figure S113b. This delocalized bonding mode is further increased by the existence of three Ga_{bare}…GaTMP long contacts which are built at the expense of Co-Ga_{bare} and Co-GaTMP (Ga_{bare} side) bonds, as discussed in the main text.

Finally, as mentioned by a reviewer, with its Co_3Ga_2 trigonal bipyramidal core, compound **1** could be at first glance viewed also as a *closo*-type Wade-Mingos cluster of mixed metal/main group character. Indeed, its corresponding apparent number of skeletal electron pairs obtained from applying the regular Wade-Mingos counting rules, namely six, is consistent with such a *closo* arrangement. It should however be mentioned that Wade-Mingos clusters are expected to be composed of conical fragments, which is by far not the case of the Co atoms. Furthermore, the weak Co-Co bonding character is not in favor of a *closo* (*i.e.*, closed) trigonal bipyramidal description. Consistently, it was not possible to identify among the occupied Kohn-Sham orbitals of **1** the six bonding orbitals expected to hold the six skeletal electron pairs of a regular trigonal bipyramidal Wade-Mingos cluster. Thus, the *closo*-type description of **1** is to be ruled out.

Table S7 Selected average X-ray and DFT-optimized interatomic distances (in Å) for **1**, with corresponding Wiberg bond indices (WBIs).

	**X-ray**	**DFT**	**WBI**
Co-Co	2.671(3)	2.611	0.080
Co-Ga_{bare}	2.385(3)	2.526	0.262
Co-GaH		2.370	0.298
Co-GaTMP (in-plane)	2.350(2)	2.312	0.302
Co-GaTMP (Ga_{bare} side)	2.177(2)	2.204	0.357
Co-GaTMP (GaH side)		2.171	0.384
Ga-H	-	1.566	0.704
Ga_{bare}...GaTMP	2.924(3)	2.701	0.440
HGa...GaTMP		3.109	0.208

Table S8 Atomic charges computed from natural population analysis (NPA) and within the QTAIM framework.

	**Co**	**Ga_{bare}**	**Ga(H)**	**Ga(TMP) (in-plane)**	**Ga(TMP) (Ga_{bare} side)**	**Ga(TMP) (GaH side)**
**NPA**	-0.98	-0.72	-0.01	0.87	1.13	1.14
**QTAIM**	-0.48	0.02	0.50	0.70	0.63	0.65

Table S9 Topological bond critical points (BCP)s descriptors in **1**. ρ , $\nabla^2\rho$, H, V, G are the electron density, Laplacian of the ρ density, energy density, potential energy density, and kinetic energy density values at the bcp, respectively. δ is the delocalization index. All values in a.u.

	**Co-Co**	**Co-Ga$_{bare}$**	**Co-GaH**	**Co-GaTMP (in-plane)**	**Co-GaTMP (Ga$_{bare}$ side)**	**Co-GaTMP (GaH side)**	**Ga$_{bare}$...GaTMP**
**ρ**	0.046	0.048	0.061	0.067	0.080	0.085	0.042
**$\nabla^2\rho$**	0.055	0.051	0.072	0.089	0.129	0.147	0.026
**H**	-0.012	-0.014	-0.021	-0.025	-0.032	-0.035	-0.012
**V**	-0.038	-0.041	-0.061	-0.071	-0.094	-0.106	-0.031
**|V|/G**	1.47	1.52	1.54	1.52	1.55	1.49	1.66
**δ**	0.424	0.451	0.573	0.690	0.947	1.085	0.440

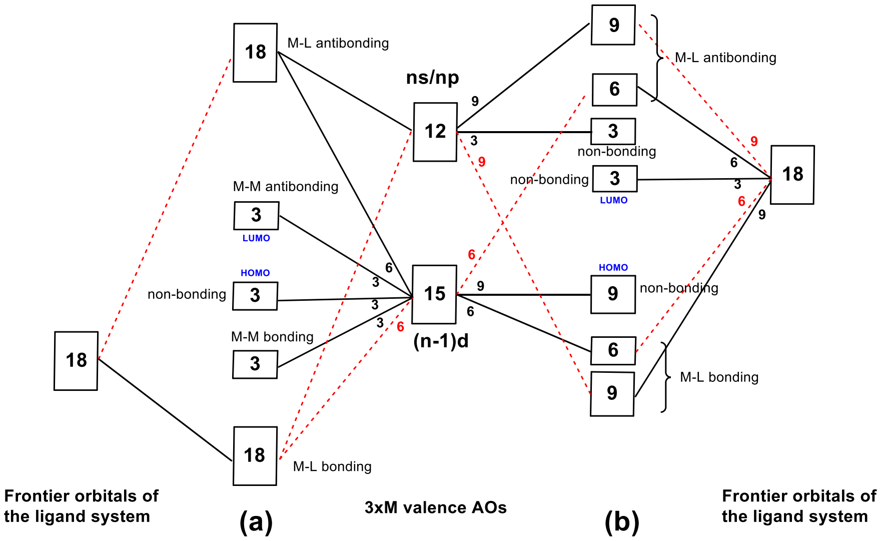

Figure S113 Simplified one-to-one orbital interaction diagram for a 48-electron triangular M_3L_n complex having 18 M-L bonding contacts: (a) The regular situation with three localized M-M bonds and 18 localized M-L bonds; (b) The limit case with no M-M bond and 15 bonding electron pairs associated with the 18 M-L bonding contacts. Solid black lines: Major contribution. Dotted red lines: Minor contribution.

References

[1] P. Jutzi, B. Neumann, G. Reumann, H.-G. Stammler, „Pentamethylcyclopentadienylgallium (Cp* Ga): Alternative synthesis and application as a terminal and bridging ligand in the chemistry of chromium, iron, cobalt, and nickel“ *Organometallics* **1998**, *17*, 1305-1314.

[2] M. Muhr, H. Liang, L. Allmendinger, R. Bühler, F. E. Napoli, D. Ukaj, M. Cokoja, C. Jandl, S. Kahlal, J. Y. Saillard, „Catalytic Alkyne Semihydrogenation with Polyhydride Ni/Ga Clusters“ *Angew. Chem. Int. Ed.* **2023**, *62*, e202308790.

[3] M. Muhr, P. Heiß, M. Schütz, R. Bühler, C. Gemel, M. H. Linden, H. B. Linden, R. A. Fischer, „Enabling LIFDI-MS measurements of highly air sensitive organometallic compounds: a combined MS/glovebox technique“ *Dalton Trans.* **2021**, *50*, 9031-9036.

[4] M. Loos, C. Gerber, F. Corona, J. Hollender, H. Singer, „Accelerated isotope fine structure calculation using pruned transition trees“ *Analytical chemistry* **2015**, *87*, 5738-5744.

[5] P. J. Dyson, B. F. Johnson, J. S. McIndoe, P. R. Langridge-Smith, „Energy-dependent electrospray ionisation mass spectrometry: applications in transition metal carbonyl chemistry“ *Rapid Communications in Mass Spectrometry* **2000**, *14*, 311-313.

[6] J. K. Diedrich, A. F. Pinto, J. R. Yates III, „Energy dependence of HCD on peptide fragmentation: stepped collisional energy finds the sweet spot“ *Journal of the American Society for Mass Spectrometry* **2013**, *24*, 1690-1699.

[7] R. Bühler, M. Schütz, K. F. Andriani, M. G. Quiles, J. P. A. de Mendonça, V. K. Ocampo-Restrepo, J. Stephan, S. Ling, S. Kahlal, J.-Y. Saillard, „A living library concept to capture the dynamics and reactivity of mixed-metal clusters for catalysis“ *Nature Chemistry* **2025**, 1-7.

[8] H. Kaesz, R. Saillant, „Hydride complexes of the transition metals“ *Chem. Rev.* **1972**, *72*, 231-281.

[9] J. Weßing, C. Göbel, B. Weber, C. Gemel, R. A. Fischer, „Diverse Reactivity of ECp*(E= Al, Ga) toward Low-Coordinate Transition Metal Amides [TM (N (SiMe3) 2) 2](TM= Fe, Co, Zn): Insertion, Cp* Transfer, and Orthometalation“ *Inorg. Chem.* **2017**, *56*, 3517-3525.

[10] J. J. Schneider, C. Krüger, M. Nolte, I. Abraham, T. S. Ertel, H. Bertagnolli, „[(Cp** CoH) 2-μ-{η2: η2-cis-μ-[(C2H5) 2Al–CH= CH–Al (C2H5) 2]}] and [{Cp*(η2-C2H4) CoAl (C2H5)} 2]: Synthesis and Structure of Unusual Co–Al Cluster Compounds“ *Angew. Chem. Int. Ed.* **1995**, *33*, 2435-2437.

[11] T. Bollermann, A. Puls, C. Gemel, T. Cadenbach, R. A. Fischer, „Reactions of cationic transition metal acetonitrile complexes [M (CH 3 CN) n] m+ with GaCp*: novel gallium complexes of iron, cobalt, copper and silver“ *Dalton Trans.* **2009**, 1372-1377.

[12] P. Jutzi, B. Neumann, L. O. Schebaum, A. Stammler, H.-G. Stammler, „Steric demand of the Cp* Ga ligand: synthesis and structure of Ni (Cp* Ga) 4 and of cis-M (Cp* Ga) 2 (CO) 4 (M= Cr, Mo)“ *Organometallics* **1999**, *18*, 4462-4464.

[13] C. Gemel, T. Steinke, D. Weiss, M. Cokoja, M. Winter, R. A. Fischer, „[M (GaCp*) 4](M= Pd, Pt) as Building Blocks for Dimeric Homoleptic Cluster Compounds of the Type [MPt (GaCp*) 5]“ *Organometallics* **2003**, *22*, 2705-2710.

[14] A. Kempter, C. Gemel, T. Cadenbach, R. A. Fischer, „Synthesis and Structure of New Compounds with Zn− Ga Bonds: Insertion of the Gallium (I) Bisimidinate Ga (DDP) into Zn− X (X= CH3, Cl) and the Homoleptic Complex Cation [Zn (GaCp*) 4] 2+“ *Inorg. Chem.* **2007**, *46*, 9481-9487.

[15] M. Muhr, J. Stephan, L. Staiger, K. Hemmer, M. Schütz, P. Heiß, C. Jandl, M. Cokoja, T. Kratky, S. Günther, „Assignment of individual structures from intermetalloid nickel gallium cluster ensembles“ *Commun. Chem.* **2024**, *7*, 29.

[16] C. L. Nivert, G. H. Williams, D. Seyferth, S. H. Strauss, „µ3-(Chloromethylidyne)-and µ3-[(Tert-Butoxycarbonyl) Methylidyne]-Tris (Tricarbonylcobalt)“ *Inorganic Syntheses* **1980**, *20*, 234-237.

[17] M. E. Smith, R. A. Andersen, „Preparation of the Paramagnetic Hydrides (Me5C5) 3M3 (µ3-CH)(µ-H)(M= Ni, Co)“ *Organometallics* **1996**, *15*, 2680-2682.

[18] S. Guo, R. Hauptmann, S. Losi, P. Zanello, J. J. Schneider, „Organometallic Heterocubane Clusters with Co–O and Co–S Framework“ *Journal of Cluster Science* **2007**, *18*, 237-251.

[19] G. H. Robinson, „Gallanes, gallenes, cyclogallenes, and gallynes: organometallic chemistry about the gallium− gallium bond“ *Acc. Chem. Res.* **1999**, *32*, 773-782.

[20] A. v. Bondi, „van der Waals Volumes and Radii“ *J. Phys. Chem.* **1964**, *68*, 441-451.

[21] H. Gao, C. Yuan, H. Chen, A. Dong, P. Gao, G. Hou, „Surface gallium hydride on Ga2O3 polymorphs: A comparative solid-state NMR study“ *Chinese Journal of Structural Chemistry* **2025**, *44*, 100561.

[22] H. Chen, P. Gao, Z. Liu, L. Liang, Q. Han, Z. Wang, K. Chen, Z. Zhao, M. Guo, X. Liu, „Direct detection of reactive gallium-hydride species on the Ga2O3 surface via solid-state NMR spectroscopy“ *J. Am. Chem. Soc.* **2022**, *144*, 17365-17375.

[23] P. Leung, P. Coppens, „Experimental charge density study of dicobalt octacarbonyl and comparison with theory“ *Acta Crystallographica Section B: Structural Science* **1983**, *39*, 535-542.

[24] A. Seifert, G. Linti, „2,2,6,6-Tetramethylpiperidinogallium as a Terminal and Bridging Ligand in Homo- and Heteroleptic Chromium, Nickel, and Cobalt Complexes“ *Inorg. Chem.* **2008**, *47*, 11398-11404.

[25] J. Dewar, H. Owen Jones, „The physical and chemical properties of iron carbonyl“ *Proceedings of the Royal Society of London. Series A, Containing Papers of a Mathematical and Physical Character* **1905**, *76*, 558-577.

[26] H. Powell, R. Ewens, „64. The crystal structure of iron enneacarbonyl“ *Journal of the Chemical Society (Resumed)* **1939**, 286-292.

[27] G. Linti, W. Köstler, „The tris (trimethylsilyl) silylgallium group as a building block in gallium–iron clusters“ *Chem. Eur. J.* **1998**, *4*, 942-949.

[28] R. Bühler, R. M. Wolf, C. Gemel, J. Stephan, S. N. Deger, S. Kahlal, R. A. Fischer, J.-Y. Saillard, „Cuprophilic Interactions in Polymeric [Cu10O2 (Mes) 6] n“ *Inorg. Chem.* **2024**, *63*, 17617-17625.

Crystallographic References

C1. *APEX4*, Version 2021, Bruker AXS Inc., Madison, Wisconsin, USA, 2021.

C2. *CrysAlisPro*, 1.171.43.144a, 2024, Rigaku OD.

C3. *SAINT*, Version 2021, Bruker AXS Inc., Madison, Wisconsin, USA, 2021.

C4. *SADABS*, Version 2021, Bruker AXS Inc., Madison, Wisconsin, USA, 2021.

C5. G. M. Sheldrick, *Acta Crystallogr. Sect. A*, 2015, **71**, 3–8.

C6. G. M. Sheldrick, *Acta Crystallogr. Sect. C*, 2015, **71**, 3–8.

C7. C. B. Hübschle, G. M. Sheldrick, B. Dittrich, *J. Appl. Cryst.*, 2011, **44**, 1281–1284.

C8. *International Tables for Crystallography*, Vol. C (Ed.: A. J. Wilson), Kluwer Academic Publishers, Dordrecht, The Netherlands, 1992, Tables 6.1.1.4 (pp. 500–502), 4.2.6.8 (pp. 219–222), and 4.2.4.2 (pp. 193–199).

C9. D. Kratzert, J. J. Holstein, I. Krossing, *J. Appl. Cryst.*, 2015, **48**, 933–938.

C10. A. L. Spek, *Acta Crystallogr. Sect. C*, 2015, **71**, 9–18.

C11. C. F. Macrae, I. J. Bruno, J. A. Chisholm, P. R. Edgington, P. McCabe, E. Pidcock, L. Rodriguez-Monge, R. Taylor, J. van de Streek, P. A. Wood, *J. Appl. Cryst.*, 2008, **41**, 466–470.

Computational References

A) G. te Velde, F. M. Bickelhaupt, E. J. Baerends, C. Fonseca Guerra, S. J. A. van Gisbergen, J. G. Snijders, T. Ziegler, *J. Comput. Chem.* **2001,** *22*, 931-967.

B) E. van Lenthe, E. J. Baerends, J. G. Snijders, *J. Chem. Phys.* **1993,** *99*, 4597-4610.

C) E. van Lenthe, E. J. Baerends, J. G. Snijders,. *J. Chem. Phys.* **1994,** *101*, 9783-9792.

D) S. Grimme, J. Antony, S. Ehrlich, H. Krieg, *J. Chem. Phys.* 2010, **132**, 154104.

E) E. van Lenthe, E. J. Baerends, *J. Comput. Chem.* **2003,** *24*, 1142-1156.

F) E. D. Glendening, J. K. Badenhoop, A. E. Reed, J. E. Carpenter, J. A. Bohmann, C. M. Morales, F. Weinhold, F. NBO 6.0, University of Wisconsin (Madison, WI, 2001, http://nbo6.chem.wisc.edu).

X) A. D. Becke, *Phys Rev A.* **1988**, *38*, 3098–3100.

Y) J. P. Perdew, *Phys Rev B.* **1986**, *33*, 8822–8824.

G) R. F. W. Bader, *Atoms in Molecules-A Quantum Theory*. Oxford University Press: Oxford, England, 1990.

H) J. I. Rodríguez, *J. Comput. Chem.* **2013,** *34*, 681-686.

I) J. I. Rodríguez, R. F. W. Bader, P. W. Ayers, C. Michel, A. W. Götz, C. Bo, *Chem. Phys. Lett.* **2009,** *472*, 149-152.

J) G. Schreckenbach, T. Ziegler, *J. Phys. Chem*. **1995**, *99*, 606-611.

K) S. K. Wolff, T. Ziegler, E. van Lenthe, E. J. Baerends, *J. Phy. Chem*. **1999**, *110*, 7689-7698.
